# Identification of two novel biomarkers of rectal carcinoma progression and prognosis via co-expression network analysis

**DOI:** 10.18632/oncotarget.18646

**Published:** 2017-06-27

**Authors:** Min Sun, Taojiao Sun, Zhongshi He, Bin Xiong

**Affiliations:** ^1^ Department of Oncology, Zhongnan Hospital of Wuhan University, Hubei Key Laboratory of Tumor Biological Behaviors & Hubei Cancer Clinical Study Center, Wuhan 430071, P.R. China; ^2^ Department of Stomatology, Zhongnan Hospital of Wuhan University, Wuhan 430071, P.R. China; ^3^ Department of Radiation and Medical Oncology, Zhongnan Hospital of Wuhan University, Wuhan 430071, P.R. China

**Keywords:** rectal cancer, The Cancer Genome Atlas, weighted gene co-expression network analysis, prognosis, disease progression

## Abstract

mRNA expression profiles provide important insights on a diversity of biological processes involved in rectal carcinoma (RC). Our aim was to comprehensively map complex interactions between the mRNA expression patterns and the clinical traits of RC. We employed the integrated analysis of five microarray datasets and The Cancer Genome Atlas rectal adenocarcinoma database to identify 2118 consensual differentially expressed genes (DEGs) in RC and adjacent normal tissue samples, and then applied weighted gene co-expression network analysis to parse DEGs and eight clinical traits in 66 eligible RC samples. A total of 16 co-expressed gene modules were identified. The green-yellow and salmon modules were most appropriate to the pathological stage (R = 0.36) and the overall survival (HR =13.534, *P* = 0.014), respectively. A diagnostic model of the five pathological stage hub genes (SCG3, SYP, CDK5R2, AP3B2, and RUNDC3A) provided a powerful classification accuracy between localized RC and non-localized RC. We also found increased Secretogranin III (SCG3) expression with higher pathological stage and poorer prognosis in the test and validation set. The increased Homer scaffolding protein 2 (HOMER2) expression with the favorable survival prediction efficiency significantly correlated with the markedly reduced overall survival of RC patients and the higher pathological stage during the test and validation set. Our findings indicate that the *SCG3* and *HOMER2* mRNA levels should be further evaluated as predictors of pathological stage and survival in patients with RC.

## INTRODUCTION

Accurate staging of rectal cancer (RC) is essential for carrying out precise therapies that increase the survival rate of patients [1]. Previous studies have found no differences in specific clinicopathological risk factors between patients with a high or low risk of RC progression [2]. Recent technological breakthroughs in genome-wide sequencing have shed new insights on deregulated mRNAs that have been identified and characterized in the past few years [3]. Genetic biomarkers for RC, especially for modules having a strong correlation between genes with similar expression patterns, to predict the pathological stage and survival outcome, which could help to understand disease pathogenesis and provide personalized treatment, have been rarely reported. However, previous research based on examining genetic mutations and different expression patterns associating with colorectal carcinogenesis has largely ignored the relationship between the genes and clinical characteristics [4, 5]. In addition, RC progression involves several critical stages, although most studies have only evaluated the differences between RC and adjacent normal tissue (ANT), regardless of the intermediate stages [6]. Therefore, there is an urgency to include stage and prognostic predictive modules to the current staging system, which could be achieved by combining information on the clinical characteristics and validated gene-specific biomarkers. For these reasons, we aimed to establish comprehensive mRNA expression patterns of the module genes and clinical traits, especially those relating to multi-stage disease progression which directly affects the prognosis through weighted gene co-expression network analysis (WGCNA) [7].

WGCNA results in the construction of free-scale gene co-expression networks that explore the relationships between the gene sets and clinical features [8]. WGCNA groups prerogative genes into modules based on their co-expression similarities and analogous functions across a population of samples [9–11].

In the present work, we carried out the pooled analysis of RC mRNA raw microarrays and the network level analysis of The Cancer Genome Atlas (TCGA) rectal adenocarcinoma (READ) data to decipher the relationships between the module genes and clinical traits. To the best of our knowledge, we are the first group to use meta-analysis and WGCNA to identify modules displaying a nominal evidence association with RC clinical traits. By characterizing module content and topology, we identified clinical traits, modules, and network concepts that play important roles in the regulation of RC at the level of the differentially expressed genes (DEGs). Moreover, to define the prognostic value of the cancer-specific module, which was related to tumor progression, further analysis was performed to validate candidate markers by combining survival analysis with an independent validation cohort.

## RESULTS

### Identification of consensual DEGs in training and test sets in rectal cancer patients

The flow chart outlining the methods used in this study is shown in Supplementary Figure 1. In contrast to colon cancer, there was very limited mRNA expression data relating to rectal cancer. We investigated and manually curated four public datasets (GSE12225, GSE34472, GSE35982, and GSE75548) as the training set (Supplementary Table 1) [12–15]**.**

**Figure 1:**
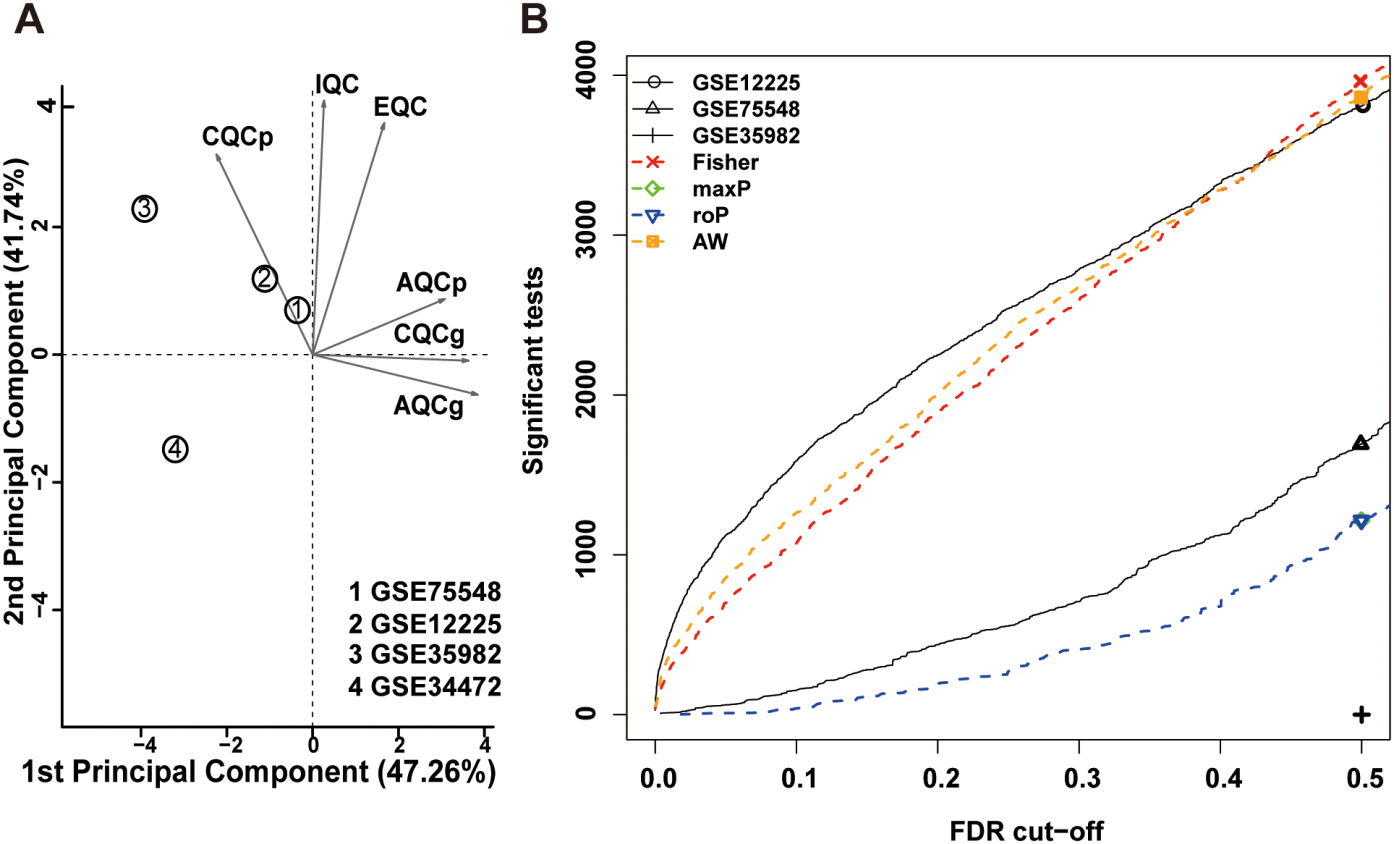
Meta-analysis of differentially expressed genes involved in rectal cancer by combining *P*-values **(A)** Principal component analysis (PCA) biplot of quality control measures in four RC studies. **(B)** The number of differentially expressed genes plotted as a function of false discovery rate (FDR) in the analysis of four different datasets and four different meta-analysis algorithms (maxP, Fisher roP and adaptively weighted statistic).

**Figure 2:**
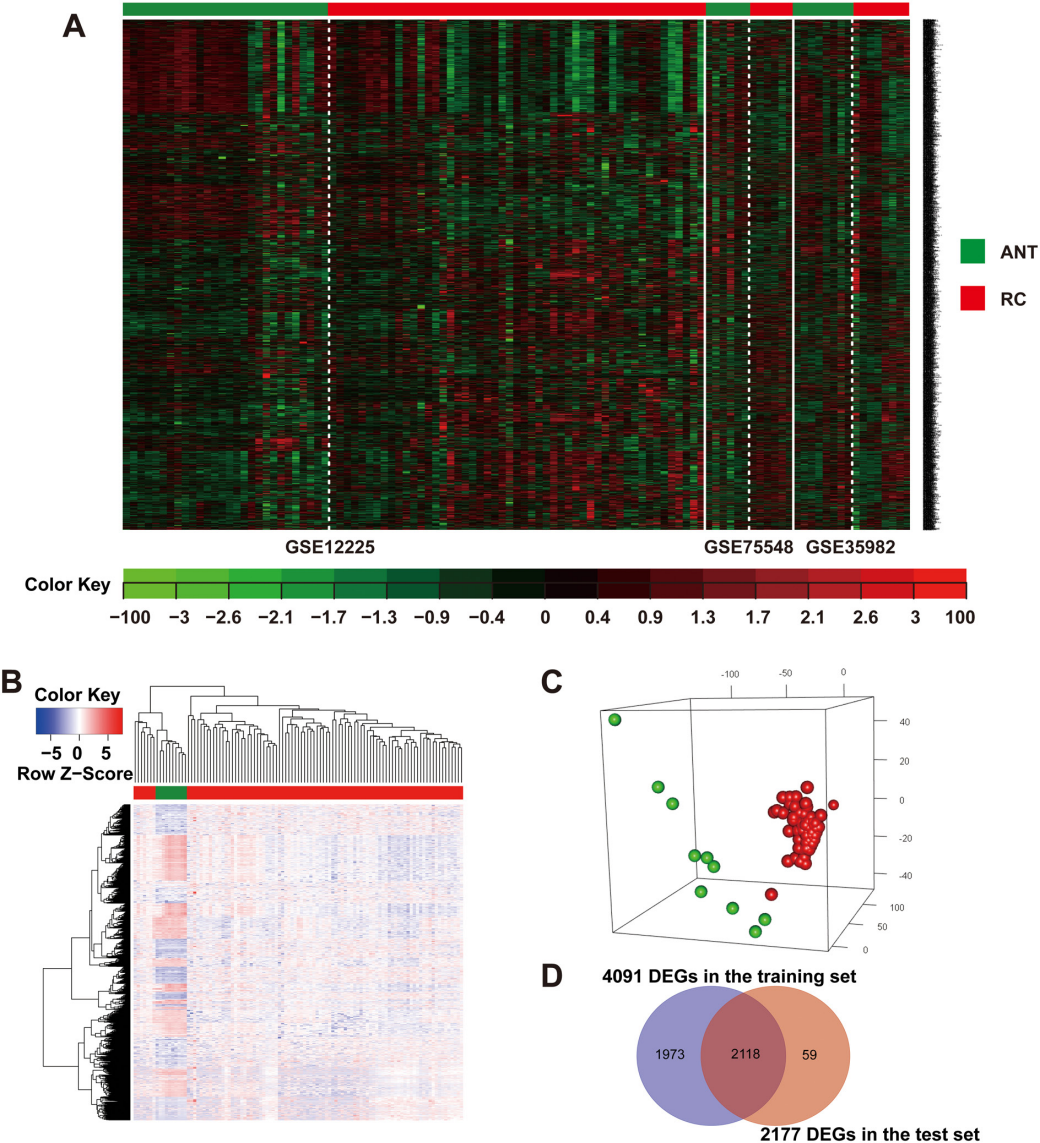
Identification of consensus DEGs in the training and test sets of rectal cancer patients **(A)** Heat map and two-way hierarchical clustering based on 4091 DEGs that were differentially expressed between RC and ANT samples of the training set. ANT (green label) and RC (red label) samples fell into separate clusters. **(B)** The 2177 DEGs RC (red label) vs. ANT (green label) of the TCGA-READ test set. Each column represents a sample, whereas each row represents the mRNA expression level. The color scale represents the raw Z-score ranging from blue (low expression) to red (high expression). Dendrograms beside each heat map correspond to the hierarchical clustering of the 2177 DEGs by the expression level. **(C)** PCA plot showing complete, unsupervised separation of the 105 array samples into 95 RC (red) and 10 ANT (green) samples. **(D)** A Venn diagram showing the overlap of DEGs detected by the training and test sets.

The training set calculated the six quantitative quality control (QC) measures by standardized mean ranks and principal component analysis (PCA) biplots within MetaQC (Table 1 and Figure 1A) [16]. GSE34472 was detected in low quality RC samples and was omitted from the training set [17]. The training set finally consisted of 65 RC samples and 42 ANTs, and it had 8153 gene symbols in common. A total of 6603 gene symbol sets passed the filtering criteria. We identified 4091 mRNAs that were consistently DEGs using the moderated *t* test, Fisher’s method by summarizing -log(p-value) across studies and running 300 permutations for the meta-analysis to infer the *P*-values (Figure 1B) [18]. As expected, hierarchical clustering of the three datasets using the remaining 4091 DEGs distinguished RC from ANT samples (Figure 2A).

**Table 1:** Quality control results of RC in the different datasets

No.	Study	IQC	EQC	CQCg	CQCp	AQCg	AQCp	Rank
1	GSE75548	3.92	2	1.8*	2.23	1.9*	1.13*	1.83
2	GSE12225	4.74	2	0.68*	1.66*	1.08*	2.38	2.00
3	GSE35982	5.62	2	0.02*	10.03	0*	0.07*	2.67
4	GSE34472	1.3*	0.4*	0.24*	0.36*	0.56*	0.11*	3.50

RC, rectal cancer; GSE, GEO dataset; IQC, internal quality control indexes; EQC, external quality control indexes; CQCg and CQCp, consistency of differential expression quality control indexes for genes and pathways; AQCg and AQCp, accuracy quality control indexes for genes and pathways.

* *P*-value not significant after Bonferroni correction.

The raw level-3 RNAseq data from 20,501 mRNAs in 95 RC samples and 10 ANT samples were downloaded from TCGA [1, 8, 10]. A total of 2177 DEGs were identified by linear models within the microarray analysis (LIMMA), among which 1092 were up-regulated and 1085 were down-regulated in RC versus ANT (Supplementary Table 2). This mRNA signature allowed for the separation of RC samples from ANT samples in the 2-way hierarchical cluster (Figure 2B) and in the PCA plot (Figure 2C). A total of 2118 overlapping DEGs were chosen by both training and test sets (Figure 2D).

### Co-expression network construction and module preservation analysis

**Figure 3:**
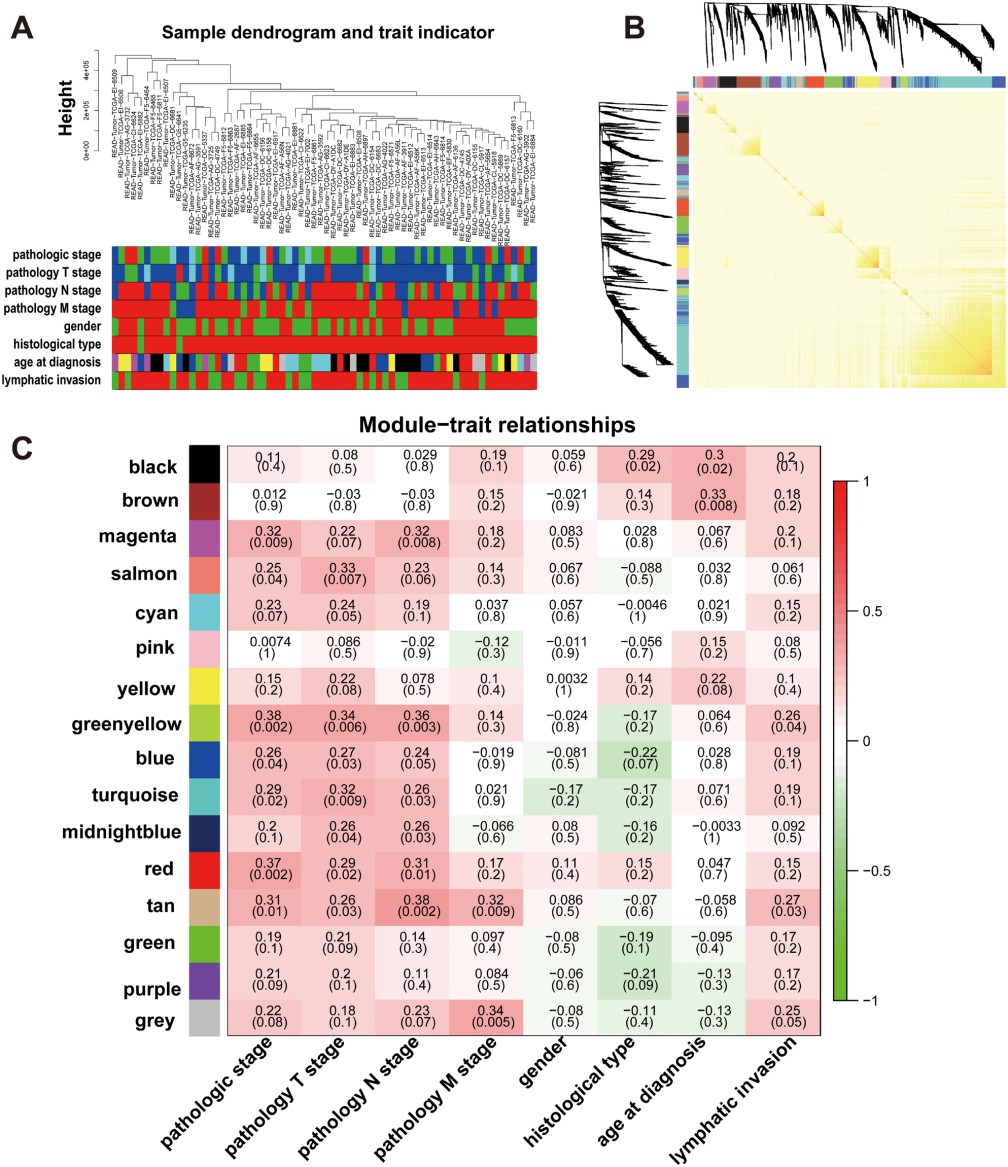
Network construction of the weighted co-expressed genes and their associations with clinical traits **(A)** Hierarchical clustering tree of the TCGA-READ samples based on the DEGs. Dendrogram tips are labeled with the TCGA-READ unique name and experiment identifier. In the hierarchical dendrogram, lower branches correspond to higher co-expression (height = Euclidean distance). Identical colors in the eight bands below the dendrogram depict the TCGA-READ clinical traits. **(B)** Heat map view of topological overlap of co-expressed genes in different modules. The heat map was generated from the topological overlap values between genes. The genes were grouped into modules labelled by a color code, which are given under the gene dendrogram on both sides. The topological overlap was high among genes of same module. **(C)** Module-trait relationships for age at diagnosis, gender, histological type, lymphatic invasion and pathologic stage. Numbers shown represent Pearson correlations between the modules and traits. *P*-values are in parentheses. Numbers on the color bar refer to the strength of the correlation in the table (red = 1, green = -1). (T: extent of the tumor; N: extent of spread to the lymph nodes; M: presence of metastasi).

We included 2118 DEGs from 70 RC patients with complete clinical traits and prognostic information of the TCGA-READ test set to construct co-expression networks via WGCNA. After four outlier samples were discarded, the connections between the genes in the gene network were in line with a scale-free network distribution when the soft threshold power β was set at 3 (Supplementary Figure 2A-2D). The dynamic tree cut method identified modules with similar expression profiles (Figure 3A, 3B). After highly similar modules were merged (Figure 3B), a total of 16 co-expressed modules were identified, ranging in size from 11 to 623 genes, whereas the “grey” module was reserved for genes that were not co-expressed (Figure 3C).

After comparing the TCGA-READ test set with the validation set (GSE29621) [19], the summary preservation statistics used in determining whether a reference network is found in another test network were visualized [20]. The green-yellow and salmon modules were well preserved, with low median Rank statistics and Z-summary statistics larger than 10 (Supplementary Figure 3) [7, 21].

### Identification of clinicopathological modules

It is important to identify modules that have the most significant associations with clinical features. We sought to explore whether any of the groups of genes from each of the identified modules were correlated with the clinical variables of RC. The list of genes in each module is presented in Supplementary Table 3. The magenta (94 genes) module yielded significant Pearson’s correlation coefficient (PCC) with the pathological stage (R = 0.32, *P* = 0.003) and the pathology N stage (R = 0.32, *P* = 0.008). The salmon (41 genes) module yielded significant PCC with the pathological stage (R = 0.25, *P* = 0.04) and pathology T stage (R = 0.33, *P* = 0.007). The red (126 genes) module yielded significant PCC with the pathological stage (R = 0.37, *P* = 0.002), the pathology T stage (R = 0.29, *P* = 0.02) and pathology N stage (R = 0.31, *P* = 0.01). The tan (44 genes) module yielded significant PCC with the pathological stage (R = 0.31, *P* = 0.01), the pathology T stage (R = 0.26, *P* = 0.03), pathology N stage (R = 0.38, *P* = 0.002), pathology M stage (R = 0.32, *P* = 0.009) and lymphatic invasion (R = 0.27, *P* = 0.03). The green-yellow (54 genes) module yielded significant PCC with the pathological stage (R = 0.38, *P* = 0.002), the pathology T stage (R = 0.34, *P* = 0.006), pathology N stage (R = 0.36, *P* = 0.006) and lymphatic invasion (R = 0.26, *P* = 0.04) (Figure 3C), which was used as the pathological stage module in subsequent analyses. These results suggested that the highly co-expressed genes in the same module have potential biological significance [6]. Each module might represent specific clinical features of RC patients [3, 22].

### Identification of overall survival modules

**Figure 4:**
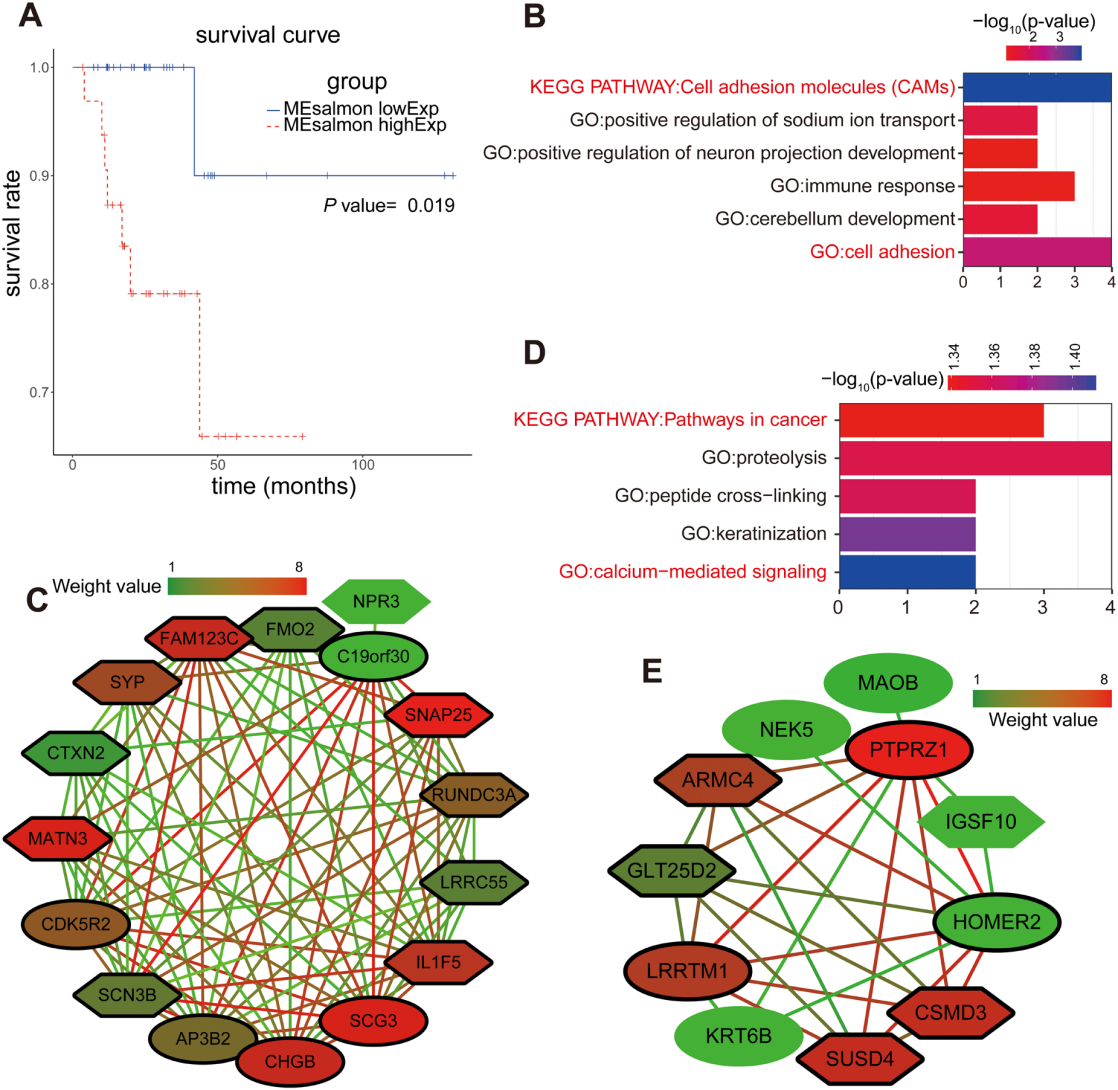
Enrichment analysis and sub-network genes of the green-yellow and salmon modules **(A)** Survival analysis based on the gene expression pattern in the salmon module. Impact of the expressed genes in the salmon module on the overall survival. **(B)** GO enrichment and KEGG analysis for the 54 module genes included in the green-yellow module. The original significance was transformed to “ – log_10_(P)” to plot the curve. **(C)** Visualization of the network connections among the most connected genes in the green-yellow module. Lines connecting two genes represent an association between the genes, and the color of the connecting line represents the weighted value of the two genes in the network. The colors of the nodes corresponded to the weighted value of the modular assignments. Elliptic and sexangular nodes indicate upregulated or downregulated sub-network genes in the modules. The nodes with a bold circle represent network hub genes identified by further analysis. **(D)** GO enrichment and KEGG analysis for the 41 module genes included in the salmon module. **(E)** Visualization of the network connections among the most connected genes in the salmon module.

We also explored the significant associations of these modules to overall survival given their biological importance. We applied the Cox regression model in 66 RC patients with complete survival data to calculate the HRs and corresponding *P*-values for each dichotomized module (Table 2). The salmon module, which was defined as the overall survival module within the sixteen merged modules, was shown to have significant associations with the prognosis of RC patients. The upregulated expression of the genes within the salmon module indicated poor outcomes of overall survival (HR = 13.534, *P* = 0.014). From the Kaplan-Meier curves, we also found that the decreased expression of genes within the salmon module indicated better outcomes of RC patients in the salmon module (*P* = 0.019) (Figure 4A).

**Table 2:** Correlation between gene co-expression modules and overall survival

	Overall survival		
	HR	CI (95% Cl)	*P*-value
ME black	0.258	0.054-1.220	0.087
ME blue	2.075	0.596-7.224	0.252
ME brown	0.448	0.115-1.747	0.248
ME cyan	1.245	0.375-4.146	0.721
ME green	0.736	0.206-2.628	0.637
ME green-yellow	2.307	0.605-8.791	0.221
ME grey	1.232	0.376-4.041	0.731
ME magenta	1.080	0.324-3.600	0.900
ME midnight-blue	1.868	0.545-6.396	0.320
ME pink	0.913	0.274-3.046	0.883
ME purple	0.234	0.049-1.106	0.067
ME red	2.802	0.742-10.579	0.129
ME salmon	***13.534***	***1.712-106.958***	***0.014***
ME tan	4.242	0.912-19.725	0.065
ME turquoise	2.487	0.658-9.400	0.180
ME yellow	1.440	0.433-4.790	0.552

The results of the survival analysis of the salmon module were consistent with their biological significance found by PCC. The salmon module was previously shown to correlate with the pathological stage (PCC = 0.25, *P* = 0.04) and the pathological T stage (PCC = 0.33, *P* = 0.007). Thus, the high expression of genes within the salmon module might represent a higher pathological stage and the T stage of RC with poor prognosis.

### Enrichment analysis and sub-network genes of green-yellow and salmon modules

We focused on the green-yellow module and carried out enrichment analysis because this module correlated strongly with the clinical features of RC patients, especially with the pathological stage. However, other modules showed weaker correlations with the phenotypic characteristics of RC. Interestingly, the green-yellow module was significantly enriched for cell adhesion for Gene Ontology (GO) and Kyoto Encyclopedia of Genes and Genomes (KEGG) (Figure 4B). We used the molecular complex detection (MCODE) algorithm (http://apps.cytoscape.org/apps/mcode) to analyze a subset of the co-expression network (threshold = 0.4). When the node density cut-off was set at 2, the node score cut-off at 0.2, the *k*-core at 2, and the maximum depth at 100, the rank 1 cluster was identified. We found that there were 15 stage sub-network genes in this cluster (*CHGB, SCG3, SYP, SNAP25, SCN3B, C19orf30, LRRC55, FMO2, CDK5R2, AP3B2, FAM123C, CTXN2, RUNDC3A, IL1F5,* and *MATN3*) (Figure 4C).

The GO and KEGG pathway enrichment analysis of the genes within the salmon module showed that cancer (*P* = 0.046) and calcium-mediated signaling (*P* = 0.039) pathways were significantly affected when the condition of *P* < 0.05 was applied (Figure 4D). We utilized the MCODE algorithm to obtain seven sub-network genes which were defined as overall survival hub genes in the rank 1 cluster (*GLT25D2, LRRTM1, ARMC4, CSMD3, SUSD4, HOMER2,* and *PTPRZ1*) (Figure 4E).

### Further screening and identification of pathological stage candidate biomarkers by ANOVA, survival and ROC curve analysis

ANOVA was carried out to determine the 15 pathological stage sub-network genes that were expressed separately and differentially. Meanwhile, the difference between localized RC (pathological stages I and II) and non-localized RC (pathological stages III and IV) by an independent *t* test for each of the 15 sub-network hub genes was determined. As a result, seven pathological stage hub genes (*SCG3, SYP, SNAP25, CDK5R2, AP3B2, FAM123C,* and *RUNDC3A*) that had a *P*-value less than 0.05 were designated to be significantly expressed (Supplementary Figure 4).

**Figure 5:**
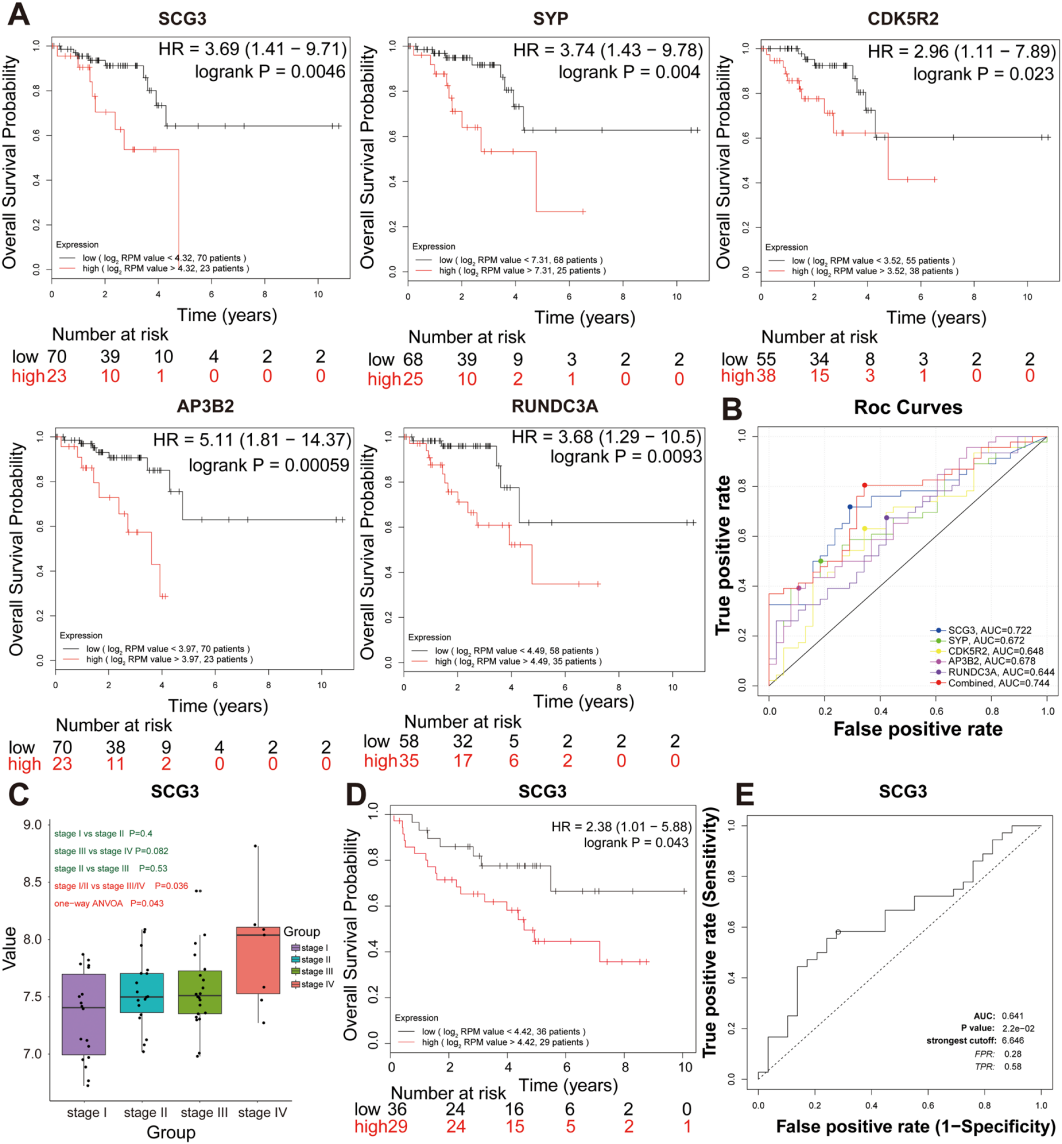
Further screening and validation of pathologic stage candidate biomarkers by survival and ROC curve analyses **(A)** Survival analysis of the five hub genes in the TCGA-READ test set. For the survival curves of the patients in different groups, solid red lines represent the high expression of hub genes and solid black lines represent the low expression of hub genes. **(B)** ROC analysis of the five hub genes in the TCGA- READ test set. Receiver operating characteristic (ROC) curve and area under the curve (AUC) statistics to evaluate the diagnostic efficiency of the hub genes in the TCGA-READ test set, which distinguished between localized and non-localized RC patients. (**C)** Boxplots of SCG3 mRNA expression across different pathologic stages in the validation set. **(D)** Survival analysis of SCG3 mRNA expression in the validation set. **(E)** ROC analysis of SCG3 mRNA expression in the independent validation set.

To further investigate whether the seven pathological stage hub genes correlated with the survival of RC patients, Kaplan-Meier analysis revealed that the five pathological stage hub genes (*SCG3, SYP, CDK5R2, AP3B2,* and *RUNDC3A*) with the highest levels significantly correlated with the markedly reduced overall survival of RC patients (Figure 5A), suggesting the important roles of five pathological stage hub genes in progress and prognosis of RC patients. Five pathological stage hub genes provided a high classification accuracy between localized RC and non-localized RC, which was estimated using receiver operating characteristic (ROC) curve analysis. AUC values for five pathological stage hub genes were greater than 0.64 in the TCGA-READ test set (Figure 5B). We could attain a best performance on accuracy by a univariate linear regression model built on a panel of the combined 5 pathological stage hub genes (AUC = 0.744): risk score = 0.369 × SCG3 + 0.296 × SYP + 0.128 × RUNDC3A -0.073 × CDK5R2 + 0.115 × AP3B2 -3.55 (Figure 5B).

Thus, five pathological stage hub genes were selected as the candidate genes for further validation. ANOVA, survival and ROC curve analysis were conducted to validate the five pathological stage hub genes in the validation set (GSE29621). Only SCG3 with a *P*-value less than 0.05 were declared to be significantly expressed in four pathological stages of RC (ANOVA test, *P* = 0.043) (Figure 5C). In addition, we carried out a single-gene survival analysis in order to reveal which genes were most significantly associated with overall survival. Unfortunately, we only found an increased expression of the SCG3 (HR 2.381 [95 % CI 1.01 – 5.88], *P* = 0.043) with poor prognosis (Figure 5D). SCG3, which was chosen as the pathological stage candidate biomarker, provided a high classification accuracy between localized RC and non-localized RC, which was estimated using ROC curve analysis (Figure 5E).

### Further screen and validation of novel overall survival candidate biomarkers

To further narrow the overall survival candidate biomarkers that harbor great significance among the declared seven overall survival hub genes, we chose to use the survival and ROC curve analysis to summarize the expression patterns of the hub genes.

**Figure 6:**
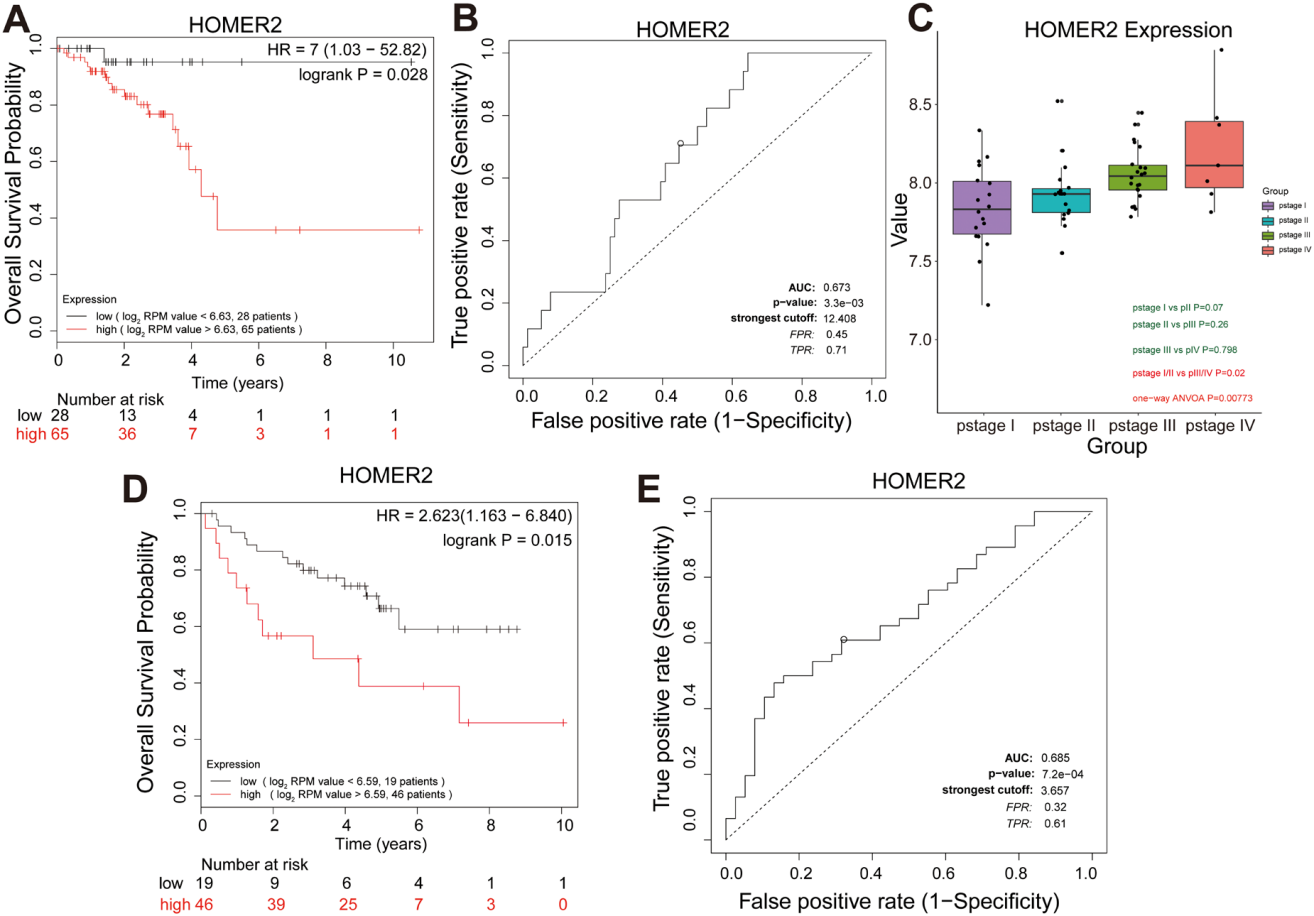
Further screening and validation of novel overall survival candidate biomarkers by survival and ROC curve analyses **(A)** Survival analysis of HOMER2 mRNA expression in the TCGA-READ test set. For survival curves of patients in different groups, solid red lines represent high expression and solid black lines represent low expression. **(B)** ROC analysis of HOMER2 mRNA expression in the TCGA-READ test set. Receiver operating characteristic (ROC) curve and area under the curve (AUC) statistics to evaluate the diagnostic efficiency of HOMER2 mRNA expression in the TCGA-READ test set, which distinguished between high risk and low risk RC patients. **(C)** Boxplots of HOMER2 mRNA expression across different pathologic stages in the validation set. **(D)** Survival analysis of HOMER2 mRNA expression in the validation set. **(E)** ROC analysis of HOMER2 mRNA expression in the independent validation set to distinguish between high risk and low risk RC patients.

Survival analysis was performed for each overall survival hub gene. The patients were dichotomized into two equal groups by the expression level of the overall survival hub genes. Survival analysis for the seven overall survival hub genes in the salmon module was conducted for 93 RC patients, and we found that only HOMER2 significantly associated with patient overall survival in the TCGA-READ set (Supplementary Figure 5). Kaplan-Meier analysis revealed that the high HOMER2 level significantly correlated with the markedly reduced overall survival of RC patients (HR 7 [95% CI 1.03 − 52.82], *P* = 0.028) (Figure 6A), suggesting the important roles of HOMER2 in the prognosis of RC patients. The sensitivity and specificity of the HOMER2 expression level on the survival outcome was assessed by ROC curve analysis, and the area under the ROC curve was used to evaluate survival prediction efficiency of HOMER2. The AUC value of HOMER2 was 0.673, which was close to 0.7 (Figure 6B). Thus, HOMER2 was chosen as the overall survival candidate biomarker for further validation to determine whether there was a significant correlation between HOMER2 and the prognosis of RC patients. ANOVA, survival and ROC curve analysis were carried out to confirm HOMER2 in the validation set (GSE29621). Only HOMER2 had a *P*-value less than 0.05 and was determined to be significantly differentially expressed in the four pathological stages of RC (ANOVA test, *P* = 0.008) (Figure 6C). In addition, we carried out a single-gene survival analysis to reveal which genes were most significantly associated with overall survival. Unfortunately, we only found the increased expression of HOMER2 (HR 2.632 [95 % CI 1.163 – 6.840], *P* = 0.015) to associate with poor prognosis (Figure 6D).

The sensitivity and specificity of the expression level of HOMER2 on the survival outcome were assessed by ROC curve analysis, and AUC was used to evaluate survival prediction efficiency of HOMER2. The AUC value of HOMER2 was 0.685 (Figure 6E).

## DISCUSSION

Gene signatures identified from genome-based assays are known to contribute to RC stratification [23]. Numerous studies have defined in part the gene signatures predicting the survival outcome or recurrence of RC [24–34]. In this study, six mRNA datasets were subjected to meta-analysis and WGCNA to identify hub genes associating with clinical characteristics as well as RC progression and prognosis [4, 5, 35].

We identified sixteen distinct modules from 2118 consensus genes that passed the meta-analysis filtering criteria and TCGA-READ validation for WGCNA [36]. Among the identified modules, the magenta, red, tan, and green-yellow modules associated with the pathological stage, especially the green-yellow module which had the strongest correlation with the pathological stage [12, 37, 38]. In addition, the brown module correlated with the age of diagnosis. Correlations were also found between the red, tan, and green-yellow modules and pathological stages T and N, suggesting that the highly co-expressed genes within the same module were of similar biological significance.

Given that the biological significance of these modules might be related to the potential clinical manifestations, we performed Cox regression analysis for each module to determine their survival outcomes [1, 11, 39, 40]. Poor outcomes were found for the high expression group of the salmon module, which was consistent with previous results indicating that the high expression of the salmon module might represent a higher pathological stage and the T stage [2].

The green-yellow and salmon modules were found to correlate with the pathological stage and overall survival, respectively. Thus, we selected the green-yellow module for the subsequent analysis because this module is likely to represent tumor staging characteristics more accurately [41]. Cell adhesion pathways suggested by GO and KEGG were over-represented in the green-yellow module. To some extent, these results also partially explained the increasing stages of RC patients. By means of one-way ANOVA and an independent sample *t* test, seven pathological stage hub genes *(SCG3, SYP, SNAP25, CDK5R2, AP3B2, FAM123C,* and *RUNDC3A)* could effectively distinguish non-localized RC from localized RC. Furthermore, the expression levels of five pathological stage hub genes *(SCG3, SYP, CDK5R2, AP3B2, RUNDC3A)* significantly associated with both the pathological stage and overall survival, but only *SCG3* mRNA expression was replicated in the validation set. The diagnosis model of the combined five pathological stage hub genes, which provided a high classification accuracy, might be good biomarkers for distinguishing between localized and non-localized RC (Figure 5B). SCG3, a member of the multifunctional granin family, played a key role in secretory granule biogenesis, which involves the cellular uptake of endogenous and exogenous toxins [42]. SCG3 was identified as the most sensitive and specific marker for circulating tumor cells in small cell lung cancer and was indicative of a worse survival outcome. As is well known, response to standard chemotherapy is important in determining survival. SCG3 was also evident in patients with poor response to standard chemotherapy [24]. There is potential mechanistic association between SCG3 expression in tumors and response to platinum-based therapy or topoisomerase II inhibitors [24]. As RE-1 silencing transcription factor is a transcriptional repressor in cancer, higher expression of SCG3 mRNA may increase the aggressive potential of the tumor or reduce the drug sensitivity of RE-1 silencing transcription factor depleted tumors. For the first time, we show SCG3 as a biomarker of RC pathological stage and prognosis.

Further analysis identified the overall survival candidate biomarkers from the salmon module, thus demonstrating the significant association with survival in the test and validation sets. Among the seven hub genes related to overall survival, increased HOMER2 expression associated with the increased pathological stage and poor survival of RC patients. Moreover, such expression may be useful in evaluating the survival risk because the AUC, which was used to evaluate the survival prediction efficiency of HOMER2, was 0.673 and 0.685 in the test and validation set, respectively. HOMER2 gene encodes a member of the homer family of dendritic proteins and regulates group 1 metabotrophic glutamate receptor function. HOMER2 is also a promising biomarker for cancer prognosis. HOMER2 is known to associate with overall survival and disease-free survival in early stage non-small cell lung cancer [43]. HOMER2 which was identified as a binding partner of MYO18B, interacted with the C-terminal region of MYO18B, a candidate tumor suppressor gene involved in the pathogenesis of human cancers including colorectal cancer [44]. Additionally, methylation of HOMER2 was reported to be a valuable biomarker which significantly discriminated CRC patients from controls [45].

**Figure 7:**
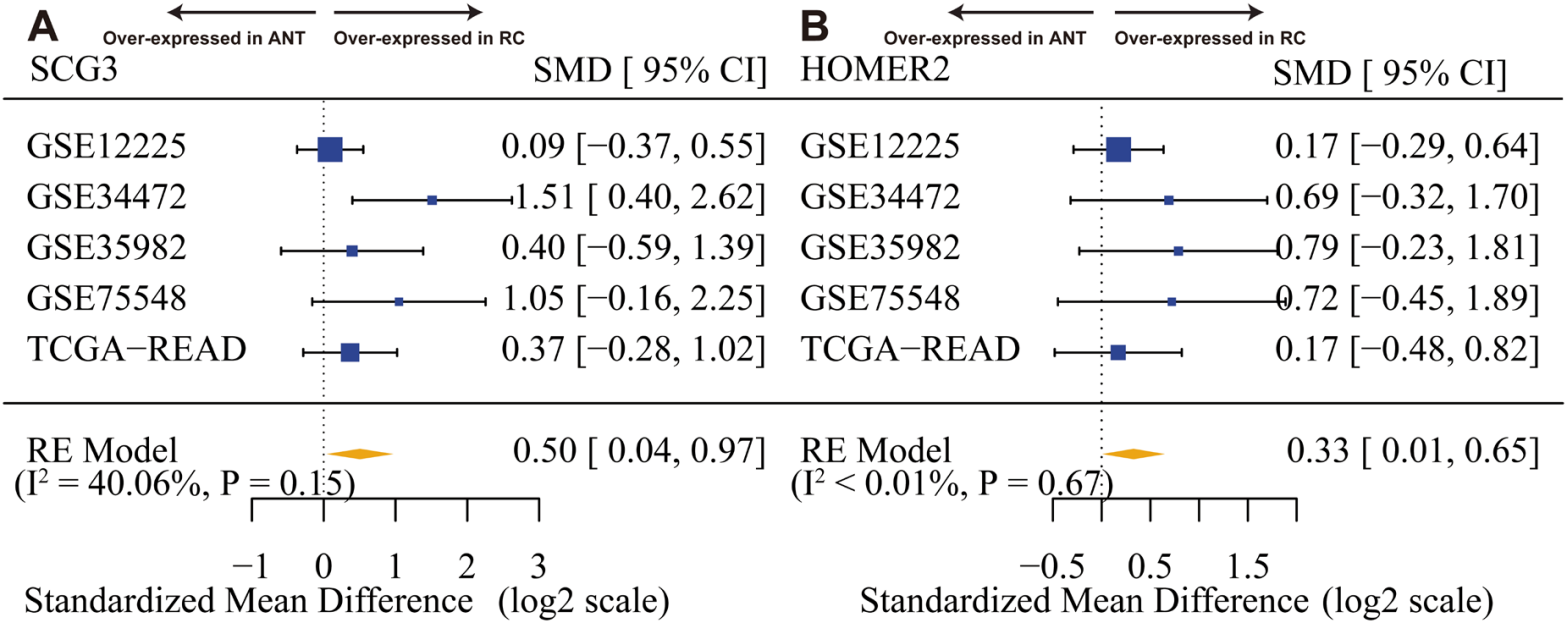
SCG3 and HOMER2 are overexpressed in rectal cancer **(A)** Forest plot of SCG3 expression across all training and test pooled analysis datasets. **(B)** Forest plot of HOMER2 expression across all training and test pooled analysis datasets. The x-axis is the standardized mean difference between rectal cancer (RC) and adjacent normal tissue (ANT) on a log2 scale. Thus, a value of 1 signifies a 2-fold difference in gene expression between cancer and normal.

In addition, we tentatively estimated between-studies heterogeneity in effect sizes of twelve hub genes (Supplementary Figure 6), especially SCG3 and HOMER2 (Figure 7A, 7B). Only one gene (SUSD4) had significant between-studies heterogeneity (I^2^ = 67.32%) and was ejected because we did not have sufficient information available to explore heterogeneity (Supplementary Table 1).

To our knowledge, this is the first study investigating the relationship between the co-expression network of clinical traits and genes in patients with RC [46]. In summary, we identified sixteen gene co-expression modules from five RC datasets using meta-analysis and WGCNA [47]. We associated a number of these network modules to RC clinicopathological variables, as well as to overall survival, and uncovered the gene expression signature associated with RC pathology stage and overall survival. We identified several significant pathways, as well as five potential pathological stage hub genes and seven overall survival hub genes using MCODE. Utilizing the TCGA-READ dataset, we constructed a diagnosis model of five mRNA signatures, and the validation cohort confirmed that the panel may be a useful biomarker for prognosis in RC. Of greatest interest, we found that the increased SCG3 and HOMER2 expression associated with the increased pathological stage and poor survival in the test and validation set among RC patients, providing two useful markers of RC and suggesting that it may help to identify those with more aggressive disease. Nevertheless, multicenter randomized controlled studies and *in vivo* and *in vitro* experiments are still required to evaluate the possible application of the molecular signatures for survival prediction and to characterize the hub genes functionally for the application of this approach in specific clinical settings.

## MATERIALS AND METHODS

### Data collection, preprocessing, and normalization

A public microarray repository was curated to search through PubMed, Gene Expression Omnibus (GEO) (accession numbers GSE75548, GSE34472, GSE35982, GSE12225, and GSE29621), ArrayExpress (accession number E-GEOD-34472), and TCGA-READ datasets up to September 2016. Only initial experimental studies that screened different mRNAs from RC and adjacent normal tissue samples in humans were included. After writing off the duplicated datasets, the combined datasets (GSE75548, GSE34472, GSE35982, GSE12225, and TCGA-READ) containing 168 RC and 60 normal rectal tissue samples were generated (Supplementary Table 1). The raw datasets were preprocessed individually using the LIMMA software package with log_2_ transformation and annotated by converting different probe IDs to the respective gene symbols. Duplicate gene expression values were averaged.

### Integrated analysis of the gene expression datasets of the training set

To make the five microarray datasets derived from the five different platforms compatible for data analysis, we used the MetaOmics software package (http://www.pitt.edu/∼tsengweb/MetaOmicsHome.htm) to integrate and analyze the GEO datasets [48]. Firstly, the MetaQC software package, which provided a quantitative and objective quality control tool for determining the inclusion/exclusion criteria for the meta-analysis, eliminated the GSE34472 dataset [48, 49]. Thus, the training set included three GEO datasets (GSE75548, GSE35982, and GSE12225) after Quality Control. Secondly, to distinguish the DEGs between RC and ANT, the MetaDE software package limited the mean and standard deviation (SD) filter thresholds, which were set at 10% to filter minor changes in gene expression levels [50]. Considering the different stringencies of the various methods, Fisher’s method was favored for the meta-analysis (Figure 1B). For Fisher’s method, the modified *t* test and the permutation method (nPermutations = 300) were used to extrapolate the *P*-values [51]. *P*-values less than 0.05 were considered statistically significant for the DEGs. The heatmaps illustrating the DEG patterns were also generated [50].

### Integrated-signature gene analysis of TCGA-READ as the test set and consensus screening of the DEGs

To further limit analyses to genes common to all datasets, the results of the DEG training set were validated in the TCGA-READ dataset, which was considered as the test set. The TCGA-READ mRNA and clinical data (level 3) of the corresponding patients (RC and ANT) were downloaded from the TCGA data portal (up to May 20, 2016). The TCGA-READ DEGs were analyzed using an empirical Bayes approach within the LIMMA software package. The DEGs of the test set with a |log_2_ fold change (FC)| ≥ 0.5 and an adjusted *P*-value less than 0.05 were selected for subsequent analysis. A total of 2177 gene symbols of the test set passed the filtering criteria. We then created an overlapping gene set by selecting common official gene symbols in both training and test sets, resulting in a total of 2118 intersecting genes. The consensus DEGs, which ensured that RC and ANT samples were well characterized, were chosen for WGCNA [10, 37, 52].

### Preprocessing of clinical information

Clinical information obtained from the TCGA-READ dataset (American Joint Committee on Cancer pathological TNM stage, gender, age at initial pathological diagnosis and histological type (mucinous adenocarcinoma or adenocarcinoma), especially vital status and time to last follow-up) for 70 patients was used after eliminating incomplete clinical traits and gene expression.

### Weighted gene co-expression network construction and module preservation analysis

The TCGA-READ dataset with intact clinical features and prognostic information was selected for constructing the scale-free gene co-expression networks within the WGCNA software package [8]. Firstly, the appropriate soft threshold power was automatically estimated and generated as described for the standard scale-free networks. In this case, the power of β, which was set at 3 (scale-free R^2^ = 0.89), was auto-selected. Moreover, the weighted adjacency matrix was constructed using the power function ADJ_mn_ = |COR_mn_|^β^ (COR_mn_ = Pearson’s correlation between gene m and gene n; ADJ_mn_ = adjacency between gene m and gene n). β was the soft thresholding parameter, which was used to transform adjacencies and correlations into a Topological Overlap Matrix (TOM), and then the corresponding dissimilarity (1-TOM) was calculated. Finally, module identification was carried out with the dynamic tree cut method by hierarchically clustering the genes using 1-TOM as the distance measure with a deep split value of 2 and a minimum size cut-off of 30 for the resulting dendrogram. Highly similar modules were marked by clustering and merged with a height cut-off of 0.25. Module preservation and quality statistics were computed using the module Preservation function (nPermutations = 200) within the WGCNA software package between the TCGA-READ test set and the GSE29621 validation set (Supplementary Table 1) [20].

### Identification of clinical feature modules, survival analysis and efficacy evaluation of pathological stage hub genes

Module eigengenes (MEs), which are the first principal components in the PCA for each gene module, summarized the expression patterns of all genes into a single characteristic expression profile within a given module. The dynamic decision-making tree, node splitting method and cluster analysis of the squared Euclidean distance were used to identify MEs related to these clinical features, especially those involved in the progression and prognosis of RC. Spearman’s correlation analysis was carried out to confirm the object module, which was the most relevant module between the MEs and clinical traits. Depending on these, the module that had the highest Spearman’s correlation coefficient for the pathological stage and MEs in the object module was defined as the pathological stage module. Hub genes that had been selected in the pathological stage module were obtained using the MCODE algorithm plugin within the Cytoscape software package (version 3.4.0) [53].

### Survival analysis of individual modules and efficacy evaluation for survival hub genes

For the single module survival analysis, the TCGA-READ test data was dichotomized around the median expression of each gene module. The overall survival was used as the survival endpoint, which was determined via the “survival” R software package. Cox regression analysis was performed to evaluate the hazard ratio (HR). Survival curves of the significant objected module, which was defined as the survival module, were constructed by the Kaplan-Meier method and compared by the log-rank test. Survival hub genes were obtained by MCODE. Survival curves and ROC analysis were designed to identify candidate markers. The results of the survival curves and ROC analysis were next verified on an independent validation set (GSE29621) using the survival hub genes as the candidate marker input to predict the classes of prognosis in RC.

### Functional annotation and network visualization within pathological stage and survival modules

The pathological stage and survival modules were functionally annotated based on the analytical results of their gene compositions. GO functional-related to specific Biological Processes and KEGG pathway enrichment analyses were performed for the object module by using the Database for Annotation, Visualization, and Integrated Discovery (DAVID) [54]. *P*-values less than 0.05 were used as the cut-off value. Network visualization of the stage and survival modules were carried out within the Cytoscape software package. Moreover, there were strong correlations between the co-expressed module genes and their functions, and MCODE was utilized to provide deeper insights for hub genes.

### Statistical analysis

Significance differences between stage groups were determined by analysis of variance (ANOVA) or the Student’s *t* test. *P*-values less than 0.05 were considered statistically significant. The preliminary relationships between the hub genes of stage modules were demonstrated by boxplot graphs. Survival curves were constructed by the Kaplan-Meier method and compared by the log-rank test for the expression level with the stage and survival hub genes for calculating the overall survival. All Cox regression models were tested based on Schoenfeld residuals to evaluate the hazard ratio (HR) for each module. Statistical analysis was performed using the R software package (version 3.2.3). ROC analysis was used to evaluate the diagnostic value on the outcome for the expression level of each stage and survival hub genes in RC via the code of Mihaly (Supplementary File 2) [55]. Using univariate linear logistic regression of the hub genes, a classification model of the combined hub genes was built to evaluate the discriminatory capacity of localized RC and non-localized RC. Finally, the results of the survival curves and ROC analysis were validated on an independent validation set (GSE29621) using the pathological stage and survival hub genes as candidate biomarker input to predict the classes of pathological stage as well as survival of neoplastic progression and prognosis in RC patients. Because of the heterogeneity of hub genes within and among samples and datasets, the between-study heterogeneity was examined by the Cochran’s Q-test and I^2^ statistic, with *P* values for heterogeneity by the I^2^ value > 50% indicating substantial heterogeneity [56]. We used 300 Monte Carlo permutation tests after combining *P* values (Supplementary Figure 1) [57], and a DerSimonian and Laird random effects model for meta-analysis by combing effect size to select hub genes in RC.

## SUPPLEMENTARY MATERIALS FIGURES AND TABLES







## Abbreviations

The abbreviations used are:

RC: rectal cancer
ANT: adjacent normal tissue
WGCNA: weighted gene co-expression network analysis
TCGA: The Cancer Genome Atlas
READ: rectal adenocarcinoma
DEGs: differentially expressed genes
HR: hazard ratio
AUC: area under the curve
GEO: Gene Expression Omnibus
GSE: gene expression data series
LIMMA: linear models for microarray analysis
QC: quality control
SD: standard deviation
TOM: Topological Overlap Matrix
ME: Module eigengene
MCODE: molecular complex detection
GO: Gene Ontology
KEGG: Kyoto Encyclopedia of Genes and Genomes
ANOVA: analysis of variance
AJCC: American Joint Committee on Cancer
PCC: Pearson’s correlation coefficient
PCA: principal component analysis
ROC: receiver operating characteristic
DAVID: the Database for Annotation, Visualization, and Integrated Discovery
