# Supplementary material for: Identification of two novel biomarkers of rectal carcinoma progression and prognosis via co-expression network analysis
Source: Oncotarget. 2017 Jun 27;8(41):69594–609. doi: 10.18632/oncotarget.18646 (PMC5642502; doi:10.18632/oncotarget.18646)
Supplement: Supplementary file 1 [file oncotarget-08-69594-s001.pdf]

Identification of two novel biomarkers of rectal carcinoma progression and prognosis via co-expression network analysis

SUPPLEMENTARY MATERIALS

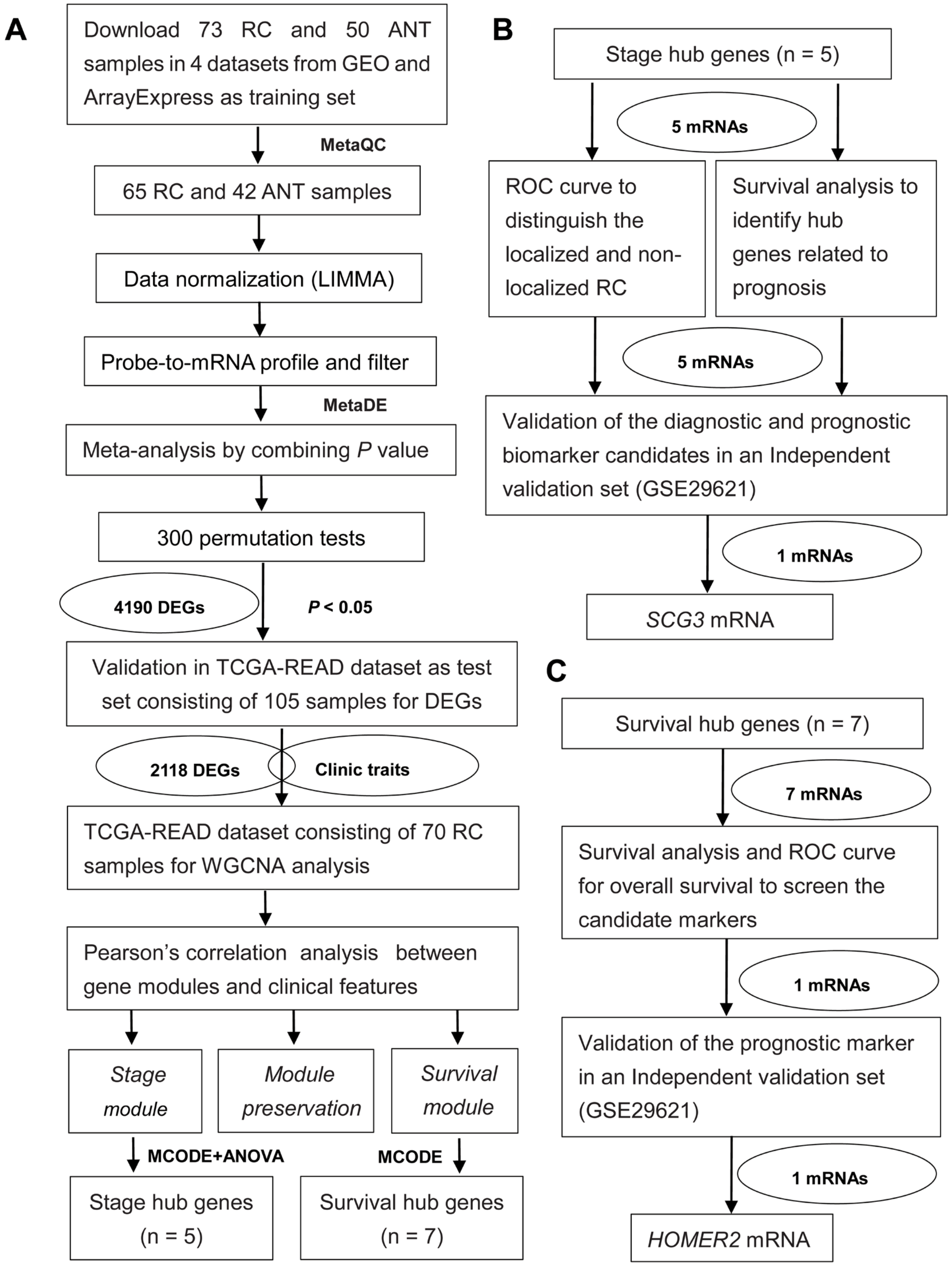

Supplementary Figure 1: A scheme of the meta-analysis workflow and WGCNA pipeline of the RC datasets. (A) A scheme of the meta-analysis and WGCNA workflow. **(B)** A flow diagram for screening the pathologic stage candidate biomarkers. **(C)** A flow diagram for screening the overall survival candidate biomarkers.

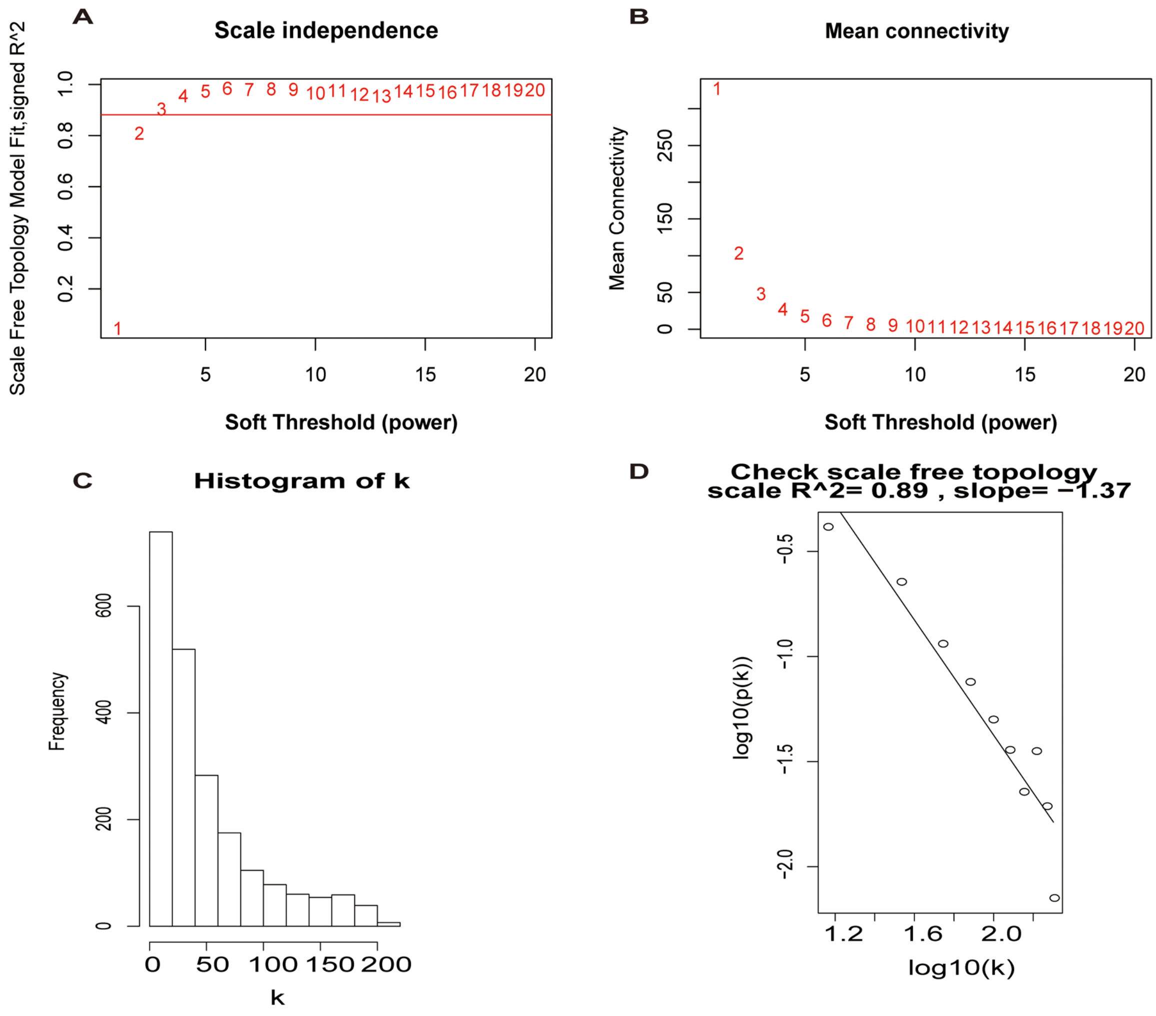

Supplementary Figure 2: Determination of parameter β of the adjacency function in the weighted gene correlation network analysis (WGCNA) algorithm. The adjacency function was weighted by the power of the correlation data between different genes; i.e., $a_{mn}= (S_{mn}, \beta)= |S_{mn}|^{\beta}$. The weighted parameter β in the formula was determined by the scale-free network law, which means that the probability (p) that a node is connected with k other nodes (p(k)) satisfies the criterion that the co-efficiency of log(k) and log(p(k)) is at least 0.8. To ensure that the average connectivity of the network was smooth, we chose β = 3 based on the diagnostic chart. **(A)** Analysis of the scale-free fit index for various soft thresholding powers β. **(B)** Analysis of the mean connectivity for various soft thresholding powers. **(C)** Histogram of connectivity distribution when β = 3. **(D)** Checking the scale free topology when β = 3.

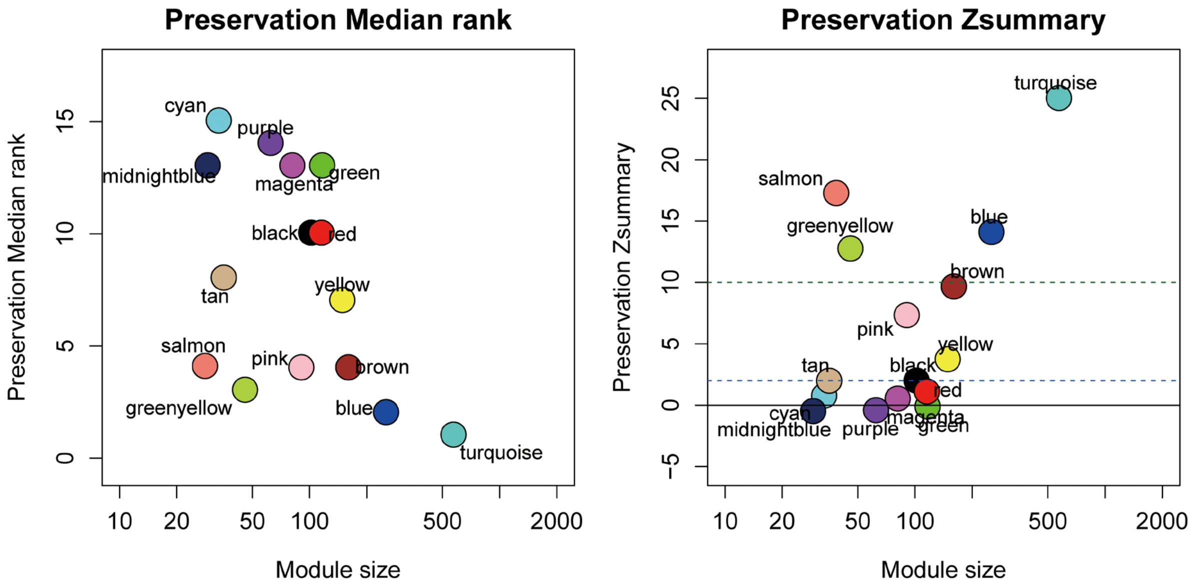

Supplementary Figure 3: The median rank and Z-summary statistics of the module preservation of the DEG modules.

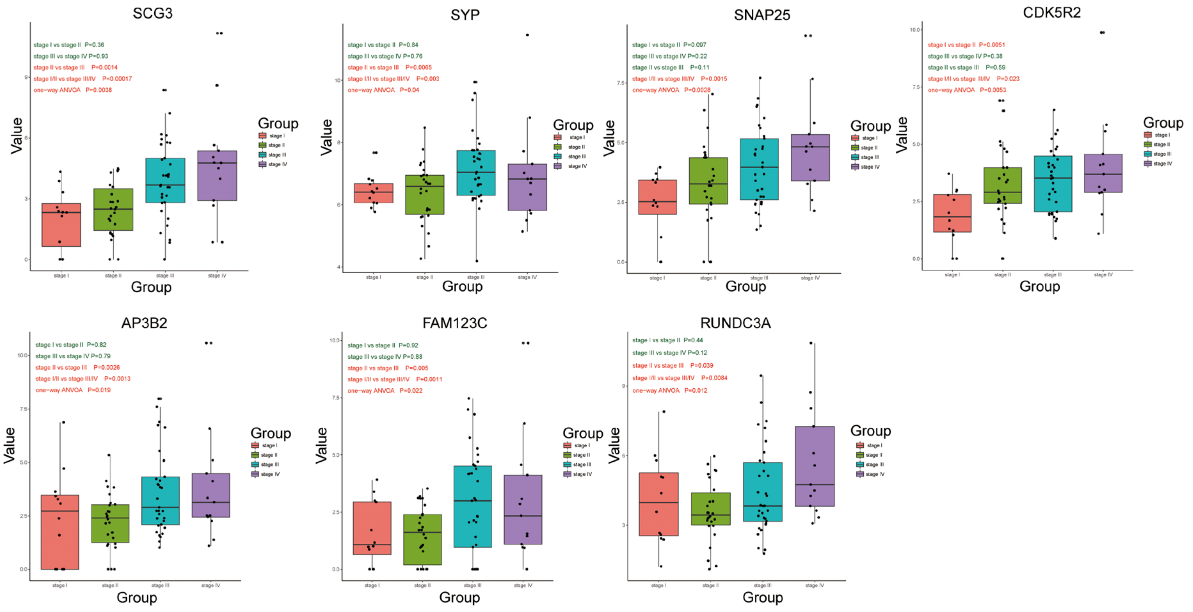

Supplementary Figure 4: Boxplots of seven hub genes across different pathological stages of the green-yellow module of the TCGA-READ test dataset.

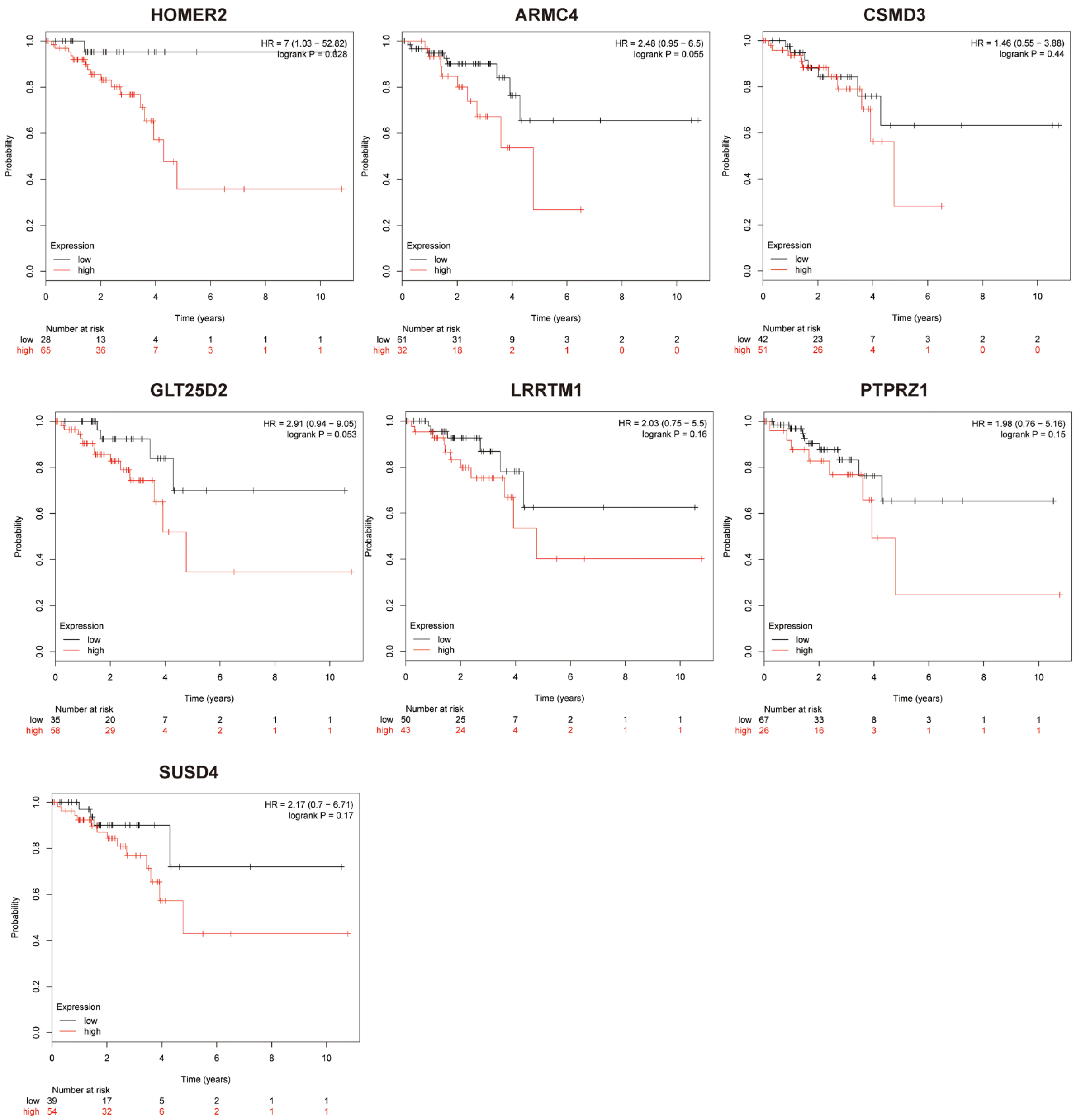

Supplementary Figure 5: Survival analyses of seven overall survival hub genes of the salmon module of the TCGA-READ test dataset. Survival curves for patients in different groups. The solid red lines represent the high expression of hub genes, and the solid black lines represent the low expression of sub-network genes.

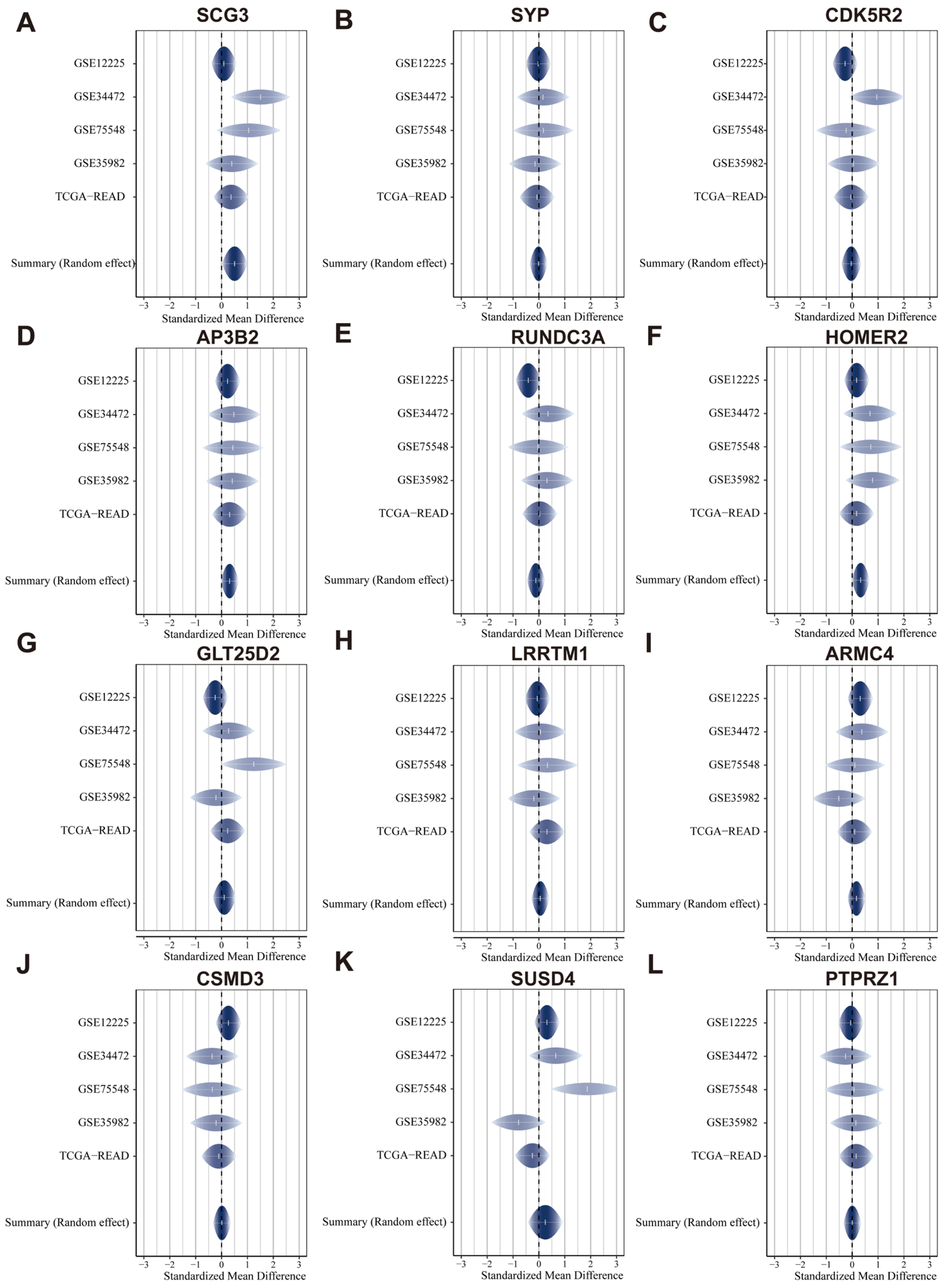

Supplementary Figure 6: Rainforest plots summarizing the expression of the five pathological stage and seven overall survival hub genes from meta-analysis. Each row represents a study with standardized mean difference between rectal cancer and normal and the confidence interval of 95%. The color of the blue raindrop is proportional to the relative effect size of each study. The dotted vertical line at 0.0 represents the null hypothesis. Thus, positive values represent overexpression in rectal cancer.

Supplementary Table 1: Characteristics of the public microarray datasets used in this study

Study	Species/array platform	Samples	Number of samples	Set
GSE75548	Illumina Human HT-12 V4.0 Expression Beadchip (GPL10558)	RC vs. control tissue	RC = 6, control tissue = 6	Training set
GSE34472/E-GEOD-34472	Agilent-014950 Human Genome CGH Microarray 4x44K (GPL8841)	RC vs. control tissue	RC = 8, control tissue = 8	Training set
GSE35982	Agilent-014850 Whole Human Genome Microarray 4x44K G4112F (GPL4133)	RC vs. control tissue	RC = 8, control tissue = 8	Training set
GSE12225	Affymetrix Human Mapping 10K 2.0 Array (GPL2641)	RC vs. control tissue	RC = 51, control tissue = 28	Training set
TCGA-READ	Human Illumina HiSeq 2000	RC vs. control tissue	RC = 95, control tissue =10	Test set
GSE29621	Affymetrix Human Genome U133 Plus 2.0 Array (GPL570)	RC	Pathological stage I: 7<br>Pathological stage II: 22<br>Pathological stage III: 18<br>Pathological stage IV: 18	Validation set

E-GEOD, ArrayExpress dataset; GSE, GEO dataset; RC, rectal cancer; TCGA, The Cancer Genome Atlas; READ, rectum adenocarcinoma.

Supplementary Table 2: Gene-level results of differentially expressed mRNAs between RC and ANT of the TCGA-READ dataset

See Supplementary File 1

Supplementary Table 3: Official gene names and Entrez gene IDs of sixteen modules listed in the gene-module

See Supplementary File 1

Supplementary Data File 1: ROC analysis script

See Supplementary File 2
