# Supplementary material for: Identification of two novel biomarkers of rectal carcinoma progression and prognosis via co-expression network analysis
Source: Oncotarget. 2017 Jun 27;8(41):69594–609. doi: 10.18632/oncotarget.18646 (PMC5642502; doi:10.18632/oncotarget.18646)
Supplement: Supplementary file 2 [file oncotarget-08-69594-s002.docx]

**Supplementary Table 2: Gene-level results of differentially expressed mRNAs between RC and ANT of the TCGA-READ dataset**

| **Official gene ID** | **Entrez ID** | **logFC** | **logCPM** | **PValue** | **FDR** | **Classification** |
| --- | --- | --- | --- | --- | --- | --- |
| KCNIP4 | 80333 | -3.8384 | 1.00794 | 6.63E-94 | 1.20E-89 | down-regulated |
| CMTM5 | 116173 | -5.7717 | -2.1622 | 9.71E-60 | 8.79E-56 | down-regulated |
| RYR3 | 6263 | -3.6532 | -0.0062 | 8.36E-59 | 5.04E-55 | down-regulated |
| GRIK3 | 2899 | -4.3901 | 1.13907 | 1.04E-56 | 4.70E-53 | down-regulated |
| BEST4 | 266675 | -5.6371 | 2.88479 | 4.13E-53 | 1.49E-49 | down-regulated |
| DPP6 | 1804 | -6.3484 | 1.25039 | 9.97E-53 | 3.01E-49 | down-regulated |
| CLEC3B | 7123 | -4.0013 | 3.92781 | 8.04E-52 | 2.08E-48 | down-regulated |
| GPM6B | 2824 | -3.819 | 2.31472 | 8.41E-51 | 1.90E-47 | down-regulated |
| CCDC69 | 26112 | -3.2813 | 5.26389 | 2.00E-48 | 4.01E-45 | down-regulated |
| FRMPD4 | 9758 | -5.9455 | -2.0276 | 4.66E-47 | 8.42E-44 | down-regulated |
| HRNBP3 | 146713 | -5.388 | 0.99134 | 6.55E-47 | 1.08E-43 | down-regulated |
| PRIMA1 | 145270 | -5.2671 | 1.82035 | 5.37E-46 | 8.10E-43 | down-regulated |
| TACR2 | 6865 | -4.9379 | 3.19438 | 2.19E-45 | 3.05E-42 | down-regulated |
| MPZ | 4359 | -3.5849 | 1.23427 | 3.35E-45 | 4.33E-42 | down-regulated |
| PLP1 | 5354 | -6.3536 | 2.63763 | 5.30E-45 | 6.39E-42 | down-regulated |
| LGI1 | 9211 | -5.7491 | -1.5546 | 8.68E-45 | 9.82E-42 | down-regulated |
| FGFBP2 | 83888 | -4.2367 | -1.1382 | 9.92E-44 | 1.06E-40 | down-regulated |
| GLP2R | 9340 | -4.6448 | -0.5401 | 1.88E-43 | 1.89E-40 | down-regulated |
| NBLA00301 | 79804 | -5.8116 | 2.47022 | 5.35E-43 | 5.10E-40 | down-regulated |
| SST | 6750 | -6.6177 | 2.13182 | 1.06E-42 | 9.56E-40 | down-regulated |
| NRXN1 | 9378 | -5.8943 | 1.08554 | 1.23E-42 | 1.06E-39 | down-regulated |
| MYOT | 9499 | -4.9361 | -1.0097 | 1.01E-41 | 8.28E-39 | down-regulated |
| FUT9 | 10690 | -6.3318 | -2.7473 | 1.52E-41 | 1.20E-38 | down-regulated |
| SCN7A | 6332 | -6.1379 | 2.57329 | 2.42E-41 | 1.82E-38 | down-regulated |
| CDH3 | 1001 | 5.0905 | 6.40795 | 2.85E-41 | 2.01E-38 | up-regulated |
| ADCYAP1R1 | 117 | -6.0564 | -2.424 | 2.89E-41 | 2.01E-38 | down-regulated |
| RSPO2 | 340419 | -5.4272 | 1.67303 | 4.58E-41 | 3.07E-38 | down-regulated |
| NPTX1 | 4884 | -5.55 | 2.90612 | 5.43E-41 | 3.51E-38 | down-regulated |
| FIGF | 2277 | -4.9491 | -0.2111 | 1.60E-40 | 1.00E-37 | down-regulated |
| EPM2A | 7957 | -2.3449 | 2.49134 | 4.53E-40 | 2.74E-37 | down-regulated |
| GRIK1 | 2897 | -4.2531 | -2.8851 | 8.44E-40 | 4.92E-37 | down-regulated |
| KY | 339855 | -4.0526 | -0.8037 | 1.08E-39 | 6.12E-37 | down-regulated |
| CADM2 | 253559 | -6.1691 | -1.2205 | 1.81E-39 | 9.92E-37 | down-regulated |
| DHRS7C | 201140 | -6.0255 | -2.9488 | 6.44E-39 | 3.43E-36 | down-regulated |
| PRKG2 | 5593 | -3.7656 | 0.195 | 1.49E-38 | 7.68E-36 | down-regulated |
| POPDC2 | 64091 | -4.0253 | 2.46297 | 1.65E-38 | 8.32E-36 | down-regulated |
| CSRP1 | 1465 | -2.5031 | 8.59744 | 1.01E-37 | 4.92E-35 | down-regulated |
| PCSK2 | 5126 | -5.9612 | -0.05 | 1.84E-37 | 8.75E-35 | down-regulated |
| CA14 | 23632 | -3.7508 | -0.9672 | 1.95E-37 | 9.02E-35 | down-regulated |
| TMEM35 | 59353 | -4.3652 | 2.29171 | 2.00E-37 | 9.02E-35 | down-regulated |
| SPIB | 6689 | -4.7352 | 2.37819 | 4.76E-37 | 2.10E-34 | down-regulated |
| PKIB | 5570 | -3.7298 | 4.20407 | 5.92E-37 | 2.54E-34 | down-regulated |
| MAL | 4118 | -4.3916 | 0.16649 | 6.04E-37 | 2.54E-34 | down-regulated |
| SDPR | 8436 | -3.3334 | 4.21723 | 6.19E-37 | 2.54E-34 | down-regulated |
| FAM129A | 116496 | -3.6858 | 5.30575 | 8.59E-37 | 3.46E-34 | down-regulated |
| PTGS1 | 5742 | -3.0382 | 5.14305 | 9.19E-37 | 3.62E-34 | down-regulated |
| ANGPTL1 | 9068 | -4.9709 | 2.60854 | 2.60E-36 | 1.00E-33 | down-regulated |
| ETV4 | 2118 | 5.16662 | 6.96083 | 1.27E-35 | 4.80E-33 | up-regulated |
| CLDN23 | 137075 | -2.9338 | 4.6555 | 4.68E-35 | 1.69E-32 | down-regulated |
| BAI3 | 577 | -4.7231 | -0.929 | 5.92E-35 | 2.10E-32 | down-regulated |
| CNTN2 | 6900 | -4.7479 | -0.5526 | 7.50E-35 | 2.61E-32 | down-regulated |
| MS4A10 | 341116 | -4.7685 | -2.7806 | 1.02E-34 | 3.49E-32 | down-regulated |
| CA7 | 766 | -5.6083 | 3.55686 | 2.05E-34 | 6.87E-32 | down-regulated |
| DAND5 | 199699 | -4.139 | -1.941 | 2.45E-34 | 8.07E-32 | down-regulated |
| PDK4 | 5166 | -3.0546 | 5.33471 | 3.38E-34 | 1.09E-31 | down-regulated |
| CAV1 | 857 | -2.7069 | 6.13377 | 8.94E-34 | 2.84E-31 | down-regulated |
| ATP6V1G2 | 534 | -2.9076 | -0.7558 | 9.23E-34 | 2.88E-31 | down-regulated |
| NECAB1 | 64168 | -3.6253 | 1.15619 | 9.71E-34 | 2.98E-31 | down-regulated |
| GLIPR2 | 152007 | -2.4309 | 4.51245 | 9.91E-34 | 2.99E-31 | down-regulated |
| LOC572558 | 572558 | -6.0852 | -0.5942 | 1.09E-33 | 3.17E-31 | down-regulated |
| PYGM | 5837 | -4.5156 | 1.57955 | 1.26E-33 | 3.63E-31 | down-regulated |
| SVIL | 6840 | -2.3487 | 6.95076 | 1.43E-33 | 4.04E-31 | down-regulated |
| ATP1A2 | 477 | -5.3922 | 3.1917 | 3.03E-33 | 8.17E-31 | down-regulated |
| KRT80 | 144501 | 6.50893 | 5.49225 | 4.23E-33 | 1.12E-30 | up-regulated |
| CNR1 | 1268 | -4.6016 | 1.02277 | 5.52E-33 | 1.45E-30 | down-regulated |
| DPT | 1805 | -4.6644 | 4.77014 | 5.94E-33 | 1.54E-30 | down-regulated |
| AFF3 | 3899 | -3.5832 | 1.24519 | 7.28E-33 | 1.85E-30 | down-regulated |
| CFL2 | 1073 | -3.0851 | 4.66579 | 8.20E-33 | 2.06E-30 | down-regulated |
| MAB21L1 | 4081 | -4.2491 | -1.8917 | 1.12E-32 | 2.77E-30 | down-regulated |
| NRG2 | 9542 | -4.3454 | -2.109 | 1.15E-32 | 2.80E-30 | down-regulated |
| GCNT2 | 2651 | -3.2411 | 1.67452 | 1.93E-32 | 4.65E-30 | down-regulated |
| LRP1B | 53353 | -4.3737 | -2.507 | 2.44E-32 | 5.82E-30 | down-regulated |
| P2RX2 | 22953 | -4.7706 | -2.5696 | 2.48E-32 | 5.82E-30 | down-regulated |
| MYLK | 4638 | -3.4654 | 8.14204 | 5.66E-32 | 1.31E-29 | down-regulated |
| GNAO1 | 2775 | -3.7832 | 3.19105 | 1.63E-31 | 3.74E-29 | down-regulated |
| NGB | 58157 | -6.0556 | -1.462 | 1.67E-31 | 3.77E-29 | down-regulated |
| STMN4 | 81551 | -4.7987 | -2.2955 | 1.71E-31 | 3.82E-29 | down-regulated |
| CADM3 | 57863 | -4.8948 | 2.51347 | 2.55E-31 | 5.63E-29 | down-regulated |
| PGM5P2 | 595135 | -3.9053 | -1.1964 | 2.59E-31 | 5.65E-29 | down-regulated |
| ASPA | 443 | -4.2253 | -0.9835 | 2.93E-31 | 6.30E-29 | down-regulated |
| LIFR | 3977 | -3.2809 | 2.8849 | 3.09E-31 | 6.58E-29 | down-regulated |
| C2orf88 | 84281 | -3.6289 | 4.19594 | 3.61E-31 | 7.59E-29 | down-regulated |
| MAMDC2 | 256691 | -4.9093 | 2.46025 | 3.82E-31 | 7.95E-29 | down-regulated |
| PMP2 | 5375 | -4.9993 | -1.5701 | 6.58E-31 | 1.34E-28 | down-regulated |
| FAM151A | 338094 | -4.2966 | -0.3794 | 9.09E-31 | 1.83E-28 | down-regulated |
| ANKS1B | 56899 | -4.1568 | -1.3623 | 9.18E-31 | 1.83E-28 | down-regulated |
| AADACL2 | 344752 | -5.4007 | -3.6631 | 9.66E-31 | 1.90E-28 | down-regulated |
| PPP1R12B | 4660 | -3.0582 | 7.01208 | 9.83E-31 | 1.91E-28 | down-regulated |
| TMEM100 | 55273 | -3.6025 | 1.03468 | 1.52E-30 | 2.93E-28 | down-regulated |
| RAB9B | 51209 | -3.1011 | -1.1221 | 1.97E-30 | 3.76E-28 | down-regulated |
| CA2 | 760 | -4.6702 | 7.37796 | 2.61E-30 | 4.91E-28 | down-regulated |
| FGL2 | 10875 | -2.9528 | 5.21432 | 2.83E-30 | 5.28E-28 | down-regulated |
| PKHD1L1 | 93035 | -4.1984 | -0.7629 | 4.08E-30 | 7.54E-28 | down-regulated |
| FHL1 | 2273 | -3.5166 | 6.26931 | 6.20E-30 | 1.13E-27 | down-regulated |
| KCNMB2 | 10242 | -3.4306 | -1.497 | 6.70E-30 | 1.21E-27 | down-regulated |
| SLC25A34 | 284723 | -3.2151 | 1.07336 | 7.13E-30 | 1.28E-27 | down-regulated |
| RXRG | 6258 | -4.4502 | -1.3194 | 7.72E-30 | 1.37E-27 | down-regulated |
| CHAT | 1103 | -4.6299 | -3.8262 | 8.54E-30 | 1.50E-27 | down-regulated |
| TPM1 | 7168 | -2.1354 | 8.84051 | 9.37E-30 | 1.63E-27 | down-regulated |
| NEGR1 | 257194 | -3.2657 | 3.26638 | 1.18E-29 | 2.04E-27 | down-regulated |
| NAP1L2 | 4674 | -3.8017 | 0.2848 | 1.23E-29 | 2.09E-27 | down-regulated |
| SMPDL3A | 10924 | -2.4688 | 4.89394 | 1.80E-29 | 3.04E-27 | down-regulated |
| RERGL | 79785 | -5.7367 | -0.2635 | 2.62E-29 | 4.40E-27 | down-regulated |
| MYL9 | 10398 | -3.4192 | 8.88298 | 2.65E-29 | 4.40E-27 | down-regulated |
| CFD | 1675 | -3.6063 | 4.43652 | 2.86E-29 | 4.70E-27 | down-regulated |
| FAM107A | 11170 | -3.2162 | 2.55583 | 3.04E-29 | 4.96E-27 | down-regulated |
| FOXQ1 | 94234 | 7.17778 | 6.0555 | 3.55E-29 | 5.74E-27 | up-regulated |
| HSPB8 | 26353 | -3.9327 | 4.89825 | 9.68E-29 | 1.55E-26 | down-regulated |
| HPSE2 | 60495 | -3.8876 | -1.2076 | 1.82E-28 | 2.86E-26 | down-regulated |
| C7orf58 | 79974 | -3.0115 | 4.01684 | 4.21E-28 | 6.56E-26 | down-regulated |
| SGK1 | 6446 | -2.6626 | 5.96382 | 4.37E-28 | 6.76E-26 | down-regulated |
| MORN5 | 254956 | -6.107 | -0.4877 | 6.18E-28 | 9.47E-26 | down-regulated |
| NLGN1 | 22871 | -4.5453 | -1.0731 | 6.26E-28 | 9.52E-26 | down-regulated |
| PDE5A | 8654 | -2.5047 | 5.15287 | 6.88E-28 | 1.04E-25 | down-regulated |
| KIAA2022 | 340533 | -3.852 | -0.5692 | 8.43E-28 | 1.26E-25 | down-regulated |
| OLFM3 | 118427 | -4.501 | -2.3486 | 9.50E-28 | 1.41E-25 | down-regulated |
| SOX10 | 6663 | -4.7039 | 0.67409 | 1.14E-27 | 1.68E-25 | down-regulated |
| CAP2 | 10486 | -2.9801 | 3.07223 | 1.19E-27 | 1.74E-25 | down-regulated |
| SYNPO2 | 171024 | -4.0341 | 7.26409 | 1.64E-27 | 2.37E-25 | down-regulated |
| MAB21L2 | 10586 | -4.0584 | 4.37747 | 1.89E-27 | 2.72E-25 | down-regulated |
| TNXB | 7148 | -3.7268 | 5.10488 | 2.28E-27 | 3.25E-25 | down-regulated |
| S100B | 6285 | -3.1911 | 2.06918 | 2.43E-27 | 3.43E-25 | down-regulated |
| FAM46B | 115572 | -3.392 | 1.32741 | 2.67E-27 | 3.74E-25 | down-regulated |
| SMYD1 | 150572 | -5.1754 | 0.58256 | 2.69E-27 | 3.74E-25 | down-regulated |
| SH3BGR | 6450 | -2.7283 | 1.47801 | 3.52E-27 | 4.86E-25 | down-regulated |
| RALYL | 138046 | -4.6036 | -3.73 | 3.55E-27 | 4.86E-25 | down-regulated |
| CTNNA3 | 29119 | -4.2503 | -2.7907 | 3.69E-27 | 5.02E-25 | down-regulated |
| LOC401093 | 401093 | -2.8229 | 2.96794 | 3.86E-27 | 5.21E-25 | down-regulated |
| XKR4 | 114786 | -5.0024 | -2.3143 | 4.00E-27 | 5.36E-25 | down-regulated |
| VSTM2A | 222008 | -5.3304 | -0.4891 | 4.08E-27 | 5.43E-25 | down-regulated |
| PGM5 | 5239 | -3.9375 | 5.15206 | 6.00E-27 | 7.86E-25 | down-regulated |
| EPHA6 | 285220 | -5.4957 | -1.2594 | 6.19E-27 | 8.05E-25 | down-regulated |
| ARL14 | 80117 | -2.8152 | 3.85055 | 7.23E-27 | 9.25E-25 | down-regulated |
| ANK2 | 287 | -3.5619 | 3.3865 | 7.26E-27 | 9.25E-25 | down-regulated |
| GNG3 | 2785 | -2.9548 | -1.2453 | 8.40E-27 | 1.06E-24 | down-regulated |
| KIAA1644 | 85352 | -3.5733 | 2.49464 | 9.92E-27 | 1.25E-24 | down-regulated |
| GRIN2D | 2906 | 5.85815 | 4.99526 | 1.03E-26 | 1.28E-24 | up-regulated |
| LMO3 | 55885 | -4.4342 | 3.23945 | 1.33E-26 | 1.64E-24 | down-regulated |
| RNF150 | 57484 | -4.0195 | 1.20491 | 1.52E-26 | 1.88E-24 | down-regulated |
| DIXDC1 | 85458 | -2.0619 | 4.4423 | 2.58E-26 | 3.15E-24 | down-regulated |
| CHODL | 140578 | -4.1767 | 0.21025 | 2.74E-26 | 3.33E-24 | down-regulated |
| PTN | 5764 | -2.8013 | 2.49679 | 2.78E-26 | 3.33E-24 | down-regulated |
| GLDN | 342035 | -3.407 | 2.5134 | 2.78E-26 | 3.33E-24 | down-regulated |
| C20orf200 | 253868 | -4.0044 | -0.5859 | 2.82E-26 | 3.35E-24 | down-regulated |
| ZMAT4 | 79698 | -4.2863 | -2.8715 | 3.41E-26 | 4.03E-24 | down-regulated |
| ZNF781 | 163115 | -2.6051 | -0.7555 | 3.91E-26 | 4.59E-24 | down-regulated |
| SYNM | 23336 | -4.3904 | 7.15354 | 4.24E-26 | 4.95E-24 | down-regulated |
| KHDRBS2 | 202559 | -4.5936 | -3.7071 | 4.41E-26 | 5.12E-24 | down-regulated |
| SMTN | 6525 | -2.0661 | 7.21678 | 4.73E-26 | 5.45E-24 | down-regulated |
| ATP2B4 | 493 | -2.4695 | 6.74661 | 5.15E-26 | 5.90E-24 | down-regulated |
| CNGA3 | 1261 | -4.3337 | -0.2639 | 5.47E-26 | 6.23E-24 | down-regulated |
| PDZRN4 | 29951 | -4.6245 | 1.95029 | 5.75E-26 | 6.50E-24 | down-regulated |
| P2RY14 | 9934 | -2.8198 | 1.86684 | 7.36E-26 | 8.27E-24 | down-regulated |
| PLCD4 | 84812 | -2.2053 | 1.56391 | 7.61E-26 | 8.49E-24 | down-regulated |
| ENPP6 | 133121 | -3.2273 | -1.0351 | 8.64E-26 | 9.59E-24 | down-regulated |
| ABCG2 | 9429 | -3.8261 | 3.17662 | 9.39E-26 | 1.04E-23 | down-regulated |
| PDE2A | 5138 | -2.9506 | 2.51108 | 9.46E-26 | 1.04E-23 | down-regulated |
| ELAVL4 | 1996 | -3.8538 | -1.4593 | 1.30E-25 | 1.41E-23 | down-regulated |
| GRIA1 | 2890 | -3.9878 | -2.3348 | 2.34E-25 | 2.53E-23 | down-regulated |
| PIRT | 644139 | -4.3929 | -0.11 | 2.48E-25 | 2.67E-23 | down-regulated |
| NFE2L3 | 9603 | 2.97972 | 7.39335 | 3.56E-25 | 3.81E-23 | up-regulated |
| GNG7 | 2788 | -2.7028 | 1.96656 | 3.90E-25 | 4.15E-23 | down-regulated |
| TP53INP2 | 58476 | -2.1626 | 6.05439 | 4.14E-25 | 4.38E-23 | down-regulated |
| EML1 | 2009 | -2.729 | 3.98979 | 4.24E-25 | 4.46E-23 | down-regulated |
| TFAP2B | 7021 | -5.5535 | -3.597 | 4.58E-25 | 4.79E-23 | down-regulated |
| SLC2A4 | 6517 | -2.9982 | 2.76673 | 4.75E-25 | 4.92E-23 | down-regulated |
| KANK2 | 25959 | -2.1919 | 6.43106 | 4.75E-25 | 4.92E-23 | down-regulated |
| MUSK | 4593 | -4.2932 | -1.7454 | 5.08E-25 | 5.22E-23 | down-regulated |
| ADAMDEC1 | 27299 | -3.6279 | 5.71581 | 6.06E-25 | 6.19E-23 | down-regulated |
| SGCG | 6445 | -4.5691 | -2.0588 | 6.78E-25 | 6.90E-23 | down-regulated |
| FAM19A2 | 338811 | -2.5062 | -1.0452 | 7.02E-25 | 7.10E-23 | down-regulated |
| SPOCK3 | 50859 | -5.3626 | -1.2081 | 7.27E-25 | 7.31E-23 | down-regulated |
| GALR1 | 2587 | -4.0399 | -3.4843 | 7.31E-25 | 7.31E-23 | down-regulated |
| GSG1L | 146395 | -3.9953 | -3.222 | 8.04E-25 | 7.99E-23 | down-regulated |
| LYVE1 | 10894 | -4.0719 | 3.2835 | 8.19E-25 | 8.10E-23 | down-regulated |
| CLDN1 | 9076 | 5.17581 | 7.06166 | 8.59E-25 | 8.45E-23 | up-regulated |
| BEND5 | 79656 | -3.2291 | -0.1963 | 8.90E-25 | 8.70E-23 | down-regulated |
| ATP2B3 | 492 | -4.2578 | -2.9492 | 1.07E-24 | 1.04E-22 | down-regulated |
| PEG3 | 5178 | -3.1213 | 1.51575 | 1.78E-24 | 1.71E-22 | down-regulated |
| TCEAL7 | 56849 | -2.7095 | 1.22659 | 2.53E-24 | 2.42E-22 | down-regulated |
| SCGN | 10590 | -4.822 | 1.41899 | 2.61E-24 | 2.49E-22 | down-regulated |
| GSTM5 | 2949 | -3.2727 | 1.32332 | 2.76E-24 | 2.61E-22 | down-regulated |
| UNC80 | 285175 | -3.6805 | -2.749 | 3.52E-24 | 3.32E-22 | down-regulated |
| FAM19A4 | 151647 | -3.9514 | -3.3641 | 3.59E-24 | 3.36E-22 | down-regulated |
| SCN9A | 6335 | -3.4111 | 2.03384 | 3.96E-24 | 3.69E-22 | down-regulated |
| RBM24 | 221662 | -3.2123 | 1.34933 | 4.31E-24 | 4.00E-22 | down-regulated |
| DNER | 92737 | -3.7347 | -0.4157 | 4.55E-24 | 4.20E-22 | down-regulated |
| GNG2 | 54331 | -2.1516 | 3.90424 | 4.65E-24 | 4.25E-22 | down-regulated |
| SSBP2 | 23635 | -2.4751 | 2.30296 | 5.44E-24 | 4.94E-22 | down-regulated |
| GFRA2 | 2675 | -3.3717 | 0.47593 | 5.82E-24 | 5.26E-22 | down-regulated |
| MYT1L | 23040 | -4.1138 | -3.3061 | 9.65E-24 | 8.64E-22 | down-regulated |
| LMOD1 | 25802 | -3.6421 | 6.37049 | 1.08E-23 | 9.59E-22 | down-regulated |
| PDE4D | 5144 | -2.0129 | 4.30545 | 1.11E-23 | 9.80E-22 | down-regulated |
| PSD | 5662 | -4.2085 | 3.71923 | 1.11E-23 | 9.80E-22 | down-regulated |
| PLN | 5350 | -3.8514 | 4.4719 | 1.19E-23 | 1.04E-21 | down-regulated |
| NPAS4 | 266743 | -3.4571 | -2.3367 | 1.21E-23 | 1.06E-21 | down-regulated |
| NKX2-3 | 159296 | -2.9705 | 2.60138 | 1.23E-23 | 1.07E-21 | down-regulated |
| NTN1 | 9423 | -2.8764 | 3.19334 | 1.24E-23 | 1.07E-21 | down-regulated |
| RBM20 | 282996 | -3.4135 | -0.0406 | 1.28E-23 | 1.11E-21 | down-regulated |
| MYH11 | 4629 | -4.3654 | 10.3884 | 1.38E-23 | 1.18E-21 | down-regulated |
| ZDHHC22 | 283576 | -4.3116 | -2.5708 | 1.57E-23 | 1.34E-21 | down-regulated |
| ESM1 | 11082 | 5.45749 | 3.3776 | 1.61E-23 | 1.37E-21 | up-regulated |
| BVES | 11149 | -3.1286 | 2.1303 | 1.62E-23 | 1.37E-21 | down-regulated |
| JUB | 84962 | 2.92924 | 4.69838 | 1.80E-23 | 1.52E-21 | up-regulated |
| ASTN1 | 460 | -4.0434 | -1.5842 | 2.15E-23 | 1.80E-21 | down-regulated |
| ABI3BP | 25890 | -3.489 | 3.79967 | 2.38E-23 | 1.99E-21 | down-regulated |
| SORCS1 | 114815 | -4.4178 | 0.7929 | 2.45E-23 | 2.03E-21 | down-regulated |
| FAM180B | 399888 | -4.897 | -2.4485 | 2.85E-23 | 2.35E-21 | down-regulated |
| LOC284276 | 284276 | -3.1463 | -2.8051 | 3.02E-23 | 2.48E-21 | down-regulated |
| KCTD4 | 386618 | -3.5975 | -2.3195 | 3.04E-23 | 2.48E-21 | down-regulated |
| TAGLN3 | 29114 | -4.4145 | -0.6544 | 3.24E-23 | 2.63E-21 | down-regulated |
| ITM2A | 9452 | -2.7131 | 3.40757 | 3.90E-23 | 3.15E-21 | down-regulated |
| FOXF2 | 2295 | -2.4764 | 3.20042 | 4.33E-23 | 3.48E-21 | down-regulated |
| HAND2 | 9464 | -4.1483 | 2.80805 | 4.49E-23 | 3.59E-21 | down-regulated |
| FBXL22 | 283807 | -2.7701 | 1.25623 | 4.54E-23 | 3.62E-21 | down-regulated |
| GUCA2A | 2980 | -4.6152 | 5.91975 | 4.64E-23 | 3.68E-21 | down-regulated |
| RNF152 | 220441 | -2.9243 | 0.54959 | 4.67E-23 | 3.69E-21 | down-regulated |
| KIAA1199 | 57214 | 5.5464 | 7.35433 | 4.86E-23 | 3.83E-21 | up-regulated |
| ANGPTL7 | 10218 | -5.6289 | 0.2061 | 7.36E-23 | 5.77E-21 | down-regulated |
| C6orf186 | 728464 | -3.9678 | -1.0146 | 8.19E-23 | 6.38E-21 | down-regulated |
| ELANE | 1991 | -3.7819 | -2.9522 | 8.47E-23 | 6.57E-21 | down-regulated |
| HMP19 | 51617 | -4.1887 | 0.1568 | 1.05E-22 | 8.11E-21 | down-regulated |
| SOX15 | 6665 | -2.4319 | -0.5129 | 1.17E-22 | 9.04E-21 | down-regulated |
| ASB2 | 51676 | -3.3088 | 2.98736 | 1.23E-22 | 9.39E-21 | down-regulated |
| LIMS2 | 55679 | -2.7162 | 5.02348 | 1.70E-22 | 1.29E-20 | down-regulated |
| LPAR1 | 1902 | -2.3562 | 3.41325 | 2.22E-22 | 1.68E-20 | down-regulated |
| DLG2 | 1740 | -3.0906 | -0.1732 | 2.33E-22 | 1.76E-20 | down-regulated |
| PPP1R14A | 94274 | -2.7646 | 2.68823 | 2.52E-22 | 1.89E-20 | down-regulated |
| BEX1 | 55859 | -3.6525 | -0.8925 | 2.63E-22 | 1.96E-20 | down-regulated |
| BAALC | 79870 | -2.9769 | -0.1191 | 3.15E-22 | 2.34E-20 | down-regulated |
| NEBL | 10529 | 2.20301 | 6.9366 | 3.80E-22 | 2.80E-20 | up-regulated |
| NCAM1 | 4684 | -2.9841 | 3.12623 | 3.83E-22 | 2.81E-20 | down-regulated |
| TMEFF2 | 23671 | -4.6271 | -2.0859 | 4.26E-22 | 3.12E-20 | down-regulated |
| CXCL12 | 6387 | -2.812 | 5.54283 | 4.31E-22 | 3.14E-20 | down-regulated |
| DNAJB5 | 25822 | -2.6844 | 3.2501 | 4.38E-22 | 3.18E-20 | down-regulated |
| SLC6A6 | 6533 | 3.48855 | 7.29283 | 4.66E-22 | 3.36E-20 | up-regulated |
| LDB3 | 11155 | -4.1114 | 2.3563 | 4.72E-22 | 3.39E-20 | down-regulated |
| SRPX | 8406 | -3.0648 | 3.10184 | 4.96E-22 | 3.55E-20 | down-regulated |
| SCARA5 | 286133 | -4.3051 | 3.68598 | 5.01E-22 | 3.57E-20 | down-regulated |
| DOCK3 | 1795 | -2.6953 | -0.1784 | 6.17E-22 | 4.38E-20 | down-regulated |
| AHCYL2 | 23382 | -2.545 | 7.02767 | 7.20E-22 | 5.09E-20 | down-regulated |
| CNTNAP3 | 79937 | -3.3175 | -0.2189 | 9.53E-22 | 6.69E-20 | down-regulated |
| PRKCB | 5579 | -2.8802 | 3.03714 | 9.65E-22 | 6.73E-20 | down-regulated |
| KIAA1549 | 57670 | 2.42755 | 5.08947 | 9.68E-22 | 6.73E-20 | up-regulated |
| RGS2 | 5997 | -2.5486 | 4.87896 | 1.05E-21 | 7.27E-20 | down-regulated |
| CNGB1 | 1258 | -3.7181 | -2.7829 | 1.08E-21 | 7.41E-20 | down-regulated |
| TMEM72 | 643236 | -4.2832 | -0.0284 | 1.18E-21 | 8.08E-20 | down-regulated |
| NEFM | 4741 | -3.6342 | -1.2611 | 1.29E-21 | 8.80E-20 | down-regulated |
| C16orf89 | 146556 | -4.0122 | 0.8871 | 1.47E-21 | 9.98E-20 | down-regulated |
| KLF4 | 9314 | -2.5421 | 6.73329 | 1.62E-21 | 1.10E-19 | down-regulated |
| IGSF11 | 152404 | -4.0063 | -1.3352 | 1.69E-21 | 1.14E-19 | down-regulated |
| CPEB1 | 64506 | -4.0098 | -1.0341 | 1.72E-21 | 1.16E-19 | down-regulated |
| PDZD4 | 57595 | -3.2771 | 1.58259 | 1.98E-21 | 1.33E-19 | down-regulated |
| TEX11 | 56159 | -3.6728 | -1.1075 | 2.08E-21 | 1.39E-19 | down-regulated |
| MMP28 | 79148 | -3.2912 | 3.77998 | 2.30E-21 | 1.53E-19 | down-regulated |
| SCN2B | 6327 | -3.8908 | -0.0872 | 2.77E-21 | 1.83E-19 | down-regulated |
| RBPMS2 | 348093 | -3.4514 | 2.64459 | 3.22E-21 | 2.12E-19 | down-regulated |
| CCBE1 | 147372 | -4.2838 | 0.79643 | 3.75E-21 | 2.47E-19 | down-regulated |
| KCNA5 | 3741 | -2.7889 | -0.3609 | 3.90E-21 | 2.56E-19 | down-regulated |
| AVPR1B | 553 | -4.1202 | -3.7114 | 3.98E-21 | 2.60E-19 | down-regulated |
| CHL1 | 10752 | -3.0159 | 2.37884 | 4.05E-21 | 2.64E-19 | down-regulated |
| SPHKAP | 80309 | -4.4074 | -3.4189 | 4.29E-21 | 2.78E-19 | down-regulated |
| PKNOX2 | 63876 | -2.8311 | -0.2378 | 4.78E-21 | 3.09E-19 | down-regulated |
| GTF2IRD1 | 9569 | 2.40491 | 6.30135 | 6.09E-21 | 3.92E-19 | up-regulated |
| SLC4A4 | 8671 | -4.485 | 5.51214 | 6.23E-21 | 4.00E-19 | down-regulated |
| RPRM | 56475 | -3.5213 | -0.2874 | 6.92E-21 | 4.41E-19 | down-regulated |
| RNF112 | 7732 | -2.7405 | -0.329 | 6.93E-21 | 4.41E-19 | down-regulated |
| KCNMA1 | 3778 | -3.5728 | 4.99583 | 7.02E-21 | 4.46E-19 | down-regulated |
| CGREF1 | 10669 | 3.32342 | 4.96926 | 8.21E-21 | 5.20E-19 | up-regulated |
| MMRN1 | 22915 | -3.6014 | 3.0012 | 8.71E-21 | 5.49E-19 | down-regulated |
| TCL1A | 8115 | -4.9384 | 0.64872 | 9.37E-21 | 5.88E-19 | down-regulated |
| FUT1 | 2523 | 2.75586 | 3.04422 | 9.51E-21 | 5.95E-19 | up-regulated |
| ST6GALNAC6 | 30815 | -2.7844 | 6.49424 | 1.07E-20 | 6.65E-19 | down-regulated |
| CAND2 | 23066 | -2.41 | 1.04637 | 1.26E-20 | 7.84E-19 | down-regulated |
| MMP27 | 64066 | -4.2893 | -3.8021 | 1.29E-20 | 7.97E-19 | down-regulated |
| JPH4 | 84502 | -2.6593 | -1.0349 | 1.50E-20 | 9.21E-19 | down-regulated |
| MYOM1 | 8736 | -3.5361 | 2.23005 | 1.79E-20 | 1.10E-18 | down-regulated |
| SSTR2 | 6752 | -2.7311 | -0.2393 | 1.85E-20 | 1.13E-18 | down-regulated |
| C14orf176 | 643382 | -2.5422 | 3.07598 | 1.94E-20 | 1.18E-18 | down-regulated |
| KCNA1 | 3736 | -4.9238 | -1.6823 | 1.98E-20 | 1.20E-18 | down-regulated |
| ABCA8 | 10351 | -4.3754 | 2.97148 | 2.00E-20 | 1.20E-18 | down-regulated |
| KCNB1 | 3745 | -4.3482 | -0.9098 | 2.00E-20 | 1.20E-18 | down-regulated |
| MSTN | 2660 | -3.2877 | -1.6867 | 2.02E-20 | 1.21E-18 | down-regulated |
| ATCAY | 85300 | -3.7226 | -1.5879 | 2.07E-20 | 1.23E-18 | down-regulated |
| SALL4 | 57167 | 5.65317 | 1.99083 | 2.33E-20 | 1.39E-18 | up-regulated |
| SCN11A | 11280 | -3.2694 | -1.2702 | 2.39E-20 | 1.42E-18 | down-regulated |
| TSPAN7 | 7102 | -2.4618 | 5.01476 | 2.60E-20 | 1.53E-18 | down-regulated |
| NTNG1 | 22854 | -4.1038 | -1.0448 | 2.69E-20 | 1.58E-18 | down-regulated |
| OSTBETA | 123264 | -3.1823 | 2.70689 | 2.73E-20 | 1.60E-18 | down-regulated |
| HSPB6 | 126393 | -3.4499 | 5.19725 | 2.74E-20 | 1.60E-18 | down-regulated |
| ATP1B2 | 482 | -2.5259 | 0.51941 | 3.44E-20 | 2.00E-18 | down-regulated |
| GEFT | 115557 | -2.7384 | 3.74986 | 4.06E-20 | 2.33E-18 | down-regulated |
| SPTBN2 | 6712 | 3.00953 | 5.15839 | 4.30E-20 | 2.46E-18 | up-regulated |
| GYLTL1B | 120071 | 4.50113 | 5.36552 | 4.54E-20 | 2.58E-18 | up-regulated |
| MT1M | 4499 | -4.283 | 3.29865 | 4.86E-20 | 2.75E-18 | down-regulated |
| FILIP1 | 27145 | -2.777 | 1.80956 | 5.61E-20 | 3.17E-18 | down-regulated |
| TRPM6 | 140803 | -3.4842 | 4.26601 | 5.90E-20 | 3.32E-18 | down-regulated |
| SORBS1 | 10580 | -2.4072 | 6.71129 | 6.62E-20 | 3.72E-18 | down-regulated |
| CDKN2BAS | 100048912 | -4.8411 | 2.74401 | 6.97E-20 | 3.90E-18 | down-regulated |
| LRRC8E | 80131 | 2.75264 | 4.0127 | 8.07E-20 | 4.49E-18 | up-regulated |
| KRT24 | 192666 | -7.5093 | -0.9205 | 8.33E-20 | 4.61E-18 | down-regulated |
| DHRS11 | 79154 | -2.1169 | 5.3182 | 9.21E-20 | 5.08E-18 | down-regulated |
| PALLD | 23022 | -2.1057 | 7.43031 | 9.32E-20 | 5.12E-18 | down-regulated |
| CDH19 | 28513 | -4.6403 | 0.33236 | 9.66E-20 | 5.29E-18 | down-regulated |
| AQP8 | 343 | -5.2843 | 5.57229 | 1.06E-19 | 5.77E-18 | down-regulated |
| CLEC9A | 283420 | -3.3607 | -2.1123 | 1.12E-19 | 6.07E-18 | down-regulated |
| DMD | 1756 | -2.4857 | 4.54761 | 1.27E-19 | 6.85E-18 | down-regulated |
| ACTG2 | 72 | -3.8775 | 8.60262 | 1.27E-19 | 6.85E-18 | down-regulated |
| LOC645323 | 645323 | -3.7261 | -2.612 | 1.72E-19 | 9.18E-18 | down-regulated |
| FXYD6 | 53826 | -2.7488 | 5.0006 | 1.96E-19 | 1.04E-17 | down-regulated |
| FLNC | 2318 | -3.2277 | 6.83679 | 2.02E-19 | 1.07E-17 | down-regulated |
| SLC5A7 | 60482 | -4.7037 | -1.1429 | 2.53E-19 | 1.33E-17 | down-regulated |
| PHOX2B | 8929 | -4.5818 | -1.1921 | 2.54E-19 | 1.33E-17 | down-regulated |
| ABCA9 | 10350 | -3.7989 | 0.91217 | 2.67E-19 | 1.40E-17 | down-regulated |
| GPM6A | 2823 | -5.0792 | 2.17029 | 2.75E-19 | 1.44E-17 | down-regulated |
| JAZF1 | 221895 | -2.0804 | 3.35054 | 2.97E-19 | 1.54E-17 | down-regulated |
| BMP3 | 651 | -4.3973 | 3.04288 | 3.07E-19 | 1.59E-17 | down-regulated |
| NR3C2 | 4306 | -2.624 | 4.97908 | 3.30E-19 | 1.70E-17 | down-regulated |
| MACC1 | 346389 | 2.88157 | 6.54444 | 3.35E-19 | 1.72E-17 | up-regulated |
| MDFI | 4188 | 3.45158 | 3.6547 | 4.02E-19 | 2.05E-17 | up-regulated |
| OGN | 4969 | -4.2965 | 3.60903 | 4.15E-19 | 2.12E-17 | down-regulated |
| SLCO4A1 | 28231 | 3.47566 | 6.09459 | 4.85E-19 | 2.46E-17 | up-regulated |
| MYOCD | 93649 | -3.2937 | 3.15693 | 4.89E-19 | 2.47E-17 | down-regulated |
| ANGPTL5 | 253935 | -4.0252 | -3.0142 | 4.90E-19 | 2.47E-17 | down-regulated |
| PHGR1 | 644844 | -2.382 | 6.84957 | 5.57E-19 | 2.79E-17 | down-regulated |
| FCRL4 | 83417 | -3.9733 | -1.986 | 6.83E-19 | 3.42E-17 | down-regulated |
| OTOP2 | 92736 | -7.2629 | 2.4215 | 7.75E-19 | 3.86E-17 | down-regulated |
| HTR7 | 3363 | -2.7445 | -1.3337 | 8.07E-19 | 4.00E-17 | down-regulated |
| ACAN | 176 | 4.4144 | 3.72725 | 8.88E-19 | 4.39E-17 | up-regulated |
| SLC17A8 | 246213 | -4.8284 | -2.0223 | 1.10E-18 | 5.35E-17 | down-regulated |
| GRIA4 | 2893 | -3.0128 | -1.3954 | 1.25E-18 | 6.07E-17 | down-regulated |
| HPGD | 3248 | -2.6624 | 5.60775 | 1.27E-18 | 6.15E-17 | down-regulated |
| RGS9 | 8787 | -2.903 | 0.46045 | 1.30E-18 | 6.29E-17 | down-regulated |
| TNS1 | 7145 | -2.8183 | 7.68628 | 1.32E-18 | 6.38E-17 | down-regulated |
| WNT2 | 7472 | 5.54603 | 3.40545 | 1.35E-18 | 6.48E-17 | up-regulated |
| CDKL1 | 8814 | -2.1367 | 0.71055 | 1.36E-18 | 6.55E-17 | down-regulated |
| SFTPC | 6440 | -6.822 | -1.7975 | 1.39E-18 | 6.65E-17 | down-regulated |
| FLNA | 2316 | -2.4971 | 10.129 | 1.50E-18 | 7.18E-17 | down-regulated |
| CASR | 846 | -3.5247 | -2.7998 | 1.59E-18 | 7.53E-17 | down-regulated |
| GFRA1 | 2674 | -3.2591 | 2.83322 | 1.59E-18 | 7.55E-17 | down-regulated |
| SIM2 | 6493 | 4.31782 | 3.92466 | 1.60E-18 | 7.57E-17 | up-regulated |
| LAMA1 | 284217 | -3.0351 | 2.39496 | 1.63E-18 | 7.69E-17 | down-regulated |
| TPM2 | 7169 | -2.7605 | 8.07814 | 1.66E-18 | 7.79E-17 | down-regulated |
| KCNMB1 | 3779 | -3.3029 | 3.80626 | 1.77E-18 | 8.30E-17 | down-regulated |
| MTHFD1L | 25902 | 2.24607 | 5.62979 | 1.78E-18 | 8.32E-17 | up-regulated |
| SORBS2 | 8470 | -2.4073 | 5.2227 | 1.81E-18 | 8.43E-17 | down-regulated |
| SHROOM4 | 57477 | 2.36218 | 5.15251 | 1.90E-18 | 8.82E-17 | up-regulated |
| DACT3 | 147906 | -2.7932 | 3.04981 | 1.96E-18 | 9.07E-17 | down-regulated |
| CNN1 | 1264 | -3.6957 | 7.25182 | 2.06E-18 | 9.54E-17 | down-regulated |
| TMEM155 | 132332 | -3.0633 | -2.1284 | 2.14E-18 | 9.88E-17 | down-regulated |
| LRCH2 | 57631 | -2.7924 | 1.31019 | 2.19E-18 | 1.01E-16 | down-regulated |
| STAP1 | 26228 | -3.4604 | -0.8101 | 2.21E-18 | 1.01E-16 | down-regulated |
| PGR | 5241 | -3.1176 | 1.17198 | 2.33E-18 | 1.07E-16 | down-regulated |
| ADAMTSL3 | 57188 | -3.4537 | 2.71292 | 2.48E-18 | 1.13E-16 | down-regulated |
| ANPEP | 290 | -3.1323 | 6.26169 | 2.52E-18 | 1.14E-16 | down-regulated |
| LOC92659 | 92659 | 3.00487 | 2.55349 | 2.61E-18 | 1.18E-16 | up-regulated |
| NKD2 | 85409 | 4.5256 | 4.65502 | 3.41E-18 | 1.54E-16 | up-regulated |
| GCG | 2641 | -6.1594 | 3.87726 | 3.52E-18 | 1.58E-16 | down-regulated |
| ASCL2 | 430 | 3.94633 | 6.75218 | 4.00E-18 | 1.80E-16 | up-regulated |
| SNAP91 | 9892 | -3.9087 | -1.5287 | 4.01E-18 | 1.80E-16 | down-regulated |
| MSRB3 | 253827 | -2.7133 | 5.301 | 4.17E-18 | 1.86E-16 | down-regulated |
| UNC5D | 137970 | -3.5774 | -1.993 | 4.33E-18 | 1.93E-16 | down-regulated |
| TESC | 54997 | 4.58666 | 5.74783 | 4.42E-18 | 1.97E-16 | up-regulated |
| ALPI | 248 | -3.8204 | 2.46966 | 4.59E-18 | 2.03E-16 | down-regulated |
| FOXD3 | 27022 | -4.0048 | -1.7249 | 4.89E-18 | 2.16E-16 | down-regulated |
| LRRC3B | 116135 | -3.5538 | -3.1246 | 5.62E-18 | 2.47E-16 | down-regulated |
| GREM2 | 64388 | -3.4306 | 3.50165 | 5.71E-18 | 2.50E-16 | down-regulated |
| DPEP1 | 1800 | 7.50441 | 8.04118 | 5.95E-18 | 2.59E-16 | up-regulated |
| TEAD4 | 7004 | 2.40387 | 4.79156 | 6.13E-18 | 2.67E-16 | up-regulated |
| SOX9 | 6662 | 2.21737 | 8.12135 | 6.67E-18 | 2.89E-16 | up-regulated |
| PCP4L1 | 654790 | -3.6833 | -0.0604 | 6.70E-18 | 2.90E-16 | down-regulated |
| CPB1 | 1360 | -5.5676 | -0.2301 | 6.73E-18 | 2.90E-16 | down-regulated |
| RGS13 | 6003 | -3.9305 | -0.1899 | 7.37E-18 | 3.15E-16 | down-regulated |
| AICDA | 57379 | -4.8002 | -2.1207 | 1.10E-17 | 4.67E-16 | down-regulated |
| EPB41L3 | 23136 | -2.5321 | 4.28511 | 1.15E-17 | 4.88E-16 | down-regulated |
| SULT1A2 | 6799 | -3.1265 | 1.79082 | 1.25E-17 | 5.32E-16 | down-regulated |
| FEV | 54738 | -4.6 | -0.9516 | 1.27E-17 | 5.38E-16 | down-regulated |
| BCHE | 590 | -4.1936 | 2.38383 | 1.32E-17 | 5.55E-16 | down-regulated |
| FMN2 | 56776 | -4.1236 | 0.5221 | 1.51E-17 | 6.34E-16 | down-regulated |
| HMGCLL1 | 54511 | -3.0784 | -2.0819 | 1.70E-17 | 7.12E-16 | down-regulated |
| ACSBG1 | 23205 | -2.4252 | -1.0339 | 1.80E-17 | 7.52E-16 | down-regulated |
| MEX3A | 92312 | 3.25948 | 4.4519 | 1.85E-17 | 7.68E-16 | up-regulated |
| TAGLN | 6876 | -2.8884 | 9.32263 | 1.91E-17 | 7.92E-16 | down-regulated |
| SGCE | 8910 | -2.1278 | 3.35774 | 1.97E-17 | 8.17E-16 | down-regulated |
| DCLK1 | 9201 | -2.9904 | 1.59562 | 2.02E-17 | 8.33E-16 | down-regulated |
| SLC8A1 | 6546 | -2.0417 | 3.82998 | 2.08E-17 | 8.56E-16 | down-regulated |
| ADCY2 | 108 | -2.8949 | 0.81926 | 2.13E-17 | 8.75E-16 | down-regulated |
| ITIH5 | 80760 | -2.2806 | 4.86244 | 2.36E-17 | 9.67E-16 | down-regulated |
| ARMCX1 | 51309 | -2.2327 | 2.75884 | 2.40E-17 | 9.82E-16 | down-regulated |
| SDCBP2 | 27111 | -2.3837 | 6.00742 | 2.40E-17 | 9.82E-16 | down-regulated |
| CALY | 50632 | -4.0037 | -2.0768 | 2.47E-17 | 1.01E-15 | down-regulated |
| AKAP6 | 9472 | -2.4256 | 2.26399 | 2.48E-17 | 1.01E-15 | down-regulated |
| MGC42105 | 167359 | -2.4522 | -2.1773 | 2.48E-17 | 1.01E-15 | down-regulated |
| HPGDS | 27306 | -2.857 | 0.11205 | 2.61E-17 | 1.05E-15 | down-regulated |
| ZEB1 | 6935 | -2.1836 | 4.9064 | 3.01E-17 | 1.21E-15 | down-regulated |
| LONRF2 | 164832 | -4.2426 | 1.37608 | 3.25E-17 | 1.30E-15 | down-regulated |
| RND2 | 8153 | -2.7209 | 0.03702 | 3.40E-17 | 1.36E-15 | down-regulated |
| CLEC10A | 10462 | -3.168 | 2.08736 | 3.41E-17 | 1.36E-15 | down-regulated |
| TRIB3 | 57761 | 3.65789 | 6.47736 | 3.57E-17 | 1.42E-15 | up-regulated |
| PVT1 | 5820 | 3.10033 | 2.98048 | 3.89E-17 | 1.55E-15 | up-regulated |
| GRIK5 | 2901 | -3.5043 | 0.85039 | 3.99E-17 | 1.58E-15 | down-regulated |
| CHRM4 | 1132 | -2.6259 | -2.0271 | 4.28E-17 | 1.69E-15 | down-regulated |
| KIAA1045 | 23349 | -3.2126 | -1.6963 | 4.29E-17 | 1.70E-15 | down-regulated |
| LGALS2 | 3957 | -3.5128 | 3.81573 | 4.46E-17 | 1.76E-15 | down-regulated |
| SLC8A2 | 6543 | -3.372 | 0.50784 | 4.78E-17 | 1.88E-15 | down-regulated |
| AGPAT9 | 84803 | -2.3295 | 3.39961 | 5.15E-17 | 2.02E-15 | down-regulated |
| GPR12 | 2835 | -4.0165 | -3.4128 | 5.25E-17 | 2.06E-15 | down-regulated |
| SLC7A14 | 57709 | -4.1916 | -1.073 | 5.28E-17 | 2.06E-15 | down-regulated |
| C2orf40 | 84417 | -4.178 | 0.554 | 5.50E-17 | 2.15E-15 | down-regulated |
| FBXO32 | 114907 | -2.0477 | 4.75073 | 5.58E-17 | 2.17E-15 | down-regulated |
| CCDC68 | 80323 | -2.1332 | 3.76576 | 5.86E-17 | 2.28E-15 | down-regulated |
| FCER1A | 2205 | -3.2278 | 0.39374 | 5.93E-17 | 2.30E-15 | down-regulated |
| PADI2 | 11240 | -2.956 | 6.58414 | 6.32E-17 | 2.44E-15 | down-regulated |
| SLC26A3 | 1811 | -3.8205 | 8.92864 | 6.35E-17 | 2.45E-15 | down-regulated |
| SLC26A2 | 1836 | -3.1386 | 8.22549 | 7.16E-17 | 2.76E-15 | down-regulated |
| CELSR3 | 1951 | 3.35069 | 4.59281 | 7.24E-17 | 2.78E-15 | up-regulated |
| GFRA3 | 2676 | -3.4026 | 0.74631 | 7.44E-17 | 2.85E-15 | down-regulated |
| MS4A12 | 54860 | -5.1066 | 5.80641 | 7.50E-17 | 2.87E-15 | down-regulated |
| TCEAL5 | 340543 | -2.4398 | -1.7502 | 8.93E-17 | 3.41E-15 | down-regulated |
| FAM70A | 55026 | -2.8771 | 0.62942 | 9.02E-17 | 3.44E-15 | down-regulated |
| ENTPD5 | 957 | -2.0297 | 4.89128 | 9.07E-17 | 3.45E-15 | down-regulated |
| C14orf139 | 79686 | -2.0135 | 1.4085 | 9.41E-17 | 3.57E-15 | down-regulated |
| SLITRK2 | 84631 | -3.3634 | -2.4721 | 9.68E-17 | 3.66E-15 | down-regulated |
| RPH3A | 22895 | -2.9027 | -2.8536 | 9.82E-17 | 3.70E-15 | down-regulated |
| PDX1 | 3651 | 4.62863 | 4.30279 | 9.91E-17 | 3.73E-15 | up-regulated |
| PRICKLE2 | 166336 | -2.1645 | 3.72674 | 9.93E-17 | 3.73E-15 | down-regulated |
| CCL23 | 6368 | -4.1227 | -1.0009 | 1.03E-16 | 3.86E-15 | down-regulated |
| IL6R | 3570 | -2.4051 | 3.45193 | 1.17E-16 | 4.39E-15 | down-regulated |
| TGFBI | 7045 | 3.53083 | 10.0632 | 1.20E-16 | 4.46E-15 | up-regulated |
| KIAA1462 | 57608 | -2.3376 | 5.05732 | 1.23E-16 | 4.56E-15 | down-regulated |
| CBX2 | 84733 | 4.07102 | 4.2473 | 1.23E-16 | 4.58E-15 | up-regulated |
| C15orf48 | 84419 | -2.5951 | 7.43014 | 1.24E-16 | 4.62E-15 | down-regulated |
| PER3 | 8863 | -2.1257 | 3.69586 | 1.35E-16 | 4.97E-15 | down-regulated |
| CLCNKB | 1188 | -4.0924 | -1.73 | 1.41E-16 | 5.19E-15 | down-regulated |
| ATP11A | 23250 | 2.51737 | 6.69882 | 1.42E-16 | 5.23E-15 | up-regulated |
| SH2D7 | 646892 | -3.2814 | -1.5356 | 1.53E-16 | 5.62E-15 | down-regulated |
| SLC16A12 | 387700 | -2.8581 | -1.9975 | 1.62E-16 | 5.92E-15 | down-regulated |
| MRGPRF | 116535 | -2.7807 | 4.239 | 1.67E-16 | 6.09E-15 | down-regulated |
| EVX2 | 344191 | -4.1254 | -3.2471 | 1.68E-16 | 6.09E-15 | down-regulated |
| ADAMTS8 | 11095 | -2.7433 | 1.33678 | 1.74E-16 | 6.31E-15 | down-regulated |
| KIAA0408 | 9729 | -6.0265 | 0.92239 | 1.76E-16 | 6.36E-15 | down-regulated |
| SLC9A9 | 285195 | -2.3421 | 1.97611 | 2.00E-16 | 7.19E-15 | down-regulated |
| C7 | 730 | -3.8661 | 4.93664 | 2.00E-16 | 7.19E-15 | down-regulated |
| SULT2B1 | 6820 | 4.40174 | 4.61931 | 2.05E-16 | 7.34E-15 | up-regulated |
| SOX4 | 6659 | 2.16039 | 7.55906 | 2.05E-16 | 7.34E-15 | up-regulated |
| PPM1H | 57460 | 2.21709 | 5.6843 | 2.13E-16 | 7.59E-15 | up-regulated |
| SPEG | 10290 | -3.288 | 3.66934 | 2.18E-16 | 7.76E-15 | down-regulated |
| VWA2 | 340706 | 4.44766 | 4.69608 | 2.19E-16 | 7.76E-15 | up-regulated |
| GALNT6 | 11226 | 2.35329 | 6.45011 | 2.20E-16 | 7.81E-15 | up-regulated |
| PRKAR2B | 5577 | -2.3134 | 3.35273 | 2.32E-16 | 8.18E-15 | down-regulated |
| CPNE7 | 27132 | 6.13115 | 4.50534 | 2.49E-16 | 8.77E-15 | up-regulated |
| C5orf40 | 408263 | -3.0315 | -2.5913 | 2.51E-16 | 8.82E-15 | down-regulated |
| TCF7 | 6932 | 2.3192 | 6.16089 | 2.56E-16 | 8.97E-15 | up-regulated |
| MT1F | 4494 | -2.7622 | 3.80817 | 2.77E-16 | 9.69E-15 | down-regulated |
| TMEM132B | 114795 | -2.5679 | -0.9383 | 3.02E-16 | 1.05E-14 | down-regulated |
| HEMGN | 55363 | -3.9665 | -3.8727 | 3.03E-16 | 1.05E-14 | down-regulated |
| CLIP3 | 25999 | -2.2484 | 4.02904 | 3.19E-16 | 1.10E-14 | down-regulated |
| SETBP1 | 26040 | -2.2858 | 3.07118 | 3.38E-16 | 1.17E-14 | down-regulated |
| TCEAL2 | 140597 | -3.9755 | 0.81416 | 3.47E-16 | 1.20E-14 | down-regulated |
| EPHX4 | 253152 | 4.3206 | 3.04865 | 3.50E-16 | 1.21E-14 | up-regulated |
| PYY | 5697 | -5.9098 | 4.04476 | 3.58E-16 | 1.23E-14 | down-regulated |
| OSBPL3 | 26031 | 2.1192 | 5.68552 | 3.68E-16 | 1.26E-14 | up-regulated |
| PRPH | 5630 | -3.7958 | 1.35554 | 3.74E-16 | 1.28E-14 | down-regulated |
| TMEM108 | 66000 | -2.1093 | -0.6188 | 3.76E-16 | 1.29E-14 | down-regulated |
| CCL14 | 6358 | -2.8405 | 2.52216 | 4.06E-16 | 1.39E-14 | down-regulated |
| TOMM34 | 10953 | 2.17661 | 6.88442 | 4.27E-16 | 1.45E-14 | up-regulated |
| GPR119 | 139760 | -3.8608 | -3.6933 | 4.28E-16 | 1.45E-14 | down-regulated |
| INSL5 | 10022 | -6.1075 | 4.08797 | 4.31E-16 | 1.46E-14 | down-regulated |
| NR3C1 | 2908 | -2.0146 | 4.48349 | 5.17E-16 | 1.75E-14 | down-regulated |
| TMEM74 | 157753 | -2.9281 | -1.6694 | 5.27E-16 | 1.78E-14 | down-regulated |
| CDKN2B | 1030 | -2.2457 | 4.76639 | 5.41E-16 | 1.82E-14 | down-regulated |
| JAM2 | 58494 | -2.5155 | 2.02316 | 5.47E-16 | 1.84E-14 | down-regulated |
| PTCHD1 | 139411 | -3.7772 | 0.05146 | 5.52E-16 | 1.86E-14 | down-regulated |
| JPH2 | 57158 | -3.1591 | 3.63414 | 5.56E-16 | 1.86E-14 | down-regulated |
| ARL4D | 379 | -2.4857 | 2.24676 | 5.67E-16 | 1.90E-14 | down-regulated |
| RGMA | 56963 | -2.6508 | 3.5084 | 6.06E-16 | 2.02E-14 | down-regulated |
| PCDH9 | 5101 | -2.9223 | -0.5324 | 7.02E-16 | 2.33E-14 | down-regulated |
| CBLN2 | 147381 | -3.1144 | -0.6095 | 7.22E-16 | 2.40E-14 | down-regulated |
| EFHC2 | 80258 | -3.2 | -1.199 | 7.26E-16 | 2.41E-14 | down-regulated |
| CPM | 1368 | -2.47 | 4.81539 | 7.33E-16 | 2.43E-14 | down-regulated |
| VPREB3 | 29802 | -3.3598 | -0.0924 | 7.60E-16 | 2.50E-14 | down-regulated |
| COL21A1 | 81578 | -2.7775 | 0.70687 | 7.65E-16 | 2.51E-14 | down-regulated |
| UST | 10090 | -2.5338 | 1.67323 | 8.13E-16 | 2.66E-14 | down-regulated |
| FABP6 | 2172 | 7.56761 | 3.03452 | 8.18E-16 | 2.68E-14 | up-regulated |
| RIC3 | 79608 | -3.377 | -0.6706 | 8.47E-16 | 2.76E-14 | down-regulated |
| CDON | 50937 | -2.0809 | 3.1777 | 8.65E-16 | 2.82E-14 | down-regulated |
| CD79B | 974 | -2.7123 | 2.02169 | 8.87E-16 | 2.88E-14 | down-regulated |
| ATRNL1 | 26033 | -2.9695 | -1.0578 | 9.45E-16 | 3.06E-14 | down-regulated |
| C1orf173 | 127254 | -3.6508 | -3.0819 | 9.67E-16 | 3.12E-14 | down-regulated |
| ROPN1 | 54763 | -3.3741 | -2.536 | 1.00E-15 | 3.23E-14 | down-regulated |
| CA1 | 759 | -5.9122 | 7.44736 | 1.01E-15 | 3.24E-14 | down-regulated |
| RNF43 | 54894 | 2.55802 | 8.32777 | 1.01E-15 | 3.24E-14 | up-regulated |
| WDR17 | 116966 | -3.2575 | -0.8748 | 1.05E-15 | 3.38E-14 | down-regulated |
| LOC283856 | 283856 | -2.9814 | -3.0144 | 1.06E-15 | 3.40E-14 | down-regulated |
| INA | 9118 | -3.3163 | -0.2302 | 1.07E-15 | 3.41E-14 | down-regulated |
| LOC100128164 | 100128164 | -2.7545 | -3.2406 | 1.13E-15 | 3.60E-14 | down-regulated |
| CHP2 | 63928 | -3.2941 | 5.87826 | 1.21E-15 | 3.81E-14 | down-regulated |
| CRYBA2 | 1412 | -3.7682 | -1.3306 | 1.29E-15 | 4.08E-14 | down-regulated |
| MAPK4 | 5596 | -3.873 | 0.2441 | 1.36E-15 | 4.28E-14 | down-regulated |
| CLIP4 | 79745 | -2.5535 | 2.69676 | 1.39E-15 | 4.35E-14 | down-regulated |
| C8orf85 | 441376 | -2.7926 | -1.5619 | 1.52E-15 | 4.74E-14 | down-regulated |
| NBEA | 26960 | -2.8915 | 2.25222 | 1.58E-15 | 4.91E-14 | down-regulated |
| HAPLN1 | 1404 | -2.6539 | 2.21484 | 1.91E-15 | 5.91E-14 | down-regulated |
| ELAVL3 | 1995 | -3.7352 | -2.7001 | 2.13E-15 | 6.56E-14 | down-regulated |
| CEND1 | 51286 | -2.3899 | -1.2164 | 2.60E-15 | 7.95E-14 | down-regulated |
| RDH5 | 5959 | -2.0925 | 2.66472 | 2.65E-15 | 8.07E-14 | down-regulated |
| PLD5 | 200150 | -4.3609 | -3.371 | 2.83E-15 | 8.58E-14 | down-regulated |
| EDIL3 | 10085 | -2.3042 | 3.98479 | 2.94E-15 | 8.92E-14 | down-regulated |
| SEC14L5 | 9717 | -2.6768 | -0.5175 | 3.01E-15 | 9.12E-14 | down-regulated |
| C9orf4 | 23732 | -3.8949 | -3.6292 | 3.14E-15 | 9.50E-14 | down-regulated |
| ANLN | 54443 | 2.10536 | 6.09545 | 3.17E-15 | 9.56E-14 | up-regulated |
| CEP72 | 55722 | 2.33394 | 3.13066 | 3.30E-15 | 9.95E-14 | up-regulated |
| PDE7B | 27115 | -2.3608 | 1.21117 | 3.47E-15 | 1.04E-13 | down-regulated |
| CD48 | 962 | -2.2585 | 3.28066 | 3.55E-15 | 1.06E-13 | down-regulated |
| AFF2 | 2334 | -3.0933 | -1.0558 | 3.64E-15 | 1.09E-13 | down-regulated |
| CTNND2 | 1501 | -3.5424 | -1.2036 | 3.72E-15 | 1.11E-13 | down-regulated |
| TMEM59L | 25789 | -3.0458 | -0.4469 | 3.73E-15 | 1.11E-13 | down-regulated |
| SLC4A10 | 57282 | -3.1331 | -0.7591 | 4.28E-15 | 1.27E-13 | down-regulated |
| FAM189A2 | 9413 | -2.9428 | 0.14877 | 4.36E-15 | 1.29E-13 | down-regulated |
| MUSTN1 | 389125 | -2.6569 | 0.38006 | 4.51E-15 | 1.33E-13 | down-regulated |
| CCDC13 | 152206 | -2.0845 | -1.566 | 4.58E-15 | 1.35E-13 | down-regulated |
| IGJ | 3512 | -3.8056 | 9.68813 | 4.69E-15 | 1.38E-13 | down-regulated |
| PITX2 | 5308 | 4.72813 | 3.67848 | 4.92E-15 | 1.44E-13 | up-regulated |
| TRPV3 | 162514 | -3.1151 | -0.5838 | 4.99E-15 | 1.46E-13 | down-regulated |
| SCN4B | 6330 | -2.242 | 1.6185 | 5.12E-15 | 1.50E-13 | down-regulated |
| C20orf194 | 25943 | -2.3329 | 2.91893 | 5.14E-15 | 1.50E-13 | down-regulated |
| GDNF | 2668 | -2.6451 | -2.2023 | 6.11E-15 | 1.77E-13 | down-regulated |
| C1QTNF7 | 114905 | -2.8356 | 1.38322 | 6.14E-15 | 1.78E-13 | down-regulated |
| LYNX1 | 66004 | -2.6443 | 2.44903 | 6.31E-15 | 1.82E-13 | down-regulated |
| COL19A1 | 1310 | -3.0813 | -2.1354 | 6.37E-15 | 1.84E-13 | down-regulated |
| C11orf86 | 254439 | -3.6906 | 2.51777 | 6.49E-15 | 1.87E-13 | down-regulated |
| WSCD1 | 23302 | -2.5168 | 3.17904 | 6.76E-15 | 1.94E-13 | down-regulated |
| POU5F1B | 5462 | 4.89314 | 3.31478 | 6.76E-15 | 1.94E-13 | up-regulated |
| HHLA2 | 11148 | -2.4398 | 5.80417 | 7.74E-15 | 2.21E-13 | down-regulated |
| SFRP1 | 6422 | -3.6932 | 3.71476 | 7.76E-15 | 2.21E-13 | down-regulated |
| DVWA | 344875 | 5.82103 | -2.4103 | 7.92E-15 | 2.25E-13 | up-regulated |
| EFHA2 | 286097 | -2.5157 | 0.21225 | 7.99E-15 | 2.27E-13 | down-regulated |
| CD22 | 933 | -3.1723 | 2.02866 | 8.01E-15 | 2.27E-13 | down-regulated |
| LOC728264 | 728264 | -2.56 | 3.93886 | 8.03E-15 | 2.27E-13 | down-regulated |
| P2RY1 | 5028 | -2.5033 | 1.83896 | 8.09E-15 | 2.29E-13 | down-regulated |
| GDPD2 | 54857 | -2.9712 | 1.74465 | 8.78E-15 | 2.48E-13 | down-regulated |
| NOVA1 | 4857 | -3.1479 | 0.58782 | 8.83E-15 | 2.49E-13 | down-regulated |
| ST8SIA1 | 6489 | -2.4158 | 1.14472 | 8.99E-15 | 2.53E-13 | down-regulated |
| EPHA5 | 2044 | -3.0355 | -2.5276 | 9.26E-15 | 2.60E-13 | down-regulated |
| LRMP | 4033 | -2.7383 | 1.98273 | 9.32E-15 | 2.61E-13 | down-regulated |
| SLC17A4 | 10050 | -2.8542 | 2.91701 | 9.64E-15 | 2.69E-13 | down-regulated |
| LOC144571 | 144571 | -2.1197 | -0.1524 | 9.96E-15 | 2.77E-13 | down-regulated |
| RASGRP2 | 10235 | -2.2809 | 1.66465 | 1.03E-14 | 2.86E-13 | down-regulated |
| ABCC8 | 6833 | -3.2338 | -2.3718 | 1.04E-14 | 2.89E-13 | down-regulated |
| PLEKHG4 | 25894 | 2.46685 | 5.18422 | 1.05E-14 | 2.90E-13 | up-regulated |
| TPX2 | 22974 | 2.10187 | 7.40339 | 1.06E-14 | 2.94E-13 | up-regulated |
| VSNL1 | 7447 | 3.76763 | 4.97164 | 1.06E-14 | 2.94E-13 | up-regulated |
| FCRLA | 84824 | -3.4685 | 0.64791 | 1.39E-14 | 3.81E-13 | down-regulated |
| P2RY12 | 64805 | -3.1115 | -1.1222 | 1.58E-14 | 4.31E-13 | down-regulated |
| MASP1 | 5648 | -3.5095 | 3.50749 | 1.71E-14 | 4.65E-13 | down-regulated |
| GUCA2B | 2981 | -5.156 | 2.96243 | 1.72E-14 | 4.68E-13 | down-regulated |
| MFSD4 | 148808 | -2.5958 | 4.71844 | 2.00E-14 | 5.41E-13 | down-regulated |
| SCN3A | 6328 | -2.3943 | -0.1817 | 2.01E-14 | 5.43E-13 | down-regulated |
| C7orf68 | 29923 | 2.28593 | 5.08243 | 2.04E-14 | 5.49E-13 | up-regulated |
| MT1E | 4493 | -2.9282 | 5.6903 | 2.06E-14 | 5.52E-13 | down-regulated |
| SNCG | 6623 | -2.407 | 1.24324 | 2.11E-14 | 5.66E-13 | down-regulated |
| HSD17B2 | 3294 | -2.7776 | 4.34578 | 2.55E-14 | 6.80E-13 | down-regulated |
| SYT10 | 341359 | -3.9339 | -2.9503 | 2.56E-14 | 6.82E-13 | down-regulated |
| SLC27A6 | 28965 | -3.3094 | -1.6959 | 2.57E-14 | 6.82E-13 | down-regulated |
| MPP2 | 4355 | -2.3238 | 1.29594 | 2.61E-14 | 6.92E-13 | down-regulated |
| CARD14 | 79092 | 3.68138 | 2.01863 | 2.61E-14 | 6.92E-13 | up-regulated |
| C6orf176 | 90632 | -3.4018 | -1.5033 | 2.64E-14 | 6.99E-13 | down-regulated |
| LPHN3 | 23284 | -2.4714 | 1.27417 | 2.65E-14 | 7.00E-13 | down-regulated |
| USP2 | 9099 | -2.547 | 2.21995 | 2.81E-14 | 7.42E-13 | down-regulated |
| STAC | 6769 | -3.1759 | 0.17353 | 3.26E-14 | 8.54E-13 | down-regulated |
| CD200R1 | 131450 | -2.446 | -0.3452 | 3.41E-14 | 8.91E-13 | down-regulated |
| CACNA2D1 | 781 | -3.1118 | 0.98031 | 3.75E-14 | 9.74E-13 | down-regulated |
| PLAC9 | 219348 | -3.0951 | 1.10507 | 3.82E-14 | 9.91E-13 | down-regulated |
| FOLR2 | 2350 | -2.7371 | 2.98118 | 3.96E-14 | 1.02E-12 | down-regulated |
| MEIS1 | 4211 | -2.2941 | 3.4051 | 4.20E-14 | 1.08E-12 | down-regulated |
| AMPD1 | 270 | -3.8809 | -0.6038 | 4.29E-14 | 1.10E-12 | down-regulated |
| LOC100190940 | 100190940 | 6.97697 | 4.09028 | 4.47E-14 | 1.15E-12 | up-regulated |
| MAPK10 | 5602 | -2.3675 | 1.05954 | 4.50E-14 | 1.15E-12 | down-regulated |
| TMTC1 | 83857 | -2.0761 | 3.28008 | 4.54E-14 | 1.16E-12 | down-regulated |
| IGFBP6 | 3489 | -2.291 | 3.6018 | 4.60E-14 | 1.18E-12 | down-regulated |
| PDE9A | 5152 | -2.3269 | 5.05965 | 4.74E-14 | 1.21E-12 | down-regulated |
| FERMT2 | 10979 | -2.0949 | 5.2339 | 4.90E-14 | 1.25E-12 | down-regulated |
| ITM2C | 81618 | -2.1066 | 9.47635 | 5.00E-14 | 1.27E-12 | down-regulated |
| SPARCL1 | 8404 | -2.3269 | 7.18856 | 5.09E-14 | 1.29E-12 | down-regulated |
| FAM135B | 51059 | -3.908 | -0.8784 | 5.11E-14 | 1.29E-12 | down-regulated |
| AOC3 | 8639 | -2.5415 | 5.80275 | 5.55E-14 | 1.40E-12 | down-regulated |
| LMTK3 | 114783 | 3.75923 | 2.72927 | 5.57E-14 | 1.40E-12 | up-regulated |
| LOC643763 | 643763 | -3.564 | -0.9719 | 5.60E-14 | 1.41E-12 | down-regulated |
| XRCC2 | 7516 | 2.38609 | 2.66501 | 6.20E-14 | 1.55E-12 | up-regulated |
| ATG9B | 285973 | 3.97771 | 2.40439 | 6.33E-14 | 1.58E-12 | up-regulated |
| MT1G | 4495 | -3.1044 | 5.95692 | 6.37E-14 | 1.59E-12 | down-regulated |
| RERG | 85004 | -2.5177 | 2.80049 | 6.50E-14 | 1.62E-12 | down-regulated |
| SLC5A6 | 8884 | 2.08326 | 7.01316 | 6.71E-14 | 1.67E-12 | up-regulated |
| NCRNA00092 | 100188953 | -2.6226 | -1.7212 | 6.82E-14 | 1.69E-12 | down-regulated |
| METTL7A | 25840 | -2.0395 | 6.03394 | 6.85E-14 | 1.70E-12 | down-regulated |
| GRHL3 | 57822 | 5.76931 | 2.48994 | 7.81E-14 | 1.92E-12 | up-regulated |
| GGTA1 | 2681 | -2.3766 | 1.90145 | 8.44E-14 | 2.07E-12 | down-regulated |
| KIAA1683 | 80726 | -2.05 | 0.93936 | 8.89E-14 | 2.17E-12 | down-regulated |
| C1orf133 | 574036 | -2.4052 | 0.31325 | 9.07E-14 | 2.21E-12 | down-regulated |
| DBNDD1 | 79007 | 2.49364 | 4.60461 | 9.45E-14 | 2.30E-12 | up-regulated |
| MAP6 | 4135 | -2.3957 | 1.31489 | 9.66E-14 | 2.35E-12 | down-regulated |
| AMOTL1 | 154810 | -2.2053 | 4.93752 | 9.75E-14 | 2.36E-12 | down-regulated |
| ZNF536 | 9745 | -3.0941 | -0.5501 | 1.00E-13 | 2.42E-12 | down-regulated |
| GPRASP1 | 9737 | -2.0622 | 2.60232 | 1.15E-13 | 2.77E-12 | down-regulated |
| SEZ6 | 124925 | -3.4752 | -2.4082 | 1.18E-13 | 2.84E-12 | down-regulated |
| MGAT4C | 25834 | -3.7909 | -3.3836 | 1.24E-13 | 2.98E-12 | down-regulated |
| CA12 | 771 | -2.095 | 7.13135 | 1.28E-13 | 3.06E-12 | down-regulated |
| LOC388796 | 388796 | 2.41417 | 5.54146 | 1.30E-13 | 3.10E-12 | up-regulated |
| MEIS2 | 4212 | -2.5401 | 2.64131 | 1.33E-13 | 3.18E-12 | down-regulated |
| SFTA1P | 207107 | -3.1051 | -2.5326 | 1.35E-13 | 3.23E-12 | down-regulated |
| CDH10 | 1008 | -3.9913 | -3.4409 | 1.41E-13 | 3.35E-12 | down-regulated |
| TMEM132A | 54972 | 2.30381 | 5.2273 | 1.45E-13 | 3.46E-12 | up-regulated |
| FLJ43390 | 646113 | -2.9003 | -3.548 | 1.49E-13 | 3.53E-12 | down-regulated |
| STRA6 | 64220 | 6.68387 | 3.9852 | 1.52E-13 | 3.59E-12 | up-regulated |
| FAM13C | 220965 | -2.2997 | 1.58051 | 1.58E-13 | 3.74E-12 | down-regulated |
| LRRC19 | 64922 | -2.5222 | 5.11477 | 1.60E-13 | 3.77E-12 | down-regulated |
| EPHA7 | 2045 | -3.4707 | 2.65905 | 1.62E-13 | 3.80E-12 | down-regulated |
| KCNC1 | 3746 | -2.5461 | -2.3635 | 1.63E-13 | 3.83E-12 | down-regulated |
| SNCA | 6622 | -2.5656 | 1.22502 | 1.65E-13 | 3.86E-12 | down-regulated |
| MAGEE2 | 139599 | -3.1453 | -3.4021 | 1.77E-13 | 4.14E-12 | down-regulated |
| CHGA | 1113 | -4.4086 | 5.45237 | 1.78E-13 | 4.16E-12 | down-regulated |
| HBB | 3043 | -2.9348 | 4.55265 | 1.82E-13 | 4.24E-12 | down-regulated |
| ROR1 | 4919 | -2.7163 | 0.91213 | 1.83E-13 | 4.25E-12 | down-regulated |
| AQPEP | 206338 | -3.0986 | -1.7164 | 1.83E-13 | 4.25E-12 | down-regulated |
| MMP7 | 4316 | 7.25865 | 5.92434 | 1.97E-13 | 4.58E-12 | up-regulated |
| CAMK2A | 815 | -2.5167 | -1.5113 | 2.22E-13 | 5.14E-12 | down-regulated |
| LRFN5 | 145581 | -2.8137 | -0.7116 | 2.26E-13 | 5.21E-12 | down-regulated |
| LDHD | 197257 | -2.5868 | 3.64628 | 2.43E-13 | 5.59E-12 | down-regulated |
| SLC7A5 | 8140 | 2.71023 | 7.5646 | 2.59E-13 | 5.94E-12 | up-regulated |
| ABCC13 | 150000 | -3.2556 | 0.47104 | 2.61E-13 | 5.98E-12 | down-regulated |
| LY6G6E | 79136 | 7.88561 | -0.6191 | 2.70E-13 | 6.18E-12 | up-regulated |
| REEP2 | 51308 | -2.4824 | 1.31092 | 2.77E-13 | 6.32E-12 | down-regulated |
| SEMA3E | 9723 | -3.8979 | 0.60612 | 2.89E-13 | 6.59E-12 | down-regulated |
| HSPB2 | 3316 | -2.0026 | 1.38305 | 2.90E-13 | 6.60E-12 | down-regulated |
| NTRK3 | 4916 | -2.96 | -1.0585 | 3.17E-13 | 7.19E-12 | down-regulated |
| CELF4 | 56853 | -2.835 | -1.854 | 3.24E-13 | 7.33E-12 | down-regulated |
| KRT23 | 25984 | 8.80478 | 7.16795 | 3.41E-13 | 7.69E-12 | up-regulated |
| TACR1 | 6869 | -2.8265 | -0.8206 | 3.42E-13 | 7.70E-12 | down-regulated |
| INHBA | 3624 | 4.98065 | 3.80544 | 3.46E-13 | 7.79E-12 | up-regulated |
| RNF180 | 285671 | -2.1958 | 1.04299 | 3.52E-13 | 7.91E-12 | down-regulated |
| ASB5 | 140458 | -4.947 | 0.98592 | 3.61E-13 | 8.10E-12 | down-regulated |
| ADCY5 | 111 | -2.9069 | 2.27581 | 4.05E-13 | 9.06E-12 | down-regulated |
| LRP8 | 7804 | 2.63406 | 4.13193 | 4.35E-13 | 9.69E-12 | up-regulated |
| DDN | 23109 | 4.05507 | 1.85264 | 4.35E-13 | 9.69E-12 | up-regulated |
| TOP1MT | 116447 | 2.12059 | 6.34545 | 4.36E-13 | 9.70E-12 | up-regulated |
| NACAD | 23148 | -2.2047 | 1.31887 | 4.58E-13 | 1.02E-11 | down-regulated |
| SLC17A7 | 57030 | -2.7939 | -0.8543 | 5.11E-13 | 1.13E-11 | down-regulated |
| FAM163A | 148753 | -2.2136 | -0.5761 | 5.26E-13 | 1.16E-11 | down-regulated |
| SLC22A18AS | 5003 | -2.0374 | 3.01229 | 5.45E-13 | 1.20E-11 | down-regulated |
| PNCK | 139728 | -3.6594 | 2.02854 | 5.48E-13 | 1.21E-11 | down-regulated |
| FAM23A | 653567 | -2.788 | 4.20287 | 5.67E-13 | 1.25E-11 | down-regulated |
| LOC100128239 | 100128239 | -2.4544 | -1.5792 | 5.71E-13 | 1.25E-11 | down-regulated |
| GAP43 | 2596 | -2.9855 | 0.49157 | 5.95E-13 | 1.30E-11 | down-regulated |
| ADAMTSL1 | 92949 | -2.1254 | 2.3696 | 6.00E-13 | 1.32E-11 | down-regulated |
| C12orf53 | 196500 | -2.3636 | -0.1647 | 6.08E-13 | 1.33E-11 | down-regulated |
| PPY | 5539 | -5.3592 | -2.2048 | 6.10E-13 | 1.33E-11 | down-regulated |
| TCF21 | 6943 | -2.0281 | 3.30829 | 6.15E-13 | 1.34E-11 | down-regulated |
| CD36 | 948 | -2.5743 | 3.74609 | 6.25E-13 | 1.36E-11 | down-regulated |
| FOXP2 | 93986 | -2.9883 | 1.92646 | 6.26E-13 | 1.36E-11 | down-regulated |
| ANKRD53 | 79998 | -2.3381 | -1.9975 | 6.27E-13 | 1.36E-11 | down-regulated |
| FAM181B | 220382 | -2.8555 | -2.0957 | 6.30E-13 | 1.37E-11 | down-regulated |
| SKA3 | 221150 | 2.29814 | 5.01482 | 6.52E-13 | 1.41E-11 | up-regulated |
| FCER2 | 2208 | -3.8088 | -0.4118 | 6.69E-13 | 1.44E-11 | down-regulated |
| PLCL2 | 23228 | -2.1767 | 3.13835 | 7.24E-13 | 1.56E-11 | down-regulated |
| PLEKHN1 | 84069 | 3.72116 | 1.7567 | 7.81E-13 | 1.68E-11 | up-regulated |
| C2orf74 | 339804 | -2.2295 | 0.89615 | 7.81E-13 | 1.68E-11 | down-regulated |
| NRSN1 | 140767 | -3.6961 | -2.1123 | 8.62E-13 | 1.83E-11 | down-regulated |
| AKR1B10 | 57016 | -3.1022 | 4.39699 | 8.64E-13 | 1.83E-11 | down-regulated |
| CNKSR2 | 22866 | -2.9435 | -2.7721 | 8.81E-13 | 1.87E-11 | down-regulated |
| MMP11 | 4320 | 4.85886 | 7.49808 | 8.83E-13 | 1.87E-11 | up-regulated |
| GABRD | 2563 | 3.19724 | 0.67507 | 8.88E-13 | 1.88E-11 | up-regulated |
| TMOD1 | 7111 | -2.211 | 3.24362 | 9.41E-13 | 1.98E-11 | down-regulated |
| CXCR5 | 643 | -2.9965 | 0.63105 | 9.67E-13 | 2.03E-11 | down-regulated |
| TNFRSF13B | 23495 | -3.0897 | -0.6551 | 9.76E-13 | 2.05E-11 | down-regulated |
| KCNK3 | 3777 | -2.8831 | 0.18735 | 9.86E-13 | 2.07E-11 | down-regulated |
| C2 | 717 | 2.63867 | 7.21883 | 9.88E-13 | 2.07E-11 | up-regulated |
| FGF2 | 2247 | -2.1266 | 2.78142 | 1.01E-12 | 2.10E-11 | down-regulated |
| COL11A1 | 1301 | 7.09264 | 5.55515 | 1.06E-12 | 2.20E-11 | up-regulated |
| SNTG2 | 54221 | -3.1474 | -3.7421 | 1.09E-12 | 2.27E-11 | down-regulated |
| B3GNT7 | 93010 | -3.2847 | 7.0267 | 1.19E-12 | 2.45E-11 | down-regulated |
| UGT1A8 | 54576 | -3.4775 | 1.97305 | 1.26E-12 | 2.59E-11 | down-regulated |
| SPINK2 | 6691 | -3.3528 | -2.029 | 1.28E-12 | 2.64E-11 | down-regulated |
| ITGA7 | 3679 | -2.1941 | 4.21892 | 1.33E-12 | 2.74E-11 | down-regulated |
| MAPT | 4137 | -2.918 | 1.45099 | 1.39E-12 | 2.86E-11 | down-regulated |
| SYT6 | 148281 | -2.9214 | -2.922 | 1.45E-12 | 2.98E-11 | down-regulated |
| ADRA1D | 146 | -2.6129 | -0.9122 | 1.50E-12 | 3.06E-11 | down-regulated |
| TSPAN1 | 10103 | -2.2616 | 8.70349 | 1.52E-12 | 3.10E-11 | down-regulated |
| SERTAD4 | 56256 | -2.3414 | 1.02773 | 1.55E-12 | 3.15E-11 | down-regulated |
| DNASE1L3 | 1776 | -3.3551 | 2.63179 | 1.64E-12 | 3.31E-11 | down-regulated |
| FLRT1 | 23769 | -2.1922 | 0.39661 | 1.69E-12 | 3.43E-11 | down-regulated |
| ZBTB16 | 7704 | -3.1063 | -0.0253 | 1.80E-12 | 3.62E-11 | down-regulated |
| HSPH1 | 10808 | 2.35919 | 8.3409 | 1.80E-12 | 3.62E-11 | up-regulated |
| PCDH11X | 27328 | -3.7118 | -3.0666 | 1.80E-12 | 3.63E-11 | down-regulated |
| NLGN4X | 57502 | -2.1618 | 1.21261 | 1.95E-12 | 3.93E-11 | down-regulated |
| CNTD2 | 79935 | 4.84215 | 1.34244 | 1.98E-12 | 3.97E-11 | up-regulated |
| ANKRD35 | 148741 | -2.1855 | 0.45299 | 2.02E-12 | 4.05E-11 | down-regulated |
| SCD | 6319 | 2.3839 | 9.26738 | 2.05E-12 | 4.09E-11 | up-regulated |
| THRB | 7068 | -2.2482 | 3.94929 | 2.07E-12 | 4.14E-11 | down-regulated |
| CDKL2 | 8999 | -2.362 | -0.0962 | 2.13E-12 | 4.24E-11 | down-regulated |
| WBSCR17 | 64409 | -2.5366 | 0.9884 | 2.13E-12 | 4.24E-11 | down-regulated |
| ANO5 | 203859 | -3.1056 | 2.01527 | 2.17E-12 | 4.31E-11 | down-regulated |
| C1orf150 | 148823 | -2.8689 | -1.9315 | 2.18E-12 | 4.33E-11 | down-regulated |
| KCNE2 | 9992 | -2.1505 | -2.2832 | 2.28E-12 | 4.50E-11 | down-regulated |
| TLX1 | 3195 | 4.76128 | 1.31528 | 2.30E-12 | 4.54E-11 | up-regulated |
| APLN | 8862 | 3.60184 | 3.50095 | 2.44E-12 | 4.79E-11 | up-regulated |
| SALL2 | 6297 | -2.1413 | 1.83324 | 2.49E-12 | 4.88E-11 | down-regulated |
| ERVFRDE1 | 405754 | -2.9016 | -2.7578 | 2.63E-12 | 5.14E-11 | down-regulated |
| UNC5C | 8633 | -2.5561 | 0.1291 | 2.63E-12 | 5.15E-11 | down-regulated |
| BEND4 | 389206 | -2.6908 | -1.6508 | 2.68E-12 | 5.23E-11 | down-regulated |
| GPR183 | 1880 | -2.0272 | 3.20672 | 2.70E-12 | 5.27E-11 | down-regulated |
| TNFRSF17 | 608 | -3.2293 | 1.40059 | 2.76E-12 | 5.37E-11 | down-regulated |
| SMOX | 54498 | 2.28009 | 4.80418 | 2.82E-12 | 5.48E-11 | up-regulated |
| IFITM1 | 8519 | 2.57735 | 8.77689 | 2.82E-12 | 5.49E-11 | up-regulated |
| TLR10 | 81793 | -2.8472 | 0.24913 | 2.85E-12 | 5.53E-11 | down-regulated |
| C1orf186 | 440712 | -2.7829 | -1.5202 | 2.92E-12 | 5.65E-11 | down-regulated |
| PRDM6 | 93166 | -2.7106 | 0.4984 | 2.93E-12 | 5.67E-11 | down-regulated |
| PCOLCE2 | 26577 | -3.4976 | 1.77575 | 2.98E-12 | 5.75E-11 | down-regulated |
| CMA1 | 1215 | -4.5147 | -1.2105 | 3.01E-12 | 5.78E-11 | down-regulated |
| SLIT3 | 6586 | -2.3928 | 4.09935 | 3.20E-12 | 6.13E-11 | down-regulated |
| TMIGD1 | 388364 | -5.2396 | 3.6534 | 3.29E-12 | 6.27E-11 | down-regulated |
| SEPP1 | 6414 | -2.3585 | 8.95474 | 3.34E-12 | 6.36E-11 | down-regulated |
| PABPC1L | 80336 | 3.95521 | 4.5495 | 3.46E-12 | 6.58E-11 | up-regulated |
| ADHFE1 | 137872 | -2.7813 | -0.1952 | 3.54E-12 | 6.71E-11 | down-regulated |
| CLECL1 | 160365 | -2.414 | -1.6005 | 3.63E-12 | 6.86E-11 | down-regulated |
| PDLIM3 | 27295 | -2.2776 | 5.32097 | 3.65E-12 | 6.89E-11 | down-regulated |
| IL1R2 | 7850 | -2.8022 | 3.724 | 3.94E-12 | 7.39E-11 | down-regulated |
| COL7A1 | 1294 | 4.77816 | 5.11402 | 3.95E-12 | 7.41E-11 | up-regulated |
| CEACAM3 | 1084 | -2.5999 | -0.9636 | 4.20E-12 | 7.86E-11 | down-regulated |
| CALD1 | 800 | -2.0184 | 8.45408 | 4.28E-12 | 7.99E-11 | down-regulated |
| GPR22 | 2845 | -2.6879 | -3.4791 | 4.67E-12 | 8.66E-11 | down-regulated |
| CDC25B | 994 | 2.42919 | 7.4358 | 4.86E-12 | 8.99E-11 | up-regulated |
| SHISA3 | 152573 | -2.9118 | 1.49845 | 4.86E-12 | 8.99E-11 | down-regulated |
| C6orf155 | 79940 | -2.257 | -2.8808 | 4.91E-12 | 9.07E-11 | down-regulated |
| SEMA6D | 80031 | -2.1508 | 3.49611 | 5.18E-12 | 9.55E-11 | down-regulated |
| BLK | 640 | -3.3042 | 0.00658 | 5.21E-12 | 9.59E-11 | down-regulated |
| KIRREL3 | 84623 | -2.6874 | -0.8645 | 5.37E-12 | 9.84E-11 | down-regulated |
| GDPD5 | 81544 | 3.33224 | 5.42245 | 5.76E-12 | 1.05E-10 | up-regulated |
| CHRNB4 | 1143 | -2.5945 | -1.5653 | 5.97E-12 | 1.09E-10 | down-regulated |
| MIR17HG | 407975 | 3.18202 | 2.78904 | 6.13E-12 | 1.12E-10 | up-regulated |
| CST1 | 1469 | 8.29708 | 5.72166 | 6.17E-12 | 1.12E-10 | up-regulated |
| HLF | 3131 | -2.5361 | 2.37594 | 6.90E-12 | 1.25E-10 | down-regulated |
| GPR156 | 165829 | -2.6959 | -2.9494 | 6.91E-12 | 1.25E-10 | down-regulated |
| NPAS3 | 64067 | -2.3173 | -0.9913 | 6.98E-12 | 1.26E-10 | down-regulated |
| JAKMIP3 | 282973 | -2.1442 | -1.6205 | 7.11E-12 | 1.28E-10 | down-regulated |
| RUNDC3B | 154661 | -2.1183 | 1.2858 | 7.18E-12 | 1.29E-10 | down-regulated |
| FAM46C | 54855 | -2.0507 | 4.88202 | 7.38E-12 | 1.32E-10 | down-regulated |
| FAM176A | 84141 | 4.16095 | 3.21227 | 7.44E-12 | 1.33E-10 | up-regulated |
| LOC339524 | 339524 | -2.3719 | 0.31587 | 7.80E-12 | 1.39E-10 | down-regulated |
| BRCA2 | 675 | 2.26054 | 3.90393 | 7.99E-12 | 1.42E-10 | up-regulated |
| STON1 | 11037 | -2.2293 | 3.85625 | 8.01E-12 | 1.42E-10 | down-regulated |
| LGI4 | 163175 | -2.2297 | 2.43884 | 8.44E-12 | 1.50E-10 | down-regulated |
| KLHL35 | 283212 | 3.17742 | 1.5122 | 8.71E-12 | 1.54E-10 | up-regulated |
| VIPR2 | 7434 | -2.5075 | 0.83501 | 8.95E-12 | 1.58E-10 | down-regulated |
| ANKRD13B | 124930 | 2.26411 | 3.9761 | 9.04E-12 | 1.59E-10 | up-regulated |
| FAM129C | 199786 | -3.2213 | -1.5769 | 9.37E-12 | 1.65E-10 | down-regulated |
| ASAM | 79827 | -2.4288 | 2.98593 | 1.00E-11 | 1.76E-10 | down-regulated |
| GPER | 2852 | -2.4356 | 2.0094 | 1.01E-11 | 1.77E-10 | down-regulated |
| OXTR | 5021 | 2.91096 | 1.47711 | 1.02E-11 | 1.79E-10 | up-regulated |
| PDE1A | 5136 | -2.2479 | 1.9886 | 1.03E-11 | 1.80E-10 | down-regulated |
| DGAT2 | 84649 | 2.08994 | 5.32247 | 1.03E-11 | 1.80E-10 | up-regulated |
| CNTN4 | 152330 | -2.0193 | 1.80084 | 1.06E-11 | 1.85E-10 | down-regulated |
| MT1H | 4496 | -3.3199 | 2.4259 | 1.09E-11 | 1.90E-10 | down-regulated |
| LY6H | 4062 | -2.9837 | -0.5005 | 1.11E-11 | 1.93E-10 | down-regulated |
| CHRDL1 | 91851 | -3.2395 | 4.48277 | 1.13E-11 | 1.96E-10 | down-regulated |
| ORC6L | 23594 | 2.12027 | 4.01282 | 1.14E-11 | 1.98E-10 | up-regulated |
| B3GALT1 | 8708 | -3.3811 | -0.1187 | 1.17E-11 | 2.02E-10 | down-regulated |
| CCR10 | 2826 | -2.2324 | -0.7637 | 1.23E-11 | 2.13E-10 | down-regulated |
| SNTB1 | 6641 | 2.49148 | 6.4328 | 1.26E-11 | 2.18E-10 | up-regulated |
| FXYD1 | 5348 | -3.2009 | 1.27252 | 1.28E-11 | 2.21E-10 | down-regulated |
| KIF1A | 547 | -3.3385 | 1.10608 | 1.28E-11 | 2.21E-10 | down-regulated |
| CACNA1H | 8912 | -2.1783 | 4.89884 | 1.29E-11 | 2.22E-10 | down-regulated |
| AXIN2 | 8313 | 2.69697 | 7.76801 | 1.31E-11 | 2.26E-10 | up-regulated |
| DPF3 | 8110 | -2.2286 | 0.61198 | 1.32E-11 | 2.26E-10 | down-regulated |
| PHOX2A | 401 | -3.4235 | -2.3977 | 1.33E-11 | 2.29E-10 | down-regulated |
| NKAPL | 222698 | -2.2267 | -1.9306 | 1.36E-11 | 2.32E-10 | down-regulated |
| OTX1 | 5013 | 5.14984 | 0.42254 | 1.37E-11 | 2.35E-10 | up-regulated |
| RASGEF1C | 255426 | -3.7027 | -2.1114 | 1.38E-11 | 2.35E-10 | down-regulated |
| SCN4A | 6329 | -2.5706 | -1.2485 | 1.39E-11 | 2.36E-10 | down-regulated |
| SH3GL3 | 6457 | -3.9146 | -3.7694 | 1.46E-11 | 2.47E-10 | down-regulated |
| PRR7 | 80758 | 2.6305 | 2.33582 | 1.47E-11 | 2.48E-10 | up-regulated |
| KLK6 | 5653 | 8.97106 | 4.72832 | 1.58E-11 | 2.66E-10 | up-regulated |
| COL10A1 | 1300 | 7.99646 | 5.04304 | 1.64E-11 | 2.75E-10 | up-regulated |
| KRTAP13-2 | 337959 | -4.7436 | 0.25198 | 1.65E-11 | 2.76E-10 | down-regulated |
| MFAP5 | 8076 | -2.8904 | 4.24686 | 1.74E-11 | 2.89E-10 | down-regulated |
| MGC14436 | 84983 | 4.35195 | -0.1181 | 1.76E-11 | 2.93E-10 | up-regulated |
| PKP1 | 5317 | 4.66541 | 3.75955 | 1.90E-11 | 3.15E-10 | up-regulated |
| AZGP1 | 563 | 3.91058 | 6.62764 | 1.91E-11 | 3.17E-10 | up-regulated |
| CTSG | 1511 | -3.2814 | 0.67687 | 2.17E-11 | 3.59E-10 | down-regulated |
| FXYD5 | 53827 | 2.21694 | 6.93092 | 2.23E-11 | 3.68E-10 | up-regulated |
| LEMD1 | 93273 | 8.34348 | 1.13496 | 2.39E-11 | 3.94E-10 | up-regulated |
| ARID3A | 1820 | 2.74677 | 4.40312 | 2.48E-11 | 4.08E-10 | up-regulated |
| HOXD1 | 3231 | -2.5512 | 0.76926 | 2.58E-11 | 4.22E-10 | down-regulated |
| GPR26 | 2849 | -3.3346 | -3.6288 | 2.59E-11 | 4.24E-10 | down-regulated |
| C2orf70 | 339778 | 3.4832 | 1.44168 | 2.66E-11 | 4.34E-10 | up-regulated |
| KCNQ5 | 56479 | -2.7435 | -1.3706 | 2.66E-11 | 4.34E-10 | down-regulated |
| TMEM220 | 388335 | -2.4934 | 2.03532 | 2.80E-11 | 4.55E-10 | down-regulated |
| ARHGAP20 | 57569 | -2.2909 | 0.5235 | 3.02E-11 | 4.89E-10 | down-regulated |
| SEMA6A | 57556 | -2.1056 | 5.06404 | 3.07E-11 | 4.97E-10 | down-regulated |
| CNR2 | 1269 | -2.8914 | -1.8886 | 3.18E-11 | 5.12E-10 | down-regulated |
| TLL1 | 7092 | -2.4145 | 0.05603 | 3.22E-11 | 5.17E-10 | down-regulated |
| CLDN2 | 9075 | 6.84798 | 7.22601 | 3.35E-11 | 5.36E-10 | up-regulated |
| DIRC3 | 729582 | -2.6615 | -2.2502 | 3.40E-11 | 5.44E-10 | down-regulated |
| STOX2 | 56977 | -2.212 | -1.4761 | 3.44E-11 | 5.50E-10 | down-regulated |
| GPR34 | 2857 | -2.0792 | 2.34597 | 3.66E-11 | 5.84E-10 | down-regulated |
| ARPP21 | 10777 | -3.4209 | -3.3378 | 3.76E-11 | 5.99E-10 | down-regulated |
| C1orf70 | 339453 | -2.1485 | -1.4184 | 3.79E-11 | 6.04E-10 | down-regulated |
| GRIN2A | 2903 | -2.9166 | -1.337 | 3.82E-11 | 6.06E-10 | down-regulated |
| FGFR2 | 2263 | -2.3423 | 4.3873 | 3.92E-11 | 6.20E-10 | down-regulated |
| CD19 | 930 | -3.1015 | 0.45308 | 4.07E-11 | 6.43E-10 | down-regulated |
| C6orf223 | 221416 | 3.8842 | 5.37507 | 4.19E-11 | 6.59E-10 | up-regulated |
| FBXO41 | 150726 | 2.1031 | 4.40441 | 4.30E-11 | 6.76E-10 | up-regulated |
| RNF183 | 138065 | 3.7776 | 1.68477 | 4.34E-11 | 6.82E-10 | up-regulated |
| C21orf88 | 114041 | -3.7978 | 1.76087 | 4.38E-11 | 6.87E-10 | down-regulated |
| LOC389791 | 389791 | 4.08203 | 0.17485 | 4.40E-11 | 6.89E-10 | up-regulated |
| ADAMTS1 | 9510 | -2.0286 | 5.31408 | 4.50E-11 | 7.04E-10 | down-regulated |
| AKAP12 | 9590 | -2.1433 | 5.45349 | 4.80E-11 | 7.49E-10 | down-regulated |
| UBE2C | 11065 | 2.1637 | 6.64215 | 4.93E-11 | 7.67E-10 | up-regulated |
| PLIN4 | 729359 | -3.5587 | 4.19025 | 4.94E-11 | 7.69E-10 | down-regulated |
| SNORD1C | 677850 | 2.5433 | 0.73821 | 5.02E-11 | 7.79E-10 | up-regulated |
| SCRG1 | 11341 | -3.8823 | -0.0583 | 5.22E-11 | 8.08E-10 | down-regulated |
| FGFRL1 | 53834 | 2.11593 | 6.08559 | 5.36E-11 | 8.28E-10 | up-regulated |
| CHST8 | 64377 | -2.7341 | -3.1112 | 5.40E-11 | 8.35E-10 | down-regulated |
| C1QTNF9 | 338872 | -2.0164 | -1.2651 | 5.45E-11 | 8.41E-10 | down-regulated |
| ST8SIA3 | 51046 | -3.6893 | -2.3266 | 5.50E-11 | 8.48E-10 | down-regulated |
| KIF5A | 3798 | -2.9164 | 0.27678 | 5.56E-11 | 8.56E-10 | down-regulated |
| LRRN4CL | 221091 | -2.015 | 1.80736 | 5.69E-11 | 8.74E-10 | down-regulated |
| STC2 | 8614 | 3.47649 | 5.24673 | 5.75E-11 | 8.82E-10 | up-regulated |
| DSCAML1 | 57453 | -2.9083 | -0.3277 | 5.86E-11 | 8.98E-10 | down-regulated |
| NOS1 | 4842 | -4.0278 | -0.6101 | 6.06E-11 | 9.27E-10 | down-regulated |
| MLXIPL | 51085 | 3.5845 | 5.69192 | 6.24E-11 | 9.53E-10 | up-regulated |
| MOBP | 4336 | -2.6126 | -2.6993 | 6.27E-11 | 9.56E-10 | down-regulated |
| GALNTL1 | 57452 | -2.6543 | 0.83288 | 6.42E-11 | 9.77E-10 | down-regulated |
| ZDHHC15 | 158866 | -2.2198 | -0.6273 | 6.43E-11 | 9.78E-10 | down-regulated |
| SH3TC2 | 79628 | 3.02102 | 3.95247 | 6.56E-11 | 9.95E-10 | up-regulated |
| PKD1L2 | 114780 | -2.5553 | -2.69 | 6.56E-11 | 9.95E-10 | down-regulated |
| FABP2 | 2169 | -2.9691 | 3.73657 | 6.62E-11 | 1.00E-09 | down-regulated |
| C2CD4A | 145741 | 3.96361 | 4.17926 | 7.31E-11 | 1.10E-09 | up-regulated |
| EVI2A | 2123 | -2.0203 | 2.891 | 7.34E-11 | 1.10E-09 | down-regulated |
| CYP27B1 | 1594 | 2.03283 | 1.79807 | 7.34E-11 | 1.10E-09 | up-regulated |
| C10orf140 | 387640 | -2.0707 | -1.2911 | 7.36E-11 | 1.10E-09 | down-regulated |
| PDE3A | 5139 | -2.1615 | 3.89026 | 7.38E-11 | 1.11E-09 | down-regulated |
| UGT2A3 | 79799 | -3.0749 | 4.60723 | 7.49E-11 | 1.12E-09 | down-regulated |
| EGFL6 | 25975 | 3.99542 | 1.99753 | 7.68E-11 | 1.15E-09 | up-regulated |
| NAALADL1 | 10004 | -2.0443 | 2.47968 | 7.68E-11 | 1.15E-09 | down-regulated |
| SULT1B1 | 27284 | -2.2667 | 3.61939 | 7.83E-11 | 1.17E-09 | down-regulated |
| ProSAPiP1 | 9762 | 2.30638 | 5.41428 | 7.89E-11 | 1.18E-09 | up-regulated |
| NIPAL4 | 348938 | -2.0487 | -0.3501 | 8.18E-11 | 1.22E-09 | down-regulated |
| CCNO | 10309 | 3.20809 | 2.46826 | 8.42E-11 | 1.25E-09 | up-regulated |
| UGT1A10 | 54575 | -2.7508 | 4.16597 | 9.97E-11 | 1.47E-09 | down-regulated |
| ANGPT1 | 284 | -2.0776 | 2.15159 | 1.13E-10 | 1.64E-09 | down-regulated |
| WISP2 | 8839 | -3.4783 | 1.00152 | 1.13E-10 | 1.65E-09 | down-regulated |
| EVPL | 2125 | 2.14873 | 6.91528 | 1.17E-10 | 1.70E-09 | up-regulated |
| CHRM2 | 1129 | -4.1076 | 0.72789 | 1.18E-10 | 1.71E-09 | down-regulated |
| KIAA1257 | 57501 | 3.48556 | 2.26178 | 1.21E-10 | 1.75E-09 | up-regulated |
| UNC5CL | 222643 | 3.08258 | 4.08634 | 1.22E-10 | 1.76E-09 | up-regulated |
| GRHL1 | 29841 | 3.00519 | 2.80398 | 1.26E-10 | 1.81E-09 | up-regulated |
| C6orf168 | 84553 | -2.331 | 1.50637 | 1.27E-10 | 1.83E-09 | down-regulated |
| OTOP3 | 347741 | -5.0729 | -2.5626 | 1.33E-10 | 1.92E-09 | down-regulated |
| SLC30A10 | 55532 | -3.7365 | 0.92895 | 1.34E-10 | 1.92E-09 | down-regulated |
| MADCAM1 | 8174 | -2.8743 | 0.51498 | 1.34E-10 | 1.93E-09 | down-regulated |
| DACH1 | 1602 | 2.77368 | 5.68212 | 1.42E-10 | 2.04E-09 | up-regulated |
| CLEC17A | 388512 | -3.1427 | -1.9243 | 1.48E-10 | 2.11E-09 | down-regulated |
| SLC30A8 | 169026 | -2.6695 | -3.2859 | 1.50E-10 | 2.14E-09 | down-regulated |
| NKD1 | 85407 | 4.86534 | 6.21341 | 1.51E-10 | 2.15E-09 | up-regulated |
| RHPN1 | 114822 | 3.05972 | 4.14193 | 1.52E-10 | 2.16E-09 | up-regulated |
| RTN1 | 6252 | -2.131 | 1.68009 | 1.53E-10 | 2.17E-09 | down-regulated |
| PDE6A | 5145 | -2.9892 | 0.87837 | 1.56E-10 | 2.20E-09 | down-regulated |
| MS4A1 | 931 | -3.337 | 2.27829 | 1.56E-10 | 2.21E-09 | down-regulated |
| HSD3B2 | 3284 | -3.6593 | -1.0327 | 1.65E-10 | 2.32E-09 | down-regulated |
| DSCR6 | 53820 | 4.40074 | 0.96708 | 1.75E-10 | 2.46E-09 | up-regulated |
| TMEM171 | 134285 | -2.041 | 4.16157 | 1.90E-10 | 2.67E-09 | down-regulated |
| SLC6A19 | 340024 | -4.226 | 2.33403 | 1.96E-10 | 2.73E-09 | down-regulated |
| GRIA3 | 2892 | -2.5207 | -1.6111 | 1.96E-10 | 2.73E-09 | down-regulated |
| TRIM40 | 135644 | -2.6652 | 1.37124 | 2.18E-10 | 3.03E-09 | down-regulated |
| SCGB2A1 | 4246 | -3.3276 | 0.39387 | 2.29E-10 | 3.18E-09 | down-regulated |
| DHRS9 | 10170 | -3.0578 | 4.67312 | 2.52E-10 | 3.47E-09 | down-regulated |
| FJX1 | 24147 | 2.43854 | 2.23944 | 2.61E-10 | 3.59E-09 | up-regulated |
| MEGF10 | 84466 | -2.4959 | -1.2447 | 2.63E-10 | 3.61E-09 | down-regulated |
| PPP1R1A | 5502 | -3.5183 | 1.27769 | 2.68E-10 | 3.67E-09 | down-regulated |
| RELL2 | 285613 | 2.63286 | 2.49508 | 2.82E-10 | 3.86E-09 | up-regulated |
| ZNF483 | 158399 | -2.2111 | -2.1212 | 2.98E-10 | 4.06E-09 | down-regulated |
| PLA2G5 | 5322 | -2.6383 | 0.9298 | 3.03E-10 | 4.13E-09 | down-regulated |
| SLC4A11 | 83959 | 4.74921 | 2.80154 | 3.14E-10 | 4.26E-09 | up-regulated |
| DNMT3B | 1789 | 2.41136 | 2.80312 | 3.14E-10 | 4.26E-09 | up-regulated |
| PRND | 23627 | -3.0281 | 0.68455 | 3.17E-10 | 4.29E-09 | down-regulated |
| SP6 | 80320 | 2.37073 | 4.68647 | 3.21E-10 | 4.34E-09 | up-regulated |
| CYS1 | 192668 | -2.1579 | 2.1524 | 3.21E-10 | 4.34E-09 | down-regulated |
| TPO | 7173 | -2.6214 | -1.9862 | 3.22E-10 | 4.35E-09 | down-regulated |
| SLC9A2 | 6549 | -2.4155 | 4.72173 | 3.23E-10 | 4.36E-09 | down-regulated |
| LY6G6F | 259215 | 6.20027 | -1.4094 | 3.29E-10 | 4.43E-09 | up-regulated |
| SECTM1 | 6398 | -2.064 | 4.7467 | 3.41E-10 | 4.59E-09 | down-regulated |
| DES | 1674 | -3.4288 | 9.108 | 3.47E-10 | 4.66E-09 | down-regulated |
| KCNN3 | 3782 | -2.1648 | 0.82315 | 3.53E-10 | 4.72E-09 | down-regulated |
| HS3ST6 | 64711 | -3.8325 | -2.1683 | 3.63E-10 | 4.85E-09 | down-regulated |
| LEPREL1 | 55214 | -2.0779 | 4.09655 | 3.69E-10 | 4.92E-09 | down-regulated |
| CASQ2 | 845 | -3.7206 | 3.08944 | 3.72E-10 | 4.96E-09 | down-regulated |
| PI16 | 221476 | -3.5609 | 2.41207 | 3.80E-10 | 5.05E-09 | down-regulated |
| C1orf170 | 84808 | 3.60571 | 0.73079 | 3.85E-10 | 5.12E-09 | up-regulated |
| CACNA1D | 776 | 2.09638 | 4.20074 | 3.94E-10 | 5.23E-09 | up-regulated |
| DCN | 1634 | -2.1042 | 8.27668 | 4.59E-10 | 6.05E-09 | down-regulated |
| SLC22A11 | 55867 | 5.34394 | 1.50209 | 4.60E-10 | 6.06E-09 | up-regulated |
| ZNF229 | 7772 | -2.0767 | -0.2091 | 4.88E-10 | 6.40E-09 | down-regulated |
| NPY6R | 4888 | -3.2325 | -1.3905 | 4.90E-10 | 6.43E-09 | down-regulated |
| GABRE | 2564 | 2.97056 | 4.6182 | 4.91E-10 | 6.43E-09 | up-regulated |
| KIAA1239 | 57495 | -2.6045 | -0.7722 | 5.05E-10 | 6.60E-09 | down-regulated |
| C8orf46 | 254778 | -2.6953 | -2.1612 | 5.05E-10 | 6.60E-09 | down-regulated |
| SCNN1B | 6338 | -3.2516 | 4.95477 | 5.20E-10 | 6.77E-09 | down-regulated |
| CORO6 | 84940 | -2.2259 | -0.6226 | 5.29E-10 | 6.87E-09 | down-regulated |
| LGR5 | 8549 | 3.39137 | 6.64911 | 5.54E-10 | 7.18E-09 | up-regulated |
| KIF14 | 9928 | 2.00221 | 3.83467 | 6.06E-10 | 7.77E-09 | up-regulated |
| GLI3 | 2737 | -2.2071 | 2.80353 | 6.25E-10 | 8.00E-09 | down-regulated |
| SLITRK5 | 26050 | -2.8155 | -1.2526 | 6.33E-10 | 8.09E-09 | down-regulated |
| ABCA10 | 10349 | -2.2032 | -0.3936 | 6.67E-10 | 8.50E-09 | down-regulated |
| SYT7 | 9066 | 2.19514 | 6.48218 | 6.78E-10 | 8.63E-09 | up-regulated |
| PPP2R2B | 5521 | -2.303 | -0.2428 | 7.03E-10 | 8.91E-09 | down-regulated |
| CTHRC1 | 115908 | 3.8229 | 5.21697 | 7.18E-10 | 9.09E-09 | up-regulated |
| ABCB5 | 340273 | -3.345 | -2.3131 | 7.23E-10 | 9.14E-09 | down-regulated |
| CCDC150 | 284992 | 2.80766 | 1.16307 | 7.74E-10 | 9.72E-09 | up-regulated |
| C1orf95 | 375057 | -3.0069 | 1.15553 | 7.75E-10 | 9.73E-09 | down-regulated |
| HIF3A | 64344 | -2.3451 | 1.53817 | 7.80E-10 | 9.78E-09 | down-regulated |
| LIX1 | 167410 | -3.3611 | -2.032 | 7.82E-10 | 9.79E-09 | down-regulated |
| FER1L4 | 80307 | 4.56023 | 4.21372 | 8.00E-10 | 1.00E-08 | up-regulated |
| IL1RAPL1 | 11141 | -2.8154 | -3.76 | 8.59E-10 | 1.07E-08 | down-regulated |
| GPR44 | 11251 | -2.089 | 0.89167 | 8.64E-10 | 1.08E-08 | down-regulated |
| PSAT1 | 29968 | 2.84449 | 6.04333 | 8.70E-10 | 1.08E-08 | up-regulated |
| CR2 | 1380 | -3.4991 | 3.01455 | 8.96E-10 | 1.11E-08 | down-regulated |
| C6orf15 | 29113 | 12.0202 | 3.42155 | 9.06E-10 | 1.12E-08 | up-regulated |
| CD1C | 911 | -2.3372 | 0.58759 | 9.48E-10 | 1.17E-08 | down-regulated |
| KCNJ14 | 3770 | 2.25062 | 1.26247 | 9.56E-10 | 1.18E-08 | up-regulated |
| TDGF1 | 6997 | 3.21584 | 5.48121 | 9.91E-10 | 1.22E-08 | up-regulated |
| LOC646627 | 646627 | -3.0049 | 4.01044 | 9.95E-10 | 1.22E-08 | down-regulated |
| HIPK4 | 147746 | -2.2058 | -2.9041 | 1.01E-09 | 1.24E-08 | down-regulated |
| EDN3 | 1908 | -2.5052 | 3.80851 | 1.02E-09 | 1.25E-08 | down-regulated |
| SPERT | 220082 | 7.08029 | -0.0058 | 1.02E-09 | 1.25E-08 | up-regulated |
| SLC16A9 | 220963 | -2.1283 | 4.00901 | 1.05E-09 | 1.29E-08 | down-regulated |
| SFTA2 | 389376 | 6.72875 | 1.46398 | 1.08E-09 | 1.32E-08 | up-regulated |
| CRYAB | 1410 | -2.1882 | 4.17157 | 1.09E-09 | 1.33E-08 | down-regulated |
| VSIG2 | 23584 | -3.196 | 5.42487 | 1.14E-09 | 1.39E-08 | down-regulated |
| CNTNAP4 | 85445 | -3.8398 | -3.5028 | 1.19E-09 | 1.44E-08 | down-regulated |
| PTK7 | 5754 | 2.32166 | 6.84169 | 1.22E-09 | 1.48E-08 | up-regulated |
| PLAC2 | 257000 | -2.3658 | 0.29767 | 1.36E-09 | 1.64E-08 | down-regulated |
| ACSL6 | 23305 | 4.95807 | 3.79474 | 1.37E-09 | 1.65E-08 | up-regulated |
| LOC100127888 | 100127888 | 4.45384 | 3.20489 | 1.42E-09 | 1.70E-08 | up-regulated |
| SCG2 | 7857 | -2.2622 | 2.69183 | 1.42E-09 | 1.71E-08 | down-regulated |
| LY6G6D | 58530 | 5.45938 | 4.92068 | 1.49E-09 | 1.78E-08 | up-regulated |
| NOTUM | 147111 | 7.89644 | 6.39198 | 1.50E-09 | 1.79E-08 | up-regulated |
| HOXD12 | 3238 | -3.4223 | -2.3095 | 1.51E-09 | 1.81E-08 | down-regulated |
| PENK | 5179 | -3.6127 | 0.27463 | 1.57E-09 | 1.87E-08 | down-regulated |
| ZG16 | 653808 | -4.1413 | 7.61269 | 1.59E-09 | 1.90E-08 | down-regulated |
| SLC35F1 | 222553 | -2.2479 | 0.21005 | 1.61E-09 | 1.92E-08 | down-regulated |
| ATP6V0D2 | 245972 | -2.1806 | 0.66891 | 1.68E-09 | 2.01E-08 | down-regulated |
| CORO2B | 10391 | -2.1099 | 0.82209 | 1.71E-09 | 2.03E-08 | down-regulated |
| GLS2 | 27165 | 2.94778 | 2.6317 | 1.75E-09 | 2.07E-08 | up-regulated |
| RHEBL1 | 121268 | 2.26203 | 0.93999 | 1.75E-09 | 2.08E-08 | up-regulated |
| LILRB5 | 10990 | -2.2816 | 1.75117 | 1.76E-09 | 2.08E-08 | down-regulated |
| TMEM82 | 388595 | -2.4243 | 1.42851 | 1.80E-09 | 2.13E-08 | down-regulated |
| C1orf135 | 79000 | 2.31702 | 2.65906 | 1.81E-09 | 2.14E-08 | up-regulated |
| BNC2 | 54796 | -2.1203 | 2.96449 | 1.86E-09 | 2.18E-08 | down-regulated |
| SPATA12 | 353324 | 2.30561 | 0.22795 | 1.89E-09 | 2.22E-08 | up-regulated |
| HAND1 | 9421 | -4.2215 | 1.82041 | 1.92E-09 | 2.25E-08 | down-regulated |
| CEL | 1056 | 7.24546 | 6.41556 | 1.98E-09 | 2.31E-08 | up-regulated |
| LY9 | 4063 | -2.2919 | 0.65734 | 2.01E-09 | 2.35E-08 | down-regulated |
| NXPH2 | 11249 | -3.5472 | -3.2321 | 2.11E-09 | 2.46E-08 | down-regulated |
| MSH5 | 4439 | 2.02049 | 4.2756 | 2.12E-09 | 2.47E-08 | up-regulated |
| HSPA2 | 3306 | -2.055 | 5.00774 | 2.17E-09 | 2.52E-08 | down-regulated |
| CXCL13 | 10563 | -2.6737 | 2.97054 | 2.18E-09 | 2.53E-08 | down-regulated |
| ULBP1 | 80329 | 3.55267 | 0.19181 | 2.19E-09 | 2.54E-08 | up-regulated |
| PLEKHG4B | 153478 | -2.3665 | -1.4385 | 2.27E-09 | 2.62E-08 | down-regulated |
| LRRN2 | 10446 | -2.4013 | 2.9531 | 2.29E-09 | 2.64E-08 | down-regulated |
| CD163L1 | 283316 | -2.0981 | 3.09609 | 2.31E-09 | 2.67E-08 | down-regulated |
| RECQL4 | 9401 | 2.42068 | 5.37212 | 2.33E-09 | 2.69E-08 | up-regulated |
| JAG2 | 3714 | 2.00569 | 5.39199 | 2.35E-09 | 2.71E-08 | up-regulated |
| KLRF1 | 51348 | -2.16 | -2.5288 | 2.38E-09 | 2.74E-08 | down-regulated |
| CST2 | 1470 | 6.03079 | 0.66711 | 2.41E-09 | 2.78E-08 | up-regulated |
| CLCA4 | 22802 | -4.3373 | 6.73707 | 2.45E-09 | 2.81E-08 | down-regulated |
| S100A2 | 6273 | 4.70938 | 3.50631 | 2.46E-09 | 2.82E-08 | up-regulated |
| BCAS1 | 8537 | -2.1675 | 6.01659 | 2.50E-09 | 2.87E-08 | down-regulated |
| MYPN | 84665 | -2.6621 | -1.308 | 2.56E-09 | 2.92E-08 | down-regulated |
| EFCAB1 | 79645 | -2.0035 | -2.7602 | 2.56E-09 | 2.93E-08 | down-regulated |
| NKPD1 | 284353 | 3.12921 | -0.0812 | 2.59E-09 | 2.95E-08 | up-regulated |
| HOXB8 | 3218 | 3.94761 | 4.85972 | 2.62E-09 | 2.99E-08 | up-regulated |
| GRIA2 | 2891 | -2.9761 | -3.6372 | 2.64E-09 | 3.01E-08 | down-regulated |
| CES3 | 23491 | -2.1562 | 5.27305 | 2.73E-09 | 3.10E-08 | down-regulated |
| NSUN5P2 | 260294 | 2.3484 | 4.85868 | 2.73E-09 | 3.10E-08 | up-regulated |
| NSUN5P1 | 155400 | 2.47293 | 3.43962 | 2.75E-09 | 3.12E-08 | up-regulated |
| DDR2 | 4921 | -2.2191 | 3.27977 | 2.78E-09 | 3.15E-08 | down-regulated |
| LCN6 | 158062 | -3.2543 | -3.2723 | 2.92E-09 | 3.30E-08 | down-regulated |
| E2F7 | 144455 | 2.07602 | 3.08708 | 2.95E-09 | 3.33E-08 | up-regulated |
| KIAA1751 | 85452 | 6.6629 | -1.7208 | 2.98E-09 | 3.35E-08 | up-regulated |
| C17orf96 | 100170841 | 3.75178 | 4.36765 | 3.09E-09 | 3.46E-08 | up-regulated |
| KCNH8 | 131096 | 2.92569 | 2.73559 | 3.15E-09 | 3.52E-08 | up-regulated |
| CPXM2 | 119587 | -2.2793 | 4.34238 | 3.15E-09 | 3.52E-08 | down-regulated |
| HKDC1 | 80201 | 2.23551 | 6.31094 | 3.23E-09 | 3.60E-08 | up-regulated |
| DPYSL5 | 56896 | -3.068 | -2.0261 | 3.23E-09 | 3.60E-08 | down-regulated |
| ACSM1 | 116285 | -2.2181 | -1.1222 | 3.32E-09 | 3.69E-08 | down-regulated |
| SHISA2 | 387914 | 3.23935 | 2.05462 | 3.38E-09 | 3.75E-08 | up-regulated |
| CLDN8 | 9073 | -4.3815 | 4.17094 | 3.40E-09 | 3.77E-08 | down-regulated |
| ABCD2 | 225 | -2.2028 | -0.8037 | 3.41E-09 | 3.78E-08 | down-regulated |
| NEXN | 91624 | -2.0475 | 4.21251 | 3.57E-09 | 3.93E-08 | down-regulated |
| SNORA39 | 677821 | 2.65175 | -0.659 | 3.78E-09 | 4.15E-08 | up-regulated |
| TNS4 | 84951 | 3.40901 | 6.93633 | 3.84E-09 | 4.20E-08 | up-regulated |
| CASQ1 | 844 | -2.4586 | -1.6914 | 3.91E-09 | 4.26E-08 | down-regulated |
| LRRK2 | 120892 | -2.1562 | 2.18408 | 3.98E-09 | 4.34E-08 | down-regulated |
| C4orf39 | 152756 | -2.1898 | -1.9504 | 4.01E-09 | 4.37E-08 | down-regulated |
| PMEPA1 | 56937 | 2.10005 | 7.97399 | 4.02E-09 | 4.37E-08 | up-regulated |
| SLC26A7 | 115111 | -2.0023 | -1.3839 | 4.12E-09 | 4.47E-08 | down-regulated |
| LOC399959 | 399959 | -2.0911 | 2.75475 | 4.13E-09 | 4.48E-08 | down-regulated |
| SLCO4C1 | 353189 | -2.803 | 0.06893 | 4.45E-09 | 4.80E-08 | down-regulated |
| MST1P9 | 11223 | -2.5858 | 1.42843 | 4.56E-09 | 4.92E-08 | down-regulated |
| MFAP4 | 4239 | -2.0131 | 6.23391 | 4.58E-09 | 4.93E-08 | down-regulated |
| RGS22 | 26166 | -2.7416 | -2.8746 | 4.63E-09 | 4.98E-08 | down-regulated |
| SEZ6L | 23544 | -2.7702 | -2.5 | 4.78E-09 | 5.14E-08 | down-regulated |
| MEP1B | 4225 | -2.9356 | -0.5765 | 4.79E-09 | 5.14E-08 | down-regulated |
| TTLL6 | 284076 | -2.1359 | 1.49138 | 5.01E-09 | 5.37E-08 | down-regulated |
| TSPAN2 | 10100 | -2.0297 | 4.12226 | 5.10E-09 | 5.45E-08 | down-regulated |
| CWH43 | 80157 | -3.4277 | 2.82144 | 5.42E-09 | 5.77E-08 | down-regulated |
| SRPX2 | 27286 | 3.17689 | 5.00121 | 5.44E-09 | 5.78E-08 | up-regulated |
| RTEL1 | 51750 | 2.29852 | 5.9741 | 5.55E-09 | 5.88E-08 | up-regulated |
| MYOC | 4653 | -4.2694 | 0.7275 | 5.55E-09 | 5.88E-08 | down-regulated |
| MYADML2 | 255275 | 5.21042 | 1.90202 | 5.71E-09 | 6.03E-08 | up-regulated |
| FGF19 | 9965 | 7.65891 | 2.3876 | 6.12E-09 | 6.46E-08 | up-regulated |
| MFAP2 | 4237 | 2.45354 | 4.66973 | 6.17E-09 | 6.51E-08 | up-regulated |
| HTR3A | 3359 | -3.0402 | -1.2078 | 6.18E-09 | 6.51E-08 | down-regulated |
| SERPINA9 | 327657 | -4.8905 | -1.2242 | 6.85E-09 | 7.19E-08 | down-regulated |
| MTUS2 | 23281 | -2.1845 | -1.4538 | 7.51E-09 | 7.84E-08 | down-regulated |
| PLCXD3 | 345557 | -3.1424 | -0.5341 | 7.69E-09 | 8.01E-08 | down-regulated |
| NR4A3 | 8013 | -2.3483 | 3.09733 | 8.46E-09 | 8.77E-08 | down-regulated |
| KRT17 | 3872 | 5.62027 | 4.69837 | 8.79E-09 | 9.09E-08 | up-regulated |
| TMPRSS3 | 64699 | 3.08537 | 3.54458 | 9.01E-09 | 9.30E-08 | up-regulated |
| LOC100126784 | 100126784 | -2.0773 | -0.8119 | 9.32E-09 | 9.59E-08 | down-regulated |
| KLHL31 | 401265 | 2.25804 | 1.0409 | 9.53E-09 | 9.79E-08 | up-regulated |
| SNAP25 | 6616 | -2.737 | 0.3088 | 9.81E-09 | 1.01E-07 | down-regulated |
| FGF10 | 2255 | -2.6559 | -1.9552 | 1.03E-08 | 1.05E-07 | down-regulated |
| ANXA9 | 8416 | 2.28893 | 2.90509 | 1.05E-08 | 1.07E-07 | up-regulated |
| TUB | 7275 | -2.0212 | 1.49021 | 1.05E-08 | 1.07E-07 | down-regulated |
| DLX4 | 1748 | 2.70204 | -0.5363 | 1.08E-08 | 1.10E-07 | up-regulated |
| ADH1C | 126 | -3.1008 | 5.63734 | 1.12E-08 | 1.13E-07 | down-regulated |
| XKRX | 402415 | 2.97051 | 3.05746 | 1.12E-08 | 1.13E-07 | up-regulated |
| P2RY4 | 5030 | -2.4971 | -2.5558 | 1.12E-08 | 1.14E-07 | down-regulated |
| SLC10A4 | 201780 | -2.1339 | -1.3403 | 1.13E-08 | 1.15E-07 | down-regulated |
| DARC | 2532 | -2.2633 | 3.24919 | 1.20E-08 | 1.21E-07 | down-regulated |
| GCNT4 | 51301 | -2.0778 | 0.28859 | 1.22E-08 | 1.22E-07 | down-regulated |
| KLK10 | 5655 | 4.65456 | 4.89388 | 1.23E-08 | 1.23E-07 | up-regulated |
| MT1X | 4501 | -2.0311 | 5.02723 | 1.23E-08 | 1.24E-07 | down-regulated |
| TNFRSF13C | 115650 | -2.2289 | -1.4384 | 1.25E-08 | 1.25E-07 | down-regulated |
| ADAM12 | 8038 | 4.04148 | 4.53229 | 1.26E-08 | 1.26E-07 | up-regulated |
| CNTN1 | 1272 | -3.1491 | 0.94206 | 1.30E-08 | 1.30E-07 | down-regulated |
| POU3F3 | 5455 | -2.9226 | -3.5426 | 1.32E-08 | 1.32E-07 | down-regulated |
| TDGF3 | 6998 | 3.0352 | 2.09842 | 1.34E-08 | 1.34E-07 | up-regulated |
| ENTPD3 | 956 | -2.6031 | 0.60536 | 1.37E-08 | 1.37E-07 | down-regulated |
| FCRL1 | 115350 | -3.0799 | -1.0775 | 1.40E-08 | 1.38E-07 | down-regulated |
| CA10 | 56934 | -2.4179 | -2.8388 | 1.44E-08 | 1.43E-07 | down-regulated |
| IER5L | 389792 | 2.10836 | 4.00649 | 1.51E-08 | 1.49E-07 | up-regulated |
| LOC134466 | 134466 | -2.009 | -0.9522 | 1.52E-08 | 1.50E-07 | down-regulated |
| WNT9A | 7483 | -2.23 | -0.9039 | 1.54E-08 | 1.51E-07 | down-regulated |
| CCDC78 | 124093 | 4.00468 | 1.33376 | 1.63E-08 | 1.60E-07 | up-regulated |
| TMEM130 | 222865 | -2.3298 | 0.2913 | 1.64E-08 | 1.60E-07 | down-regulated |
| NCAN | 1463 | -2.6711 | -3.499 | 1.64E-08 | 1.61E-07 | down-regulated |
| SYNGR1 | 9145 | -2.1357 | 2.39917 | 1.66E-08 | 1.62E-07 | down-regulated |
| TRIM29 | 23650 | 3.06855 | 6.44723 | 1.70E-08 | 1.66E-07 | up-regulated |
| SORCS3 | 22986 | -3.1003 | -3.0344 | 1.72E-08 | 1.68E-07 | down-regulated |
| UCN2 | 90226 | 4.39796 | -0.4858 | 1.73E-08 | 1.69E-07 | up-regulated |
| KCNIP1 | 30820 | -2.529 | -2.7676 | 1.76E-08 | 1.72E-07 | down-regulated |
| CST4 | 1472 | 6.48627 | 0.65145 | 1.81E-08 | 1.76E-07 | up-regulated |
| SP5 | 389058 | 3.59413 | 3.80952 | 1.82E-08 | 1.76E-07 | up-regulated |
| KLHL10 | 317719 | -2.2125 | -3.5104 | 1.83E-08 | 1.77E-07 | down-regulated |
| AMELX | 265 | 6.10497 | -1.5014 | 1.86E-08 | 1.80E-07 | up-regulated |
| CA9 | 768 | 5.89392 | 5.14898 | 1.86E-08 | 1.80E-07 | up-regulated |
| KCTD8 | 386617 | -3.0784 | -2.9975 | 1.87E-08 | 1.80E-07 | down-regulated |
| LRRC6 | 23639 | 2.38159 | 2.33121 | 1.90E-08 | 1.84E-07 | up-regulated |
| COMP | 1311 | 6.79293 | 4.8498 | 2.00E-08 | 1.92E-07 | up-regulated |
| PPP1R3C | 5507 | -2.307 | 2.35842 | 2.04E-08 | 1.96E-07 | down-regulated |
| FANCB | 2187 | 2.03975 | 1.25873 | 2.06E-08 | 1.98E-07 | up-regulated |
| FAM150A | 389658 | 3.98302 | 1.34812 | 2.09E-08 | 2.00E-07 | up-regulated |
| SLC22A3 | 6581 | 2.11747 | 5.09488 | 2.14E-08 | 2.05E-07 | up-regulated |
| TMPRSS13 | 84000 | 4.07888 | 3.71424 | 2.17E-08 | 2.07E-07 | up-regulated |
| WDR66 | 144406 | 2.65344 | 1.03987 | 2.27E-08 | 2.16E-07 | up-regulated |
| GPR15 | 2838 | -3.0734 | 0.44596 | 2.33E-08 | 2.21E-07 | down-regulated |
| C13orf30 | 144809 | -2.4465 | -2.5544 | 2.33E-08 | 2.21E-07 | down-regulated |
| ENHO | 375704 | -2.0354 | -0.1969 | 2.41E-08 | 2.28E-07 | down-regulated |
| C8ORFK29 | 340393 | 4.17896 | -0.3422 | 2.41E-08 | 2.28E-07 | up-regulated |
| SHH | 6469 | 2.25601 | 2.79329 | 2.45E-08 | 2.31E-07 | up-regulated |
| WNT3 | 7473 | 2.59405 | 0.2647 | 2.46E-08 | 2.32E-07 | up-regulated |
| WFIKKN2 | 124857 | -2.4394 | -1.9686 | 2.46E-08 | 2.32E-07 | down-regulated |
| MC1R | 4157 | 2.10313 | 2.56689 | 2.48E-08 | 2.33E-07 | up-regulated |
| FAM40B | 57464 | 2.26307 | 2.44986 | 2.54E-08 | 2.38E-07 | up-regulated |
| SERPINA4 | 5267 | 9.51237 | 1.74411 | 2.61E-08 | 2.45E-07 | up-regulated |
| PAPPA2 | 60676 | -2.8148 | -0.6912 | 2.66E-08 | 2.50E-07 | down-regulated |
| C2orf58 | 285154 | -2.0063 | -2.2984 | 2.67E-08 | 2.50E-07 | down-regulated |
| SCNN1G | 6340 | -3.5035 | 1.62994 | 2.73E-08 | 2.55E-07 | down-regulated |
| PCK1 | 5105 | -2.4567 | 6.77365 | 2.76E-08 | 2.57E-07 | down-regulated |
| PROX1 | 5629 | 2.26653 | 3.73168 | 2.81E-08 | 2.61E-07 | up-regulated |
| DLGAP2 | 9228 | -2.1972 | -2.8838 | 2.82E-08 | 2.62E-07 | down-regulated |
| MOGAT2 | 80168 | -2.5001 | 2.83191 | 2.85E-08 | 2.65E-07 | down-regulated |
| GHR | 2690 | -2.3601 | 2.88768 | 3.01E-08 | 2.79E-07 | down-regulated |
| TUBAL3 | 79861 | -2.0661 | 2.87389 | 3.01E-08 | 2.79E-07 | down-regulated |
| CHST5 | 23563 | -2.786 | 4.7085 | 3.04E-08 | 2.81E-07 | down-regulated |
| BTBD16 | 118663 | 3.74455 | -0.4456 | 3.04E-08 | 2.82E-07 | up-regulated |
| ADAM33 | 80332 | -2.2302 | 2.19428 | 3.07E-08 | 2.84E-07 | down-regulated |
| GOLGA7B | 401647 | 2.80019 | 3.57534 | 3.13E-08 | 2.89E-07 | up-regulated |
| C20orf46 | 55321 | 3.33659 | 2.86114 | 3.30E-08 | 3.04E-07 | up-regulated |
| KLK8 | 11202 | 10.4388 | 1.85008 | 3.42E-08 | 3.15E-07 | up-regulated |
| CA4 | 762 | -3.6656 | 5.61944 | 3.53E-08 | 3.24E-07 | down-regulated |
| HCN4 | 10021 | -2.5639 | -3.0872 | 3.56E-08 | 3.26E-07 | down-regulated |
| AKR1C4 | 1109 | 10.3645 | 1.77739 | 3.60E-08 | 3.29E-07 | up-regulated |
| LOC148709 | 148709 | 2.36687 | 0.97417 | 3.61E-08 | 3.30E-07 | up-regulated |
| MMP1 | 4312 | 5.11688 | 6.63333 | 3.63E-08 | 3.31E-07 | up-regulated |
| STK31 | 56164 | 4.63833 | 1.57611 | 3.84E-08 | 3.48E-07 | up-regulated |
| ZNF835 | 90485 | -2.0342 | -2.012 | 3.89E-08 | 3.53E-07 | down-regulated |
| GSDMC | 56169 | 3.3767 | -2.2027 | 3.93E-08 | 3.56E-07 | up-regulated |
| CELP | 1057 | 5.93445 | 3.2239 | 3.97E-08 | 3.59E-07 | up-regulated |
| KRTAP5-1 | 387264 | 2.64243 | 0.46812 | 3.98E-08 | 3.59E-07 | up-regulated |
| OTUB2 | 78990 | 2.14915 | 3.40508 | 4.05E-08 | 3.66E-07 | up-regulated |
| CYP2B6 | 1555 | 3.85088 | 5.39907 | 4.06E-08 | 3.67E-07 | up-regulated |
| MYH2 | 4620 | -3.6159 | -3.0903 | 4.12E-08 | 3.71E-07 | down-regulated |
| KRT6B | 3854 | 6.85046 | 4.0581 | 4.12E-08 | 3.71E-07 | up-regulated |
| FOSB | 2354 | -2.4682 | 5.74692 | 4.31E-08 | 3.86E-07 | down-regulated |
| CHI3L1 | 1116 | 3.33417 | 5.21711 | 4.34E-08 | 3.89E-07 | up-regulated |
| SV2B | 9899 | -2.1621 | 0.54647 | 4.37E-08 | 3.91E-07 | down-regulated |
| CD177 | 57126 | -3.4569 | 6.06515 | 4.42E-08 | 3.94E-07 | down-regulated |
| CD79A | 973 | -2.3908 | 3.64062 | 4.51E-08 | 4.02E-07 | down-regulated |
| MMP3 | 4314 | 5.62658 | 6.02414 | 4.58E-08 | 4.07E-07 | up-regulated |
| FGF18 | 8817 | 2.84197 | 0.77748 | 4.62E-08 | 4.11E-07 | up-regulated |
| ULBP2 | 80328 | 3.74497 | 0.76909 | 4.67E-08 | 4.15E-07 | up-regulated |
| LOC284578 | 284578 | -2.2165 | -0.3105 | 4.69E-08 | 4.16E-07 | down-regulated |
| KRT75 | 9119 | 8.21536 | 0.91988 | 5.00E-08 | 4.42E-07 | up-regulated |
| C20orf151 | 140893 | 2.01347 | 4.17796 | 5.05E-08 | 4.45E-07 | up-regulated |
| MPPED2 | 744 | -2.153 | -0.2235 | 5.28E-08 | 4.64E-07 | down-regulated |
| SHISA7 | 729956 | -2.1813 | -3.3939 | 5.28E-08 | 4.64E-07 | down-regulated |
| KRT1 | 3848 | -2.7429 | -1.7391 | 5.37E-08 | 4.72E-07 | down-regulated |
| STON1-GTF2A1L | 286749 | -3.9644 | -0.9313 | 5.52E-08 | 4.85E-07 | down-regulated |
| PROKR1 | 10887 | -2.5441 | -3.6722 | 5.55E-08 | 4.86E-07 | down-regulated |
| ATP6V1C2 | 245973 | 2.27462 | 3.55639 | 5.60E-08 | 4.90E-07 | up-regulated |
| TG | 7038 | 4.07467 | 3.38539 | 5.79E-08 | 5.05E-07 | up-regulated |
| KLRC1 | 3821 | -2.1602 | -1.9079 | 5.80E-08 | 5.06E-07 | down-regulated |
| SSTR3 | 6753 | -2.405 | -3.4006 | 5.83E-08 | 5.08E-07 | down-regulated |
| ZNF676 | 163223 | -2.5222 | -2.9911 | 5.84E-08 | 5.08E-07 | down-regulated |
| LOC286467 | 286467 | 5.60025 | 0.10247 | 5.91E-08 | 5.13E-07 | up-regulated |
| ATHL1 | 80162 | 3.08281 | 5.71063 | 6.01E-08 | 5.21E-07 | up-regulated |
| LRRC36 | 55282 | 3.31366 | 1.91048 | 6.01E-08 | 5.21E-07 | up-regulated |
| DUSP26 | 78986 | -2.1648 | -1.0581 | 6.13E-08 | 5.32E-07 | down-regulated |
| FCRL3 | 115352 | -2.594 | -0.0582 | 6.31E-08 | 5.46E-07 | down-regulated |
| BGN | 633 | 2.86256 | 8.35572 | 6.35E-08 | 5.49E-07 | up-regulated |
| C1orf114 | 57821 | -2.0515 | -2.3886 | 6.46E-08 | 5.58E-07 | down-regulated |
| DRP2 | 1821 | -2.121 | 0.73828 | 6.69E-08 | 5.76E-07 | down-regulated |
| PBX4 | 80714 | 2.36432 | 1.08752 | 6.70E-08 | 5.76E-07 | up-regulated |
| MGC16121 | 84848 | 3.78633 | -0.3012 | 6.78E-08 | 5.83E-07 | up-regulated |
| PTP4A3 | 11156 | 2.50609 | 6.07404 | 6.79E-08 | 5.83E-07 | up-regulated |
| BTNL8 | 79908 | -2.4744 | 3.8108 | 6.83E-08 | 5.86E-07 | down-regulated |
| STC1 | 6781 | 2.25684 | 4.26492 | 6.90E-08 | 5.91E-07 | up-regulated |
| SLC5A11 | 115584 | -2.3615 | -2.2051 | 7.14E-08 | 6.10E-07 | down-regulated |
| TH | 7054 | 3.90135 | 0.22454 | 7.17E-08 | 6.12E-07 | up-regulated |
| PLAC8 | 51316 | -2.413 | 5.73003 | 7.24E-08 | 6.17E-07 | down-regulated |
| LOC84931 | 84931 | 7.00904 | -1.4309 | 7.28E-08 | 6.20E-07 | up-regulated |
| CAPN13 | 92291 | -2.0669 | 3.42913 | 7.37E-08 | 6.27E-07 | down-regulated |
| CKB | 1152 | -2.0057 | 9.37994 | 7.44E-08 | 6.32E-07 | down-regulated |
| UBE2QL1 | 134111 | -2.1891 | 1.02561 | 7.46E-08 | 6.34E-07 | down-regulated |
| FCRL2 | 79368 | -2.5974 | -0.3067 | 7.48E-08 | 6.35E-07 | down-regulated |
| CPXM1 | 56265 | 2.63669 | 3.95405 | 7.61E-08 | 6.45E-07 | up-regulated |
| KLK7 | 5650 | 8.67191 | 1.80984 | 7.66E-08 | 6.49E-07 | up-regulated |
| CXCL11 | 6373 | 3.84272 | 3.50097 | 7.77E-08 | 6.58E-07 | up-regulated |
| MYOM3 | 127294 | 2.72796 | 4.94801 | 7.91E-08 | 6.68E-07 | up-regulated |
| IL11 | 3589 | 4.87786 | 2.19322 | 7.98E-08 | 6.73E-07 | up-regulated |
| ABCA6 | 23460 | -2.6597 | 1.25216 | 8.19E-08 | 6.88E-07 | down-regulated |
| C1orf105 | 92346 | 5.81513 | -1.7571 | 8.21E-08 | 6.89E-07 | up-regulated |
| GPR143 | 4935 | 3.10862 | 3.06671 | 8.22E-08 | 6.90E-07 | up-regulated |
| CYP24A1 | 1591 | 7.47899 | 1.05945 | 8.25E-08 | 6.92E-07 | up-regulated |
| SIGLEC6 | 946 | -2.2632 | -1.5396 | 8.26E-08 | 6.92E-07 | down-regulated |
| BMP5 | 653 | -2.354 | 0.7505 | 8.48E-08 | 7.10E-07 | down-regulated |
| NPY2R | 4887 | -3.7547 | -1.6857 | 8.56E-08 | 7.16E-07 | down-regulated |
| CLEC5A | 23601 | 3.53537 | 1.47404 | 8.60E-08 | 7.18E-07 | up-regulated |
| PRSS22 | 64063 | 2.59298 | 4.309 | 8.77E-08 | 7.31E-07 | up-regulated |
| COL27A1 | 85301 | 2.89707 | 3.99885 | 8.78E-08 | 7.32E-07 | up-regulated |
| C4orf7 | 260436 | -3.4438 | 1.46976 | 8.89E-08 | 7.40E-07 | down-regulated |
| CCL21 | 6366 | -2.4537 | 4.84565 | 8.99E-08 | 7.48E-07 | down-regulated |
| MYBL2 | 4605 | 2.14158 | 7.09016 | 9.31E-08 | 7.73E-07 | up-regulated |
| CCL28 | 56477 | -2.0446 | 3.64839 | 9.52E-08 | 7.89E-07 | down-regulated |
| SPTBN5 | 51332 | 3.92476 | 2.59187 | 9.71E-08 | 8.03E-07 | up-regulated |
| C13orf29 | 283487 | 2.23009 | 2.32785 | 9.86E-08 | 8.14E-07 | up-regulated |
| TNFRSF6B | 8771 | 4.38909 | 5.41034 | 1.01E-07 | 8.31E-07 | up-regulated |
| DTNA | 1837 | -2.0261 | 2.84916 | 1.02E-07 | 8.40E-07 | down-regulated |
| C20orf165 | 128497 | 2.35358 | -0.122 | 1.03E-07 | 8.46E-07 | up-regulated |
| AQP4 | 361 | -3.3792 | -2.8616 | 1.11E-07 | 9.08E-07 | down-regulated |
| DMBX1 | 127343 | 7.03798 | -0.0166 | 1.15E-07 | 9.41E-07 | up-regulated |
| KRT20 | 54474 | -2.0783 | 8.84606 | 1.18E-07 | 9.61E-07 | down-regulated |
| IL23A | 51561 | 2.8014 | 1.33231 | 1.20E-07 | 9.70E-07 | up-regulated |
| LTC4S | 4056 | -2.0023 | -1.2041 | 1.21E-07 | 9.79E-07 | down-regulated |
| FAM3B | 54097 | 6.04458 | 4.19049 | 1.22E-07 | 9.89E-07 | up-regulated |
| SLCO1B3 | 28234 | 7.31127 | 3.13176 | 1.24E-07 | 1.00E-06 | up-regulated |
| TAS2R38 | 5726 | 3.83428 | -1.5055 | 1.34E-07 | 1.07E-06 | up-regulated |
| KIF5C | 3800 | -2.2086 | 1.97316 | 1.37E-07 | 1.10E-06 | down-regulated |
| KIF26B | 55083 | 3.35508 | 3.05201 | 1.43E-07 | 1.14E-06 | up-regulated |
| NEK5 | 341676 | 3.84435 | -0.3775 | 1.43E-07 | 1.14E-06 | up-regulated |
| FAM5C | 339479 | -3.2795 | 0.52186 | 1.44E-07 | 1.15E-06 | down-regulated |
| C1orf180 | 439927 | 4.12323 | -0.211 | 1.49E-07 | 1.18E-06 | up-regulated |
| FAM55D | 54827 | -2.6798 | 5.5953 | 1.50E-07 | 1.19E-06 | down-regulated |
| OLR1 | 4973 | 4.14847 | 2.3908 | 1.50E-07 | 1.19E-06 | up-regulated |
| SIGLEC8 | 27181 | -2.241 | -0.0504 | 1.53E-07 | 1.21E-06 | down-regulated |
| TACSTD2 | 4070 | 5.55748 | 5.81816 | 1.53E-07 | 1.22E-06 | up-regulated |
| HTR1D | 3352 | 3.43638 | 3.1075 | 1.56E-07 | 1.23E-06 | up-regulated |
| PTGDR | 5729 | -2.7286 | 1.41831 | 1.57E-07 | 1.24E-06 | down-regulated |
| LEF1 | 51176 | 2.39414 | 4.14238 | 1.57E-07 | 1.24E-06 | up-regulated |
| UCA1 | 652995 | 4.66363 | 4.99621 | 1.65E-07 | 1.30E-06 | up-regulated |
| C13orf38 | 728591 | -3.0544 | -2.7603 | 1.67E-07 | 1.31E-06 | down-regulated |
| GALNT13 | 114805 | -2.4871 | -1.8979 | 1.72E-07 | 1.35E-06 | down-regulated |
| UBD | 10537 | 3.21051 | 6.70814 | 1.76E-07 | 1.38E-06 | up-regulated |
| TM6SF2 | 53345 | -2.0567 | 0.62066 | 1.76E-07 | 1.38E-06 | down-regulated |
| HAGHL | 84264 | 3.55216 | 2.65543 | 1.79E-07 | 1.41E-06 | up-regulated |
| CILP | 8483 | -2.6073 | 3.37473 | 1.80E-07 | 1.42E-06 | down-regulated |
| MSX1 | 4487 | 3.26578 | 2.86516 | 1.86E-07 | 1.46E-06 | up-regulated |
| ELMOD1 | 55531 | -2.2016 | -2.8309 | 2.02E-07 | 1.57E-06 | down-regulated |
| GAD1 | 2571 | 4.74201 | 0.92631 | 2.03E-07 | 1.57E-06 | up-regulated |
| ONECUT2 | 9480 | 3.16047 | 2.22711 | 2.03E-07 | 1.58E-06 | up-regulated |
| IGSF5 | 150084 | -2.1595 | -2.4892 | 2.04E-07 | 1.58E-06 | down-regulated |
| DYNC1I1 | 1780 | -2.4716 | 1.36341 | 2.05E-07 | 1.59E-06 | down-regulated |
| C8orf51 | 78998 | 2.15792 | 0.82291 | 2.10E-07 | 1.62E-06 | up-regulated |
| MMP13 | 4322 | 6.65005 | 1.66352 | 2.11E-07 | 1.63E-06 | up-regulated |
| GCNT3 | 9245 | -2.1021 | 6.64334 | 2.13E-07 | 1.65E-06 | down-regulated |
| SYT5 | 6861 | -2.3434 | -1.4785 | 2.24E-07 | 1.73E-06 | down-regulated |
| STK32A | 202374 | -2.8137 | -2.8029 | 2.27E-07 | 1.74E-06 | down-regulated |
| HOXD13 | 3239 | -2.3357 | 3.04808 | 2.27E-07 | 1.74E-06 | down-regulated |
| FRMD3 | 257019 | -2.137 | 1.74601 | 2.31E-07 | 1.77E-06 | down-regulated |
| PRDM12 | 59335 | 2.09934 | -1.4964 | 2.33E-07 | 1.78E-06 | up-regulated |
| CEACAM7 | 1087 | -2.6849 | 8.61544 | 2.41E-07 | 1.84E-06 | down-regulated |
| PDPN | 10630 | 2.21752 | 4.79438 | 2.42E-07 | 1.84E-06 | up-regulated |
| NAP1L6 | 645996 | -2.5545 | -3.0319 | 2.45E-07 | 1.86E-06 | down-regulated |
| ZBTB7C | 201501 | -2.0794 | 4.41717 | 2.66E-07 | 2.01E-06 | down-regulated |
| THSD1P1 | 374500 | 2.08326 | 4.07038 | 2.76E-07 | 2.07E-06 | up-regulated |
| PRSS33 | 260429 | 6.04893 | 3.5331 | 2.76E-07 | 2.07E-06 | up-regulated |
| SPINK5 | 11005 | -2.2213 | 3.41031 | 2.99E-07 | 2.23E-06 | down-regulated |
| SLC6A20 | 54716 | 3.76997 | 4.83907 | 3.10E-07 | 2.31E-06 | up-regulated |
| RIMS4 | 140730 | -2.7543 | -0.0695 | 3.11E-07 | 2.31E-06 | down-regulated |
| TPSG1 | 25823 | -2.6316 | 2.66076 | 3.38E-07 | 2.48E-06 | down-regulated |
| COL1A1 | 1277 | 2.92305 | 11.446 | 3.45E-07 | 2.53E-06 | up-regulated |
| GPT | 2875 | -2.3499 | 3.79982 | 3.73E-07 | 2.72E-06 | down-regulated |
| AGT | 183 | 3.47851 | 5.64616 | 4.05E-07 | 2.93E-06 | up-regulated |
| SMPX | 23676 | -3.1669 | 0.65765 | 4.10E-07 | 2.96E-06 | down-regulated |
| PTPRN | 5798 | -2.0669 | 0.73776 | 4.21E-07 | 3.03E-06 | down-regulated |
| DIO2 | 1734 | 2.56562 | 4.2706 | 4.25E-07 | 3.06E-06 | up-regulated |
| ESPNP | 284729 | 4.80581 | -2.5119 | 4.30E-07 | 3.09E-06 | up-regulated |
| ASPHD1 | 253982 | 2.53878 | 3.01152 | 4.37E-07 | 3.14E-06 | up-regulated |
| SPDYC | 387778 | 4.95941 | -0.7635 | 4.40E-07 | 3.16E-06 | up-regulated |
| MATN4 | 8785 | -2.2283 | -2.8639 | 4.44E-07 | 3.18E-06 | down-regulated |
| ASXL3 | 80816 | -2.0897 | -0.1109 | 4.67E-07 | 3.33E-06 | down-regulated |
| F13A1 | 2162 | -2.1549 | 4.63567 | 4.75E-07 | 3.38E-06 | down-regulated |
| TMEM211 | 255349 | 4.91765 | 1.10707 | 4.97E-07 | 3.53E-06 | up-regulated |
| POU2AF1 | 5450 | -2.1455 | 3.38718 | 5.00E-07 | 3.55E-06 | down-regulated |
| SLC13A2 | 9058 | -2.5739 | 3.75622 | 5.16E-07 | 3.65E-06 | down-regulated |
| CYP2D7P1 | 1564 | 3.14981 | 0.60172 | 5.46E-07 | 3.83E-06 | up-regulated |
| CORIN | 10699 | 4.04489 | 1.58546 | 5.67E-07 | 3.97E-06 | up-regulated |
| ADAMTS6 | 11174 | 2.11932 | 1.1756 | 5.77E-07 | 4.04E-06 | up-regulated |
| HRASLS2 | 54979 | -2.2834 | 0.68503 | 5.91E-07 | 4.12E-06 | down-regulated |
| MYEOV | 26579 | 3.38269 | 4.60189 | 5.94E-07 | 4.14E-06 | up-regulated |
| SIX1 | 6495 | 4.17615 | 0.25601 | 6.06E-07 | 4.22E-06 | up-regulated |
| C8orf73 | 642475 | 2.45823 | 4.56951 | 6.15E-07 | 4.28E-06 | up-regulated |
| DSG1 | 1828 | 3.06974 | -0.8406 | 6.17E-07 | 4.29E-06 | up-regulated |
| FLJ35024 | 401491 | -2.1842 | -2.091 | 6.36E-07 | 4.41E-06 | down-regulated |
| MS4A2 | 2206 | -2.1001 | 0.74198 | 6.64E-07 | 4.60E-06 | down-regulated |
| GABRG2 | 2566 | -3.1953 | -2.1181 | 6.66E-07 | 4.60E-06 | down-regulated |
| PCDH10 | 57575 | -2.6448 | -1.3812 | 6.67E-07 | 4.61E-06 | down-regulated |
| NNAT | 4826 | -2.4422 | 2.16594 | 6.67E-07 | 4.61E-06 | down-regulated |
| CNTFR | 1271 | -2.8631 | 0.62459 | 6.87E-07 | 4.74E-06 | down-regulated |
| SLC13A3 | 64849 | 4.30797 | 3.33277 | 7.05E-07 | 4.85E-06 | up-regulated |
| PAH | 5053 | 7.05036 | 2.81041 | 7.11E-07 | 4.88E-06 | up-regulated |
| CLVS2 | 134829 | -3.2168 | -3.0427 | 7.33E-07 | 5.02E-06 | down-regulated |
| SOX2OT | 347689 | -2.3037 | -2.9465 | 7.38E-07 | 5.05E-06 | down-regulated |
| PHLDA1 | 22822 | 2.09199 | 7.45539 | 7.48E-07 | 5.10E-06 | up-regulated |
| REG1A | 5967 | 9.42913 | 7.99463 | 7.49E-07 | 5.11E-06 | up-regulated |
| C19orf45 | 374877 | 2.35642 | 1.02871 | 7.57E-07 | 5.16E-06 | up-regulated |
| UCHL1 | 7345 | -2.1176 | 2.94257 | 7.66E-07 | 5.22E-06 | down-regulated |
| CXCL1 | 2919 | 2.64805 | 6.11143 | 7.73E-07 | 5.26E-06 | up-regulated |
| RORB | 6096 | -2.3091 | -2.7244 | 7.94E-07 | 5.39E-06 | down-regulated |
| CYP4B1 | 1580 | -2.4175 | -1.5947 | 8.07E-07 | 5.47E-06 | down-regulated |
| RAET1K | 646024 | 3.11471 | -1.6338 | 8.07E-07 | 5.47E-06 | up-regulated |
| FAM53A | 152877 | 2.02479 | 0.72096 | 8.44E-07 | 5.71E-06 | up-regulated |
| RELN | 5649 | -2.5337 | 1.54756 | 9.09E-07 | 6.13E-06 | down-regulated |
| PODNL1 | 79883 | 2.17701 | 2.37737 | 9.16E-07 | 6.17E-06 | up-regulated |
| C8orf80 | 389643 | -2.4179 | 0.26979 | 9.34E-07 | 6.28E-06 | down-regulated |
| CD300LG | 146894 | -3.0376 | -1.4857 | 9.35E-07 | 6.29E-06 | down-regulated |
| LRAT | 9227 | -2.9119 | -0.9628 | 9.46E-07 | 6.35E-06 | down-regulated |
| ADRB3 | 155 | -2.6069 | -2.5569 | 9.49E-07 | 6.37E-06 | down-regulated |
| GBA3 | 57733 | -2.9691 | 0.7376 | 9.53E-07 | 6.40E-06 | down-regulated |
| LRRC16B | 90668 | 2.52445 | 1.30291 | 9.56E-07 | 6.41E-06 | up-regulated |
| CETN4P | 729338 | -2.106 | -3.5133 | 9.87E-07 | 6.60E-06 | down-regulated |
| C9orf43 | 257169 | 2.3404 | -0.7055 | 1.05E-06 | 6.95E-06 | up-regulated |
| CAMKV | 79012 | 5.44144 | 1.11286 | 1.05E-06 | 6.98E-06 | up-regulated |
| C10orf81 | 79949 | 2.38819 | 5.31583 | 1.07E-06 | 7.11E-06 | up-regulated |
| MSX2 | 4488 | 4.0667 | 3.3732 | 1.08E-06 | 7.17E-06 | up-regulated |
| GJB4 | 127534 | 3.13668 | 2.41271 | 1.13E-06 | 7.50E-06 | up-regulated |
| KLC3 | 147700 | 3.50427 | 0.19984 | 1.18E-06 | 7.73E-06 | up-regulated |
| SYN2 | 6854 | -2.3485 | -0.294 | 1.22E-06 | 8.01E-06 | down-regulated |
| CLEC4F | 165530 | -2.178 | -2.2179 | 1.25E-06 | 8.17E-06 | down-regulated |
| FAM163B | 642968 | -2.5824 | -2.6284 | 1.26E-06 | 8.24E-06 | down-regulated |
| TRPM2 | 7226 | 2.18335 | 4.69609 | 1.26E-06 | 8.25E-06 | up-regulated |
| DCAF12L2 | 340578 | -2.6798 | -2.9904 | 1.26E-06 | 8.25E-06 | down-regulated |
| ADAMTS12 | 81792 | 3.24876 | 3.35717 | 1.28E-06 | 8.36E-06 | up-regulated |
| GBX2 | 2637 | 5.46598 | -1.5618 | 1.29E-06 | 8.37E-06 | up-regulated |
| ISM2 | 145501 | 5.85491 | 2.82845 | 1.29E-06 | 8.40E-06 | up-regulated |
| SFTPA1 | 653509 | -3.8846 | -1.7558 | 1.30E-06 | 8.42E-06 | down-regulated |
| LY6G6C | 80740 | 3.96507 | 0.76768 | 1.30E-06 | 8.42E-06 | up-regulated |
| LOC100133161 | 100133161 | 2.05456 | 0.72031 | 1.32E-06 | 8.53E-06 | up-regulated |
| AR | 367 | -2.3033 | -0.6587 | 1.35E-06 | 8.71E-06 | down-regulated |
| CR1 | 1378 | -2.0832 | 1.74362 | 1.35E-06 | 8.74E-06 | down-regulated |
| HSPB7 | 27129 | -2.2372 | 3.64781 | 1.36E-06 | 8.79E-06 | down-regulated |
| INHBB | 3625 | 3.23716 | 3.914 | 1.55E-06 | 9.89E-06 | up-regulated |
| REG3A | 5068 | 10.6381 | 6.65569 | 1.55E-06 | 9.90E-06 | up-regulated |
| DMRTA1 | 63951 | -2.4462 | -1.1881 | 1.56E-06 | 9.93E-06 | down-regulated |
| SOX14 | 8403 | 7.35221 | -1.107 | 1.57E-06 | 9.96E-06 | up-regulated |
| NEB | 4703 | 3.59248 | 1.0793 | 1.57E-06 | 9.96E-06 | up-regulated |
| HSF4 | 3299 | 2.50192 | 2.76955 | 1.60E-06 | 1.02E-05 | up-regulated |
| TFR2 | 7036 | 5.12695 | 3.45059 | 1.60E-06 | 1.02E-05 | up-regulated |
| KRT6A | 3853 | 7.76595 | 2.0707 | 1.61E-06 | 1.02E-05 | up-regulated |
| KRT6C | 286887 | 6.5139 | -0.0642 | 1.65E-06 | 1.04E-05 | up-regulated |
| CCDC160 | 347475 | -2.733 | -3.6133 | 1.66E-06 | 1.05E-05 | down-regulated |
| TNNI3 | 7137 | 5.47758 | 0.60874 | 1.67E-06 | 1.05E-05 | up-regulated |
| EPYC | 1833 | 9.41572 | 1.75048 | 1.68E-06 | 1.06E-05 | up-regulated |
| ALAS2 | 212 | -2.5193 | -3.1484 | 1.68E-06 | 1.06E-05 | down-regulated |
| VWC2 | 375567 | -2.1027 | -2.8945 | 1.71E-06 | 1.08E-05 | down-regulated |
| BFSP2 | 8419 | -2.3045 | -2.8783 | 1.76E-06 | 1.11E-05 | down-regulated |
| UGT1A7 | 54577 | -3.4876 | -3.3159 | 1.80E-06 | 1.13E-05 | down-regulated |
| DSG3 | 1830 | 6.3826 | 4.61367 | 1.90E-06 | 1.19E-05 | up-regulated |
| ZFR2 | 23217 | -2.0954 | -3.563 | 1.94E-06 | 1.21E-05 | down-regulated |
| ZNF280A | 129025 | 6.40661 | -1.9309 | 1.98E-06 | 1.23E-05 | up-regulated |
| FAP | 2191 | 2.93425 | 4.32282 | 2.00E-06 | 1.24E-05 | up-regulated |
| LPO | 4025 | 6.93922 | 0.22588 | 2.10E-06 | 1.30E-05 | up-regulated |
| DNAH2 | 146754 | 2.80239 | 1.50459 | 2.12E-06 | 1.31E-05 | up-regulated |
| PMFBP1 | 83449 | 2.29177 | 1.49455 | 2.14E-06 | 1.32E-05 | up-regulated |
| AGTR1 | 185 | -2.7428 | 1.67137 | 2.16E-06 | 1.33E-05 | down-regulated |
| CHRNA3 | 1136 | -2.089 | 1.75698 | 2.18E-06 | 1.35E-05 | down-regulated |
| WDR72 | 256764 | 4.48508 | 4.06901 | 2.19E-06 | 1.35E-05 | up-regulated |
| STAB2 | 55576 | -2.262 | -0.7247 | 2.20E-06 | 1.36E-05 | down-regulated |
| UNC93A | 54346 | 3.63987 | 1.60139 | 2.24E-06 | 1.38E-05 | up-regulated |
| WT1 | 7490 | 5.19586 | 1.30749 | 2.26E-06 | 1.39E-05 | up-regulated |
| SNHG4 | 724102 | 2.1106 | 0.9257 | 2.26E-06 | 1.39E-05 | up-regulated |
| C5orf46 | 389336 | 5.48219 | -1.0918 | 2.30E-06 | 1.41E-05 | up-regulated |
| IGFL4 | 444882 | 4.9144 | -2.0675 | 2.37E-06 | 1.45E-05 | up-regulated |
| SEMA3D | 223117 | -2.2583 | 1.46661 | 2.39E-06 | 1.46E-05 | down-regulated |
| DSC3 | 1825 | 4.89762 | 4.23202 | 2.42E-06 | 1.48E-05 | up-regulated |
| THBS4 | 7060 | -2.8701 | 4.70037 | 2.46E-06 | 1.49E-05 | down-regulated |
| DHRS2 | 10202 | 4.54003 | 2.37147 | 2.52E-06 | 1.53E-05 | up-regulated |
| ABCC2 | 1244 | 4.6855 | 2.85397 | 2.63E-06 | 1.58E-05 | up-regulated |
| EN2 | 2020 | 5.62071 | 0.11137 | 2.66E-06 | 1.60E-05 | up-regulated |
| RNF165 | 494470 | -2.0739 | -1.8878 | 2.70E-06 | 1.62E-05 | down-regulated |
| POU3F4 | 5456 | -3.2678 | -3.7698 | 2.72E-06 | 1.63E-05 | down-regulated |
| NRAP | 4892 | -2.9107 | -0.7459 | 2.73E-06 | 1.64E-05 | down-regulated |
| BMP7 | 655 | 2.93933 | 5.85969 | 2.76E-06 | 1.65E-05 | up-regulated |
| FEZF1 | 389549 | 8.04394 | 0.96858 | 2.82E-06 | 1.69E-05 | up-regulated |
| ATP13A4 | 84239 | -2.4418 | 0.51636 | 2.84E-06 | 1.70E-05 | down-regulated |
| ACBD7 | 414149 | 2.22416 | 2.12135 | 2.93E-06 | 1.75E-05 | up-regulated |
| SLC17A9 | 63910 | 2.07947 | 5.64628 | 2.98E-06 | 1.78E-05 | up-regulated |
| CLDN9 | 9080 | 3.12396 | 1.23582 | 3.02E-06 | 1.80E-05 | up-regulated |
| LZTS1 | 11178 | 2.06357 | 2.79741 | 3.07E-06 | 1.82E-05 | up-regulated |
| MAPK15 | 225689 | 4.24989 | 1.84314 | 3.15E-06 | 1.87E-05 | up-regulated |
| OSR1 | 130497 | -2.1954 | 1.11858 | 3.29E-06 | 1.94E-05 | down-regulated |
| LILRA4 | 23547 | -2.2249 | -1.8824 | 3.31E-06 | 1.95E-05 | down-regulated |
| SRMS | 6725 | 3.05359 | 1.16977 | 3.33E-06 | 1.96E-05 | up-regulated |
| CXCL3 | 2921 | 2.29303 | 4.98879 | 3.37E-06 | 1.98E-05 | up-regulated |
| GTF2A1L | 11036 | -2.2332 | -1.8716 | 3.44E-06 | 2.03E-05 | down-regulated |
| MAP7D2 | 256714 | 3.27445 | 4.04729 | 3.45E-06 | 2.03E-05 | up-regulated |
| C6orf105 | 84830 | -2.2376 | 4.00124 | 3.56E-06 | 2.08E-05 | down-regulated |
| LRRC4C | 57689 | -2.1296 | -0.872 | 3.58E-06 | 2.09E-05 | down-regulated |
| SLIT2 | 9353 | -2.1475 | 2.55886 | 3.60E-06 | 2.10E-05 | down-regulated |
| DKK4 | 27121 | 8.39593 | 2.1852 | 3.63E-06 | 2.11E-05 | up-regulated |
| COL9A3 | 1299 | 3.43562 | 4.73504 | 3.82E-06 | 2.21E-05 | up-regulated |
| UGT2B15 | 7366 | -2.7537 | 4.6215 | 3.84E-06 | 2.22E-05 | down-regulated |
| PDZD7 | 79955 | 2.49123 | -0.3176 | 3.86E-06 | 2.24E-05 | up-regulated |
| GDF15 | 9518 | 2.01427 | 7.1836 | 3.89E-06 | 2.25E-05 | up-regulated |
| ALDH3B2 | 222 | 3.84202 | 0.99192 | 4.09E-06 | 2.36E-05 | up-regulated |
| TAS1R1 | 80835 | -2.4038 | -2.7937 | 4.09E-06 | 2.36E-05 | down-regulated |
| PNLIPRP2 | 5408 | -2.5023 | 2.21097 | 4.11E-06 | 2.37E-05 | down-regulated |
| EXTL1 | 2134 | -2.1144 | -2.0337 | 4.30E-06 | 2.47E-05 | down-regulated |
| B3GALT5 | 10317 | -2.2068 | 3.47638 | 4.30E-06 | 2.47E-05 | down-regulated |
| HEPHL1 | 341208 | 6.71762 | 1.53318 | 4.44E-06 | 2.55E-05 | up-regulated |
| WNT7B | 7477 | 4.5968 | 0.14099 | 4.55E-06 | 2.61E-05 | up-regulated |
| GZMB | 3002 | 2.71922 | 3.32053 | 4.65E-06 | 2.66E-05 | up-regulated |
| C2orf48 | 348738 | 2.88377 | -0.2372 | 4.84E-06 | 2.75E-05 | up-regulated |
| HPCAL4 | 51440 | -2.2114 | -1.9444 | 4.85E-06 | 2.76E-05 | down-regulated |
| AK5 | 26289 | -2.2288 | -0.3289 | 5.07E-06 | 2.87E-05 | down-regulated |
| BEST2 | 54831 | -3.0654 | 2.32961 | 5.23E-06 | 2.95E-05 | down-regulated |
| SGCA | 6442 | -2.2471 | 1.4455 | 5.31E-06 | 2.99E-05 | down-regulated |
| TM4SF19 | 116211 | 3.60524 | -0.3939 | 5.53E-06 | 3.10E-05 | up-regulated |
| HRASLS5 | 117245 | -2.2497 | -0.186 | 5.57E-06 | 3.12E-05 | down-regulated |
| CALN1 | 83698 | -2.6185 | -2.8054 | 5.60E-06 | 3.13E-05 | down-regulated |
| WNT8B | 7479 | 3.61608 | -2.5212 | 5.72E-06 | 3.19E-05 | up-regulated |
| ADAMTS14 | 140766 | 2.06709 | 3.34913 | 5.81E-06 | 3.24E-05 | up-regulated |
| VIP | 7432 | -2.5014 | 4.00876 | 5.98E-06 | 3.32E-05 | down-regulated |
| KRT7 | 3855 | 3.39124 | 2.96474 | 6.13E-06 | 3.39E-05 | up-regulated |
| CAV3 | 859 | -3.185 | -3.8464 | 6.13E-06 | 3.39E-05 | down-regulated |
| TCN1 | 6947 | 7.19653 | 4.44999 | 6.18E-06 | 3.42E-05 | up-regulated |
| KIAA0125 | 9834 | -2.1015 | 0.67107 | 6.26E-06 | 3.46E-05 | down-regulated |
| SEC14L4 | 284904 | 5.90329 | -0.1377 | 6.30E-06 | 3.48E-05 | up-regulated |
| CADPS | 8618 | 2.5382 | 4.43191 | 6.32E-06 | 3.49E-05 | up-regulated |
| LRRC7 | 57554 | -2.799 | -2.3436 | 6.37E-06 | 3.51E-05 | down-regulated |
| PCSK9 | 255738 | 2.40664 | 5.99217 | 6.42E-06 | 3.54E-05 | up-regulated |
| KISS1 | 3814 | 3.36792 | -0.3403 | 6.51E-06 | 3.58E-05 | up-regulated |
| GRIN2B | 2904 | 2.59894 | 0.37291 | 6.52E-06 | 3.58E-05 | up-regulated |
| NXPH4 | 11247 | 4.49968 | 1.72137 | 6.56E-06 | 3.60E-05 | up-regulated |
| TMPRSS5 | 80975 | 4.66481 | 1.15822 | 6.74E-06 | 3.69E-05 | up-regulated |
| SLC35D3 | 340146 | 3.98687 | 2.9705 | 6.76E-06 | 3.70E-05 | up-regulated |
| GRB7 | 2886 | 2.99481 | 6.61779 | 6.81E-06 | 3.72E-05 | up-regulated |
| REP15 | 387849 | -2.2775 | 3.25298 | 6.89E-06 | 3.76E-05 | down-regulated |
| MMP8 | 4317 | 5.09443 | -1.2642 | 7.05E-06 | 3.84E-05 | up-regulated |
| SLAMF9 | 89886 | 3.55982 | -1.1229 | 7.12E-06 | 3.87E-05 | up-regulated |
| TBX15 | 6913 | 3.56208 | 0.4677 | 7.16E-06 | 3.89E-05 | up-regulated |
| IRX5 | 10265 | 3.64467 | 0.9971 | 7.20E-06 | 3.91E-05 | up-regulated |
| IBSP | 3381 | 6.45358 | 1.16702 | 7.41E-06 | 4.02E-05 | up-regulated |
| KRT16 | 3868 | 5.68859 | 0.6357 | 7.42E-06 | 4.02E-05 | up-regulated |
| GLYATL3 | 389396 | -3.1952 | -3.2092 | 7.46E-06 | 4.04E-05 | down-regulated |
| NANOS3 | 342977 | 3.92065 | 0.5445 | 7.54E-06 | 4.08E-05 | up-regulated |
| NOX4 | 50507 | 2.99716 | 1.51857 | 7.59E-06 | 4.11E-05 | up-regulated |
| EDAR | 10913 | 3.25497 | 4.62231 | 7.67E-06 | 4.14E-05 | up-regulated |
| PRRT4 | 401399 | -2.0597 | -1.9125 | 7.89E-06 | 4.25E-05 | down-regulated |
| PLCB4 | 5332 | 2.12808 | 7.55556 | 8.01E-06 | 4.32E-05 | up-regulated |
| MST1 | 4485 | 3.25149 | 3.14033 | 8.37E-06 | 4.49E-05 | up-regulated |
| CPA4 | 51200 | 3.6178 | -1.3994 | 8.39E-06 | 4.50E-05 | up-regulated |
| HS6ST3 | 266722 | -2.4972 | -2.2399 | 8.41E-06 | 4.51E-05 | down-regulated |
| C1orf182 | 128229 | 2.07115 | -2.0134 | 9.00E-06 | 4.79E-05 | up-regulated |
| UGT2B11 | 10720 | -3.236 | -3.4095 | 9.03E-06 | 4.80E-05 | down-regulated |
| CCDC19 | 25790 | 2.17171 | -0.2463 | 9.11E-06 | 4.84E-05 | up-regulated |
| DCX | 1641 | -2.3952 | -2.5889 | 9.17E-06 | 4.86E-05 | down-regulated |
| DUSP27 | 92235 | 6.83329 | 5.92361 | 9.40E-06 | 4.98E-05 | up-regulated |
| HTR3E | 285242 | -2.6414 | -2.5659 | 9.59E-06 | 5.06E-05 | down-regulated |
| SLC6A4 | 6532 | 4.17782 | 2.44328 | 9.75E-06 | 5.13E-05 | up-regulated |
| PNPLA1 | 285848 | 2.73531 | -0.1625 | 9.78E-06 | 5.15E-05 | up-regulated |
| KRTAP5-4 | 387267 | 3.95751 | -2.7995 | 1.01E-05 | 5.29E-05 | up-regulated |
| SLC11A1 | 6556 | 2.55298 | 2.73105 | 1.01E-05 | 5.29E-05 | up-regulated |
| COL4A6 | 1288 | -2.0112 | 0.60901 | 1.01E-05 | 5.29E-05 | down-regulated |
| SLITRK3 | 22865 | -2.9197 | -0.1285 | 1.01E-05 | 5.30E-05 | down-regulated |
| TRPV4 | 59341 | 2.27792 | 1.13894 | 1.02E-05 | 5.34E-05 | up-regulated |
| ZIC2 | 7546 | 6.19268 | 2.44981 | 1.03E-05 | 5.36E-05 | up-regulated |
| CAMP | 820 | -2.2098 | -3.274 | 1.03E-05 | 5.38E-05 | down-regulated |
| PRELP | 5549 | -2.0283 | 5.32364 | 1.04E-05 | 5.41E-05 | down-regulated |
| WISP1 | 8840 | 2.37316 | 2.7909 | 1.10E-05 | 5.67E-05 | up-regulated |
| GRAMD2 | 196996 | 2.15246 | 2.81534 | 1.12E-05 | 5.77E-05 | up-regulated |
| FOXI2 | 399823 | -2.0592 | -3.0919 | 1.13E-05 | 5.81E-05 | down-regulated |
| AGR3 | 155465 | -2.031 | 5.48262 | 1.15E-05 | 5.95E-05 | down-regulated |
| PAX5 | 5079 | -2.511 | 0.04836 | 1.18E-05 | 6.07E-05 | down-regulated |
| DNAH5 | 1767 | 2.50234 | -0.0073 | 1.20E-05 | 6.15E-05 | up-regulated |
| CPNE4 | 131034 | -2.524 | -2.5749 | 1.25E-05 | 6.36E-05 | down-regulated |
| LOC554202 | 554202 | 4.88796 | -0.9973 | 1.26E-05 | 6.44E-05 | up-regulated |
| KAL1 | 3730 | 2.71664 | 3.63992 | 1.27E-05 | 6.44E-05 | up-regulated |
| R3HDML | 140902 | 2.22683 | -0.2635 | 1.27E-05 | 6.47E-05 | up-regulated |
| F7 | 2155 | 4.31806 | 1.84705 | 1.30E-05 | 6.62E-05 | up-regulated |
| LOC283867 | 283867 | 3.89163 | -2.7987 | 1.35E-05 | 6.85E-05 | up-regulated |
| IGFL1 | 374918 | 8.22341 | -0.2989 | 1.42E-05 | 7.16E-05 | up-regulated |
| C2orf61 | 285051 | 2.76647 | -2.5482 | 1.43E-05 | 7.21E-05 | up-regulated |
| DUSP9 | 1852 | 3.84795 | -0.7068 | 1.51E-05 | 7.56E-05 | up-regulated |
| GNG4 | 2786 | 2.8977 | 5.85075 | 1.52E-05 | 7.62E-05 | up-regulated |
| PGC | 5225 | 4.57801 | 0.58445 | 1.53E-05 | 7.65E-05 | up-regulated |
| ALG1L | 200810 | 2.65077 | 2.0724 | 1.53E-05 | 7.65E-05 | up-regulated |
| FLJ41941 | 100192420 | -2.1121 | -3.7465 | 1.56E-05 | 7.81E-05 | down-regulated |
| GLRA4 | 441509 | -2.1797 | -2.4183 | 1.60E-05 | 7.98E-05 | down-regulated |
| GP2 | 2813 | -2.8481 | 0.92617 | 1.64E-05 | 8.16E-05 | down-regulated |
| CLDN16 | 10686 | 3.11935 | -1.1045 | 1.69E-05 | 8.37E-05 | up-regulated |
| OMD | 4958 | -2.4711 | 1.37448 | 1.70E-05 | 8.45E-05 | down-regulated |
| ZNF469 | 84627 | 2.09263 | 3.24742 | 1.70E-05 | 8.45E-05 | up-regulated |
| APOBEC1 | 339 | 2.26707 | 2.18632 | 1.73E-05 | 8.57E-05 | up-regulated |
| PRKAA2 | 5563 | -2.0783 | 2.55041 | 1.85E-05 | 9.12E-05 | down-regulated |
| REEP6 | 92840 | 2.21069 | 4.85951 | 1.89E-05 | 9.30E-05 | up-regulated |
| ITGA11 | 22801 | 2.2378 | 5.11021 | 1.90E-05 | 9.33E-05 | up-regulated |
| PRDM13 | 59336 | 3.81255 | -1.219 | 1.91E-05 | 9.36E-05 | up-regulated |
| GPR120 | 338557 | -2.0259 | 2.78782 | 1.93E-05 | 9.45E-05 | down-regulated |
| BMP4 | 652 | 2.15099 | 6.62837 | 1.94E-05 | 9.50E-05 | up-regulated |
| SNCB | 6620 | -2.2282 | -3.3456 | 1.96E-05 | 9.57E-05 | down-regulated |
| PLA2G4E | 123745 | 2.47455 | -2.1936 | 1.97E-05 | 9.62E-05 | up-regulated |
| LOC728606 | 728606 | -2.6479 | -2.4478 | 1.99E-05 | 9.73E-05 | down-regulated |
| SH2D5 | 400745 | 3.4499 | -0.7677 | 2.10E-05 | 0.000102 | up-regulated |
| TLX1NB | 100038246 | 3.92109 | -2.7478 | 2.11E-05 | 0.000102 | up-regulated |
| SLC18A3 | 6572 | -2.7347 | -2.4825 | 2.11E-05 | 0.000102 | down-regulated |
| NPFFR1 | 64106 | 2.57108 | -1.9794 | 2.17E-05 | 0.000105 | up-regulated |
| LOC150197 | 150197 | 2.7273 | -0.5615 | 2.18E-05 | 0.000105 | up-regulated |
| PALM3 | 342979 | 3.41118 | 2.17827 | 2.20E-05 | 0.000106 | up-regulated |
| IGF2BP1 | 10642 | 6.58963 | 3.05942 | 2.30E-05 | 0.00011 | up-regulated |
| GLYATL1 | 92292 | 4.03254 | 1.9385 | 2.38E-05 | 0.000113 | up-regulated |
| KRT9 | 3857 | -2.3124 | -3.5084 | 2.38E-05 | 0.000113 | down-regulated |
| ATAD3C | 219293 | 2.733 | 2.116 | 2.41E-05 | 0.000115 | up-regulated |
| ALDOB | 229 | 4.86287 | 6.035 | 2.45E-05 | 0.000117 | up-regulated |
| ADAMTS18 | 170692 | 2.49722 | -1.1927 | 2.46E-05 | 0.000117 | up-regulated |
| DLX6 | 1750 | 4.62574 | 0.72402 | 2.54E-05 | 0.00012 | up-regulated |
| RP1 | 6101 | 4.43356 | -1.6843 | 2.58E-05 | 0.000122 | up-regulated |
| KIAA1875 | 340390 | 3.19803 | 0.51645 | 2.68E-05 | 0.000126 | up-regulated |
| LOC284837 | 284837 | 2.06477 | 0.74045 | 2.71E-05 | 0.000127 | up-regulated |
| FGA | 2243 | 13.53 | 8.34963 | 2.75E-05 | 0.000129 | up-regulated |
| POU4F1 | 5457 | 2.79518 | -0.4447 | 2.76E-05 | 0.000129 | up-regulated |
| RAD51AP2 | 729475 | 2.54752 | -1.4472 | 2.79E-05 | 0.000131 | up-regulated |
| SCARNA12 | 677777 | 2.05023 | -1.1931 | 2.82E-05 | 0.000132 | up-regulated |
| COL28A1 | 340267 | -2.0391 | 1.53891 | 2.84E-05 | 0.000133 | down-regulated |
| PPEF1 | 5475 | 2.72394 | -1.3834 | 2.89E-05 | 0.000135 | up-regulated |
| CXCL2 | 2920 | 2.09708 | 4.54257 | 2.95E-05 | 0.000137 | up-regulated |
| CFB | 629 | 2.22176 | 7.75191 | 3.01E-05 | 0.00014 | up-regulated |
| B4GALNT4 | 338707 | 3.32216 | 3.55148 | 3.02E-05 | 0.00014 | up-regulated |
| CHGB | 1114 | -2.2497 | 2.55934 | 3.08E-05 | 0.000143 | down-regulated |
| ASGR1 | 432 | 3.90282 | 3.17927 | 3.08E-05 | 0.000143 | up-regulated |
| PPBP | 5473 | 8.0738 | 4.19122 | 3.20E-05 | 0.000148 | up-regulated |
| MYH16 | 84176 | 2.57606 | -2.9543 | 3.22E-05 | 0.000148 | up-regulated |
| CCL19 | 6363 | -2.2861 | 2.75528 | 3.37E-05 | 0.000155 | down-regulated |
| C9orf169 | 375791 | 3.15961 | -0.2953 | 3.38E-05 | 0.000155 | up-regulated |
| CHI3L2 | 1117 | -2.0042 | 0.33947 | 3.45E-05 | 0.000158 | down-regulated |
| GDF1 | 2657 | -2.2823 | -2.8257 | 3.46E-05 | 0.000159 | down-regulated |
| SSTR5 | 6755 | 3.7295 | -0.0078 | 3.52E-05 | 0.000161 | up-regulated |
| LOC285954 | 285954 | 2.78275 | -3.0931 | 3.60E-05 | 0.000164 | up-regulated |
| ZNF727 | 442319 | -2.1588 | -2.5687 | 3.69E-05 | 0.000168 | down-regulated |
| DLX3 | 1747 | 4.03751 | 1.26957 | 3.76E-05 | 0.000171 | up-regulated |
| MAT1A | 4143 | 6.33543 | 4.19157 | 3.91E-05 | 0.000177 | up-regulated |
| VENTX | 27287 | 4.29294 | 2.68229 | 3.99E-05 | 0.00018 | up-regulated |
| HABP2 | 3026 | 8.89489 | 3.07847 | 4.00E-05 | 0.00018 | up-regulated |
| CSF2 | 1437 | 3.69552 | -0.8826 | 4.02E-05 | 0.000181 | up-regulated |
| ? | 728788 | -2.0767 | -3.734 | 4.13E-05 | 0.000185 | down-regulated |
| FAM189A1 | 23359 | -2.0429 | 2.19407 | 4.36E-05 | 0.000195 | down-regulated |
| HTR4 | 3360 | -2.0672 | -0.323 | 4.39E-05 | 0.000196 | down-regulated |
| CSTL1 | 128817 | 4.77837 | -3.1516 | 4.46E-05 | 0.000199 | up-regulated |
| WNT11 | 7481 | 2.8691 | 4.75373 | 4.47E-05 | 0.000199 | up-regulated |
| RFX8 | 731220 | 3.0104 | -2.796 | 4.70E-05 | 0.000209 | up-regulated |
| CNTN3 | 5067 | -2.0681 | 1.20723 | 4.80E-05 | 0.000212 | down-regulated |
| PSORS1C2 | 170680 | 3.13596 | -0.9367 | 4.91E-05 | 0.000217 | up-regulated |
| LRRN4 | 164312 | 2.98008 | -1.4655 | 4.96E-05 | 0.000219 | up-regulated |
| DLX6AS | 285987 | 4.26139 | -0.7462 | 4.97E-05 | 0.000219 | up-regulated |
| FAM70B | 348013 | 2.01829 | 2.25026 | 4.97E-05 | 0.000219 | up-regulated |
| PROZ | 8858 | 3.66608 | -1.1421 | 5.18E-05 | 0.000227 | up-regulated |
| C20orf195 | 79025 | 2.03749 | -0.6923 | 5.34E-05 | 0.000234 | up-regulated |
| ERP27 | 121506 | 3.33288 | 3.30584 | 5.41E-05 | 0.000236 | up-regulated |
| ANKRD33 | 341405 | 3.74044 | -2.8689 | 5.44E-05 | 0.000237 | up-regulated |
| WFDC10B | 280664 | 3.73921 | -1.7164 | 5.45E-05 | 0.000238 | up-regulated |
| CLDN14 | 23562 | 2.9745 | 0.42859 | 5.46E-05 | 0.000238 | up-regulated |
| PRKCG | 5582 | 4.47146 | 1.09145 | 5.62E-05 | 0.000244 | up-regulated |
| MYLPF | 29895 | 3.56348 | -2.4699 | 5.67E-05 | 0.000246 | up-regulated |
| CDKN2A | 1029 | 2.41775 | 2.49444 | 5.86E-05 | 0.000254 | up-regulated |
| LOC440925 | 440925 | 2.19285 | 1.66701 | 5.95E-05 | 0.000257 | up-regulated |
| PTPRZ1 | 5803 | -2.668 | 0.60892 | 5.99E-05 | 0.000259 | down-regulated |
| BAAT | 570 | 6.67642 | 2.20576 | 6.05E-05 | 0.000261 | up-regulated |
| DIRC1 | 116093 | 4.41238 | -3.3533 | 6.20E-05 | 0.000267 | up-regulated |
| NCRNA00105 | 80161 | 2.12581 | 1.98342 | 6.21E-05 | 0.000267 | up-regulated |
| RDH16 | 8608 | 5.24495 | 1.50095 | 6.29E-05 | 0.00027 | up-regulated |
| SPP1 | 6696 | 3.6733 | 7.36878 | 6.34E-05 | 0.000272 | up-regulated |
| FGB | 2244 | 13.3174 | 7.90822 | 6.52E-05 | 0.000279 | up-regulated |
| MYBPHL | 343263 | 7.86101 | -0.6399 | 7.06E-05 | 0.0003 | up-regulated |
| SYN3 | 8224 | 3.21944 | 1.88153 | 7.09E-05 | 0.000301 | up-regulated |
| AGAP7 | 653268 | 2.48946 | -0.365 | 7.11E-05 | 0.000302 | up-regulated |
| LOC100192378 | 100192378 | -2.0203 | -2.985 | 7.14E-05 | 0.000303 | down-regulated |
| SPACA3 | 124912 | 4.04015 | 0.06618 | 7.22E-05 | 0.000306 | up-regulated |
| BRSK2 | 9024 | 3.17037 | 2.41515 | 7.25E-05 | 0.000307 | up-regulated |
| ORM1 | 5004 | 13.1091 | 7.81813 | 7.27E-05 | 0.000308 | up-regulated |
| FCN3 | 8547 | 3.0616 | 0.67625 | 7.28E-05 | 0.000308 | up-regulated |
| RSPH10B2 | 728194 | 2.24103 | -2.6169 | 7.30E-05 | 0.000309 | up-regulated |
| GJC3 | 349149 | 2.73876 | -1.1708 | 7.51E-05 | 0.000317 | up-regulated |
| APCDD1 | 147495 | 2.66272 | 7.22401 | 7.59E-05 | 0.000319 | up-regulated |
| MATN3 | 4148 | 3.4086 | 2.72274 | 7.60E-05 | 0.00032 | up-regulated |
| F2 | 2147 | 10.3909 | 4.01927 | 7.68E-05 | 0.000323 | up-regulated |
| MMP10 | 4319 | 3.72358 | 2.52562 | 7.68E-05 | 0.000323 | up-regulated |
| SPRR1A | 6698 | 5.33401 | -2.1772 | 7.75E-05 | 0.000326 | up-regulated |
| GRM8 | 2918 | 2.33051 | 3.96141 | 7.75E-05 | 0.000326 | up-regulated |
| MSLN | 10232 | 3.22438 | 6.24469 | 7.78E-05 | 0.000327 | up-regulated |
| FAM157A | 728262 | 2.33607 | -0.5931 | 7.93E-05 | 0.000332 | up-regulated |
| ZIC5 | 85416 | 7.95831 | 1.29695 | 7.94E-05 | 0.000333 | up-regulated |
| GNGT1 | 2792 | 4.82359 | -2.4848 | 8.02E-05 | 0.000335 | up-regulated |
| OR51E1 | 143503 | 2.26696 | 2.2613 | 8.11E-05 | 0.000339 | up-regulated |
| PAEP | 5047 | 5.07723 | -1.9214 | 8.59E-05 | 0.000357 | up-regulated |
| FOLR1 | 2348 | 4.66809 | 3.22008 | 8.64E-05 | 0.000359 | up-regulated |
| SLC38A3 | 10991 | 5.33979 | 2.11209 | 8.92E-05 | 0.000369 | up-regulated |
| PCDH15 | 65217 | -2.2474 | -3.5589 | 8.97E-05 | 0.000371 | down-regulated |
| GIF | 2694 | 4.06236 | 0.36709 | 9.00E-05 | 0.000372 | up-regulated |
| GNG13 | 51764 | -2.2071 | -2.3289 | 9.07E-05 | 0.000374 | down-regulated |
| PLAC1 | 10761 | 3.35771 | -0.8497 | 9.24E-05 | 0.000381 | up-regulated |
| H19 | 283120 | 3.98101 | 8.39645 | 9.34E-05 | 0.000385 | up-regulated |
| DEFA6 | 1671 | 6.34674 | 4.90729 | 9.57E-05 | 0.000392 | up-regulated |
| DHDPSL | 112817 | 3.13166 | 0.34447 | 9.76E-05 | 0.0004 | up-regulated |
| LOC440356 | 440356 | 4.44979 | -2.4088 | 9.77E-05 | 0.0004 | up-regulated |
| DNASE1L2 | 1775 | 2.00438 | -0.6433 | 0.0001 | 0.000409 | up-regulated |
| ELF5 | 2001 | 6.18255 | 1.60604 | 0.0001 | 0.00041 | up-regulated |
| SLC34A3 | 142680 | 3.27107 | -0.6855 | 0.000102 | 0.000418 | up-regulated |
| CRTAC1 | 55118 | -2.2104 | -0.0676 | 0.000103 | 0.000421 | down-regulated |
| PCP2 | 126006 | 3.19154 | -2 | 0.000106 | 0.00043 | up-regulated |
| FLJ45445 | 399844 | 2.25469 | 1.53926 | 0.000106 | 0.000431 | up-regulated |
| AKR1B15 | 441282 | -2.3345 | -2.9954 | 0.000108 | 0.000437 | down-regulated |
| TP73 | 7161 | 2.04143 | 1.38662 | 0.00011 | 0.000446 | up-regulated |
| LCN12 | 286256 | 2.16005 | 1.53851 | 0.000111 | 0.000449 | up-regulated |
| NRADDP | 100129354 | 2.33609 | -2.6427 | 0.000113 | 0.000456 | up-regulated |
| GAS2 | 2620 | 2.07733 | 1.5226 | 0.000114 | 0.000458 | up-regulated |
| TPRXL | 348825 | 3.86421 | -0.0014 | 0.000115 | 0.000462 | up-regulated |
| PLA2G2D | 26279 | -2.0159 | 1.40595 | 0.000115 | 0.000462 | down-regulated |
| WBSCR28 | 135886 | 2.45054 | -1.7838 | 0.000116 | 0.000466 | up-regulated |
| CILP2 | 148113 | 2.89868 | 1.52767 | 0.000116 | 0.000467 | up-regulated |
| C19orf51 | 352909 | 2.44603 | -0.2244 | 0.000116 | 0.000467 | up-regulated |
| ORM2 | 5005 | 12.1956 | 4.85238 | 0.000117 | 0.00047 | up-regulated |
| WISP3 | 8838 | 6.93685 | 1.42421 | 0.00012 | 0.000481 | up-regulated |
| NPC1L1 | 29881 | 4.41071 | 2.8055 | 0.000121 | 0.000482 | up-regulated |
| C21orf29 | 54084 | 3.30758 | 0.47983 | 0.000121 | 0.000482 | up-regulated |
| DUSP15 | 128853 | 2.60158 | 1.39193 | 0.000121 | 0.000484 | up-regulated |
| SERPINF2 | 5345 | 3.66188 | 4.5005 | 0.000125 | 0.0005 | up-regulated |
| C9orf70 | 84850 | 5.61914 | -2.5734 | 0.000126 | 0.000502 | up-regulated |
| RP1L1 | 94137 | 2.09971 | -1.4913 | 0.000127 | 0.000506 | up-regulated |
| LOC221442 | 221442 | 2.36977 | 1.24653 | 0.000128 | 0.000507 | up-regulated |
| COL22A1 | 169044 | 3.48966 | 1.44827 | 0.00013 | 0.000515 | up-regulated |
| NCRNA00114 | 400866 | 2.43648 | -0.4036 | 0.000131 | 0.000519 | up-regulated |
| P4HA3 | 283208 | 2.26964 | 1.6251 | 0.000131 | 0.000521 | up-regulated |
| GPR81 | 27198 | 4.19747 | 1.19508 | 0.000132 | 0.000523 | up-regulated |
| SFRP5 | 6425 | -2.6628 | 0.3109 | 0.000132 | 0.000523 | down-regulated |
| MME | 4311 | 3.0064 | 5.41043 | 0.000132 | 0.000523 | up-regulated |
| CACNG4 | 27092 | 3.55232 | 2.73661 | 0.000134 | 0.00053 | up-regulated |
| LBP | 3929 | 8.90708 | 4.43061 | 0.000138 | 0.000542 | up-regulated |
| PRSS41 | 360226 | 4.53739 | -2.1382 | 0.000139 | 0.000546 | up-regulated |
| SP8 | 221833 | 5.77421 | -0.4487 | 0.00014 | 0.000548 | up-regulated |
| UPK2 | 7379 | 6.5592 | -0.2455 | 0.000143 | 0.000559 | up-regulated |
| REG1B | 5968 | 7.63481 | 4.7465 | 0.000145 | 0.000568 | up-regulated |
| PLAC4 | 191585 | 3.25357 | 0.56461 | 0.000147 | 0.000572 | up-regulated |
| GJA3 | 2700 | 2.58449 | -0.931 | 0.000148 | 0.000579 | up-regulated |
| APOC2 | 344 | 3.85046 | 4.62228 | 0.000149 | 0.00058 | up-regulated |
| GSDMA | 284110 | 2.2144 | 1.18706 | 0.00015 | 0.000585 | up-regulated |
| HOXC11 | 3227 | 7.02849 | -0.1126 | 0.00015 | 0.000585 | up-regulated |
| AKAP4 | 8852 | 5.7045 | -0.9518 | 0.000153 | 0.000596 | up-regulated |
| AAA1 | 404744 | 4.69463 | -3.1889 | 0.000158 | 0.000614 | up-regulated |
| SLC30A2 | 7780 | 3.06795 | 2.11075 | 0.000162 | 0.000625 | up-regulated |
| GPR27 | 2850 | -2.1234 | -2.9427 | 0.000162 | 0.000627 | down-regulated |
| OSM | 5008 | 2.42433 | 2.06989 | 0.000168 | 0.000645 | up-regulated |
| IL8 | 3576 | 2.78366 | 6.37721 | 0.000168 | 0.000647 | up-regulated |
| WSCD2 | 9671 | -2.1774 | -0.1728 | 0.000169 | 0.000648 | down-regulated |
| SFRP4 | 6424 | 3.23439 | 6.07875 | 0.000171 | 0.000657 | up-regulated |
| HEPACAM2 | 253012 | -2.4537 | 5.07429 | 0.000172 | 0.00066 | down-regulated |
| KREMEN2 | 79412 | 2.48104 | 1.20612 | 0.000173 | 0.000661 | up-regulated |
| KCNH4 | 23415 | 2.92662 | -0.5368 | 0.000173 | 0.000662 | up-regulated |
| AMBP | 259 | 7.67499 | 5.85702 | 0.000177 | 0.000676 | up-regulated |
| C14orf105 | 55195 | 6.51658 | -1.1719 | 0.000181 | 0.00069 | up-regulated |
| ADH1B | 125 | -2.688 | 5.97866 | 0.000185 | 0.000702 | down-regulated |
| HOTAIR | 100124700 | 7.32683 | -1.1175 | 0.000188 | 0.000714 | up-regulated |
| DKFZp566F0947 | 94023 | -2.0101 | -3.6514 | 0.000189 | 0.000717 | down-regulated |
| PLIN1 | 5346 | -2.18 | 1.69832 | 0.000192 | 0.000727 | down-regulated |
| SIX2 | 10736 | 3.4032 | 0.67613 | 0.000194 | 0.000733 | up-regulated |
| CHRNA7 | 1139 | -2.1769 | -0.8289 | 0.000196 | 0.000739 | down-regulated |
| HPX | 3263 | 8.10914 | 5.8934 | 0.0002 | 0.000753 | up-regulated |
| PRAME | 23532 | 4.707 | 1.65621 | 0.0002 | 0.000755 | up-regulated |
| PNPLA3 | 80339 | 4.31702 | -0.0198 | 0.000202 | 0.000759 | up-regulated |
| MYH7B | 57644 | 4.01 | 3.41716 | 0.000213 | 0.000798 | up-regulated |
| PPAPDC1A | 196051 | 2.85501 | 1.23083 | 0.000217 | 0.00081 | up-regulated |
| C4BPB | 725 | 2.33497 | 2.80081 | 0.000218 | 0.000813 | up-regulated |
| NEUROG2 | 63973 | 6.06786 | -0.9405 | 0.000218 | 0.000814 | up-regulated |
| TNNT2 | 7139 | 3.42016 | -0.8425 | 0.000222 | 0.000827 | up-regulated |
| KRTAP4-1 | 85285 | 2.57573 | -0.2988 | 0.000224 | 0.000833 | up-regulated |
| UCN | 7349 | 2.29962 | -1.9457 | 0.000225 | 0.000836 | up-regulated |
| SERPINE1 | 5054 | 2.05446 | 5.79334 | 0.000228 | 0.000847 | up-regulated |
| NXPH1 | 30010 | 4.08353 | -2.6077 | 0.000229 | 0.000849 | up-regulated |
| TCL6 | 27004 | -2.321 | -1.9579 | 0.000233 | 0.000863 | down-regulated |
| LRRC15 | 131578 | 2.25232 | 4.2425 | 0.000234 | 0.000867 | up-regulated |
| DUSP5P | 574029 | 3.64115 | -2.0218 | 0.000236 | 0.000873 | up-regulated |
| KIAA1984 | 84960 | 2.11858 | 2.32406 | 0.000238 | 0.00088 | up-regulated |
| FABP4 | 2167 | -2.4014 | 2.55612 | 0.000245 | 0.000904 | down-regulated |
| SPAG17 | 200162 | 3.16635 | -2.6331 | 0.000246 | 0.000905 | up-regulated |
| CYP26A1 | 1592 | 4.80208 | -0.5383 | 0.000247 | 0.00091 | up-regulated |
| SLC26A9 | 115019 | 6.45484 | 0.92213 | 0.000252 | 0.000926 | up-regulated |
| SAA4 | 6291 | 8.85163 | 2.34901 | 0.000253 | 0.000929 | up-regulated |
| TSPO2 | 222642 | 2.60527 | -1.489 | 0.000254 | 0.000934 | up-regulated |
| THBS2 | 7058 | 2.43235 | 7.59817 | 0.00026 | 0.000951 | up-regulated |
| NCRNA00176 | 284739 | 3.41463 | 1.35721 | 0.000261 | 0.000955 | up-regulated |
| LOC389493 | 389493 | -2.6854 | -2.996 | 0.000262 | 0.000957 | down-regulated |
| CCDC114 | 93233 | 2.00437 | -1.1355 | 0.000265 | 0.000968 | up-regulated |
| ALK | 238 | -2.2775 | -2.0853 | 0.000266 | 0.000971 | down-regulated |
| AADAC | 13 | 5.3929 | 1.59289 | 0.000267 | 0.000975 | up-regulated |
| GOLGA6L6 | 727832 | 5.66848 | -1.2746 | 0.00027 | 0.000985 | up-regulated |
| SAA2 | 6289 | 6.20892 | 5.51409 | 0.000277 | 0.001007 | up-regulated |
| SLC7A9 | 11136 | 4.63405 | -0.029 | 0.000278 | 0.001012 | up-regulated |
| ADAMTS2 | 9509 | 2.063 | 4.18682 | 0.000284 | 0.00103 | up-regulated |
| HIST1H1E | 3008 | 2.45203 | -1.1243 | 0.000287 | 0.001041 | up-regulated |
| FER1L6 | 654463 | -2.3763 | 3.02181 | 0.000291 | 0.001053 | down-regulated |
| DEFA5 | 1670 | 7.25052 | 5.63357 | 0.000291 | 0.001053 | up-regulated |
| RASSF10 | 644943 | 2.06553 | 1.98078 | 0.000294 | 0.001063 | up-regulated |
| PCP4 | 5121 | -2.015 | 2.59395 | 0.000302 | 0.00109 | down-regulated |
| CABP4 | 57010 | 2.3515 | -0.5463 | 0.000315 | 0.00113 | up-regulated |
| SERPIND1 | 3053 | 5.49098 | 2.96817 | 0.000318 | 0.001141 | up-regulated |
| CPNE9 | 151835 | 2.40631 | -1.3014 | 0.000319 | 0.001142 | up-regulated |
| AMH | 268 | 3.08281 | -0.4477 | 0.000322 | 0.001153 | up-regulated |
| SMTNL2 | 342527 | 2.53063 | 1.18408 | 0.000323 | 0.001154 | up-regulated |
| RNF182 | 221687 | 3.72363 | 1.41201 | 0.000326 | 0.001166 | up-regulated |
| SPRR1B | 6699 | 5.82139 | -1.324 | 0.000331 | 0.001181 | up-regulated |
| ZNF750 | 79755 | 4.16437 | 0.33581 | 0.000334 | 0.00119 | up-regulated |
| ADRA2C | 152 | 2.58756 | 2.7727 | 0.000335 | 0.001193 | up-regulated |
| RHBG | 57127 | 5.09937 | -2.9431 | 0.000336 | 0.001195 | up-regulated |
| ERBB4 | 2066 | -2.0085 | -3.0655 | 0.000342 | 0.001213 | down-regulated |
| DRD2 | 1813 | 3.3992 | 2.54264 | 0.000348 | 0.001232 | up-regulated |
| MRGPRX2 | 117194 | -3.0465 | -3.7039 | 0.000352 | 0.001242 | down-regulated |
| DPF1 | 8193 | 2.63087 | -1.5145 | 0.000354 | 0.00125 | up-regulated |
| LGR6 | 59352 | 2.38807 | 4.80644 | 0.000356 | 0.001257 | up-regulated |
| IL17A | 3605 | 3.65041 | -0.9314 | 0.000357 | 0.00126 | up-regulated |
| CYP2W1 | 54905 | 2.73988 | 5.56584 | 0.00036 | 0.001266 | up-regulated |
| C7orf52 | 375607 | 4.32429 | -1.0231 | 0.000365 | 0.001284 | up-regulated |
| AHSG | 197 | 10.5229 | 4.97672 | 0.000366 | 0.001288 | up-regulated |
| SIX4 | 51804 | 2.19331 | 0.82695 | 0.000367 | 0.001289 | up-regulated |
| C9orf57 | 138240 | 3.51938 | -3.082 | 0.000367 | 0.00129 | up-regulated |
| FMO3 | 2328 | 3.59074 | 2.09855 | 0.000368 | 0.001295 | up-regulated |
| FAM131C | 348487 | 2.45489 | -0.6686 | 0.000369 | 0.001295 | up-regulated |
| FCGBP | 8857 | -2.1859 | 10.1742 | 0.000371 | 0.001302 | down-regulated |
| FGGY | 55277 | 2.22569 | 4.91732 | 0.000376 | 0.001319 | up-regulated |
| C4orf48 | 401115 | 2.51444 | 3.13256 | 0.000377 | 0.00132 | up-regulated |
| HS6ST2 | 90161 | 3.01697 | 4.01748 | 0.000377 | 0.001321 | up-regulated |
| PADI3 | 51702 | 4.98085 | 1.5256 | 0.000381 | 0.001334 | up-regulated |
| MAGEA3 | 4102 | 11.6122 | 3.80599 | 0.000389 | 0.001359 | up-regulated |
| FGF20 | 26281 | 6.52235 | -0.1243 | 0.000397 | 0.001381 | up-regulated |
| TAL2 | 6887 | 2.44255 | -3.2987 | 0.000399 | 0.001387 | up-regulated |
| C1QTNF8 | 390664 | 3.54143 | -3.3449 | 0.000424 | 0.001466 | up-regulated |
| IL24 | 11009 | 4.0804 | 1.60676 | 0.000424 | 0.001468 | up-regulated |
| C1QL4 | 338761 | 3.46366 | -2.6918 | 0.00043 | 0.001484 | up-regulated |
| HULC | 728655 | 9.3986 | 3.89825 | 0.000443 | 0.001524 | up-regulated |
| C19orf59 | 199675 | 2.83033 | -0.2275 | 0.000446 | 0.001533 | up-regulated |
| AKNAD1 | 254268 | 2.34412 | -1.1148 | 0.000454 | 0.001557 | up-regulated |
| SOX1 | 6656 | 5.1567 | 1.7271 | 0.000457 | 0.001565 | up-regulated |
| SLC1A7 | 6512 | 2.7815 | 3.13905 | 0.000462 | 0.001583 | up-regulated |
| BEAN | 146227 | 2.65281 | 0.17682 | 0.000469 | 0.001603 | up-regulated |
| IGF2BP3 | 10643 | 3.76446 | 3.19034 | 0.00047 | 0.001606 | up-regulated |
| ABCA13 | 154664 | 2.45805 | 0.72817 | 0.000473 | 0.001616 | up-regulated |
| TBX20 | 57057 | 4.22133 | -1.4078 | 0.000473 | 0.001616 | up-regulated |
| CKMT2 | 1160 | 3.45513 | 3.66404 | 0.000474 | 0.001619 | up-regulated |
| TRY6 | 154754 | 7.46902 | 0.65827 | 0.000477 | 0.001627 | up-regulated |
| HSPB3 | 8988 | -2.2206 | -0.7651 | 0.00048 | 0.001637 | down-regulated |
| CXCL10 | 3627 | 2.12646 | 4.24745 | 0.000482 | 0.001643 | up-regulated |
| S100A5 | 6276 | 2.82149 | -2.0471 | 0.000494 | 0.001681 | up-regulated |
| CCR8 | 1237 | 2.03463 | -0.8706 | 0.000501 | 0.001701 | up-regulated |
| OBP2B | 29989 | 6.57771 | -1.1098 | 0.000515 | 0.00174 | up-regulated |
| ICAM5 | 7087 | 2.90877 | -0.6774 | 0.000525 | 0.00177 | up-regulated |
| CYP1A1 | 1543 | -2.6555 | -2.1783 | 0.000525 | 0.001771 | down-regulated |
| LOC285401 | 285401 | 3.30081 | -3.197 | 0.000537 | 0.001807 | up-regulated |
| HP | 3240 | 10.9252 | 8.97614 | 0.00054 | 0.001813 | up-regulated |
| GSC | 145258 | 2.48428 | -2.5643 | 0.000547 | 0.001836 | up-regulated |
| LCN2 | 3934 | 2.33712 | 8.99741 | 0.00055 | 0.001846 | up-regulated |
| IGF2 | 3481 | 4.64698 | 10.0274 | 0.000554 | 0.001854 | up-regulated |
| BEST3 | 144453 | 3.50523 | -2.1182 | 0.000557 | 0.001862 | up-regulated |
| CALML3 | 810 | 2.67426 | 0.01857 | 0.000564 | 0.00188 | up-regulated |
| INHBE | 83729 | 3.97467 | 1.60896 | 0.000567 | 0.00189 | up-regulated |
| GPR109B | 8843 | 3.36443 | 1.28661 | 0.000574 | 0.001913 | up-regulated |
| SPRR2D | 6703 | 5.18022 | -1.355 | 0.000576 | 0.001916 | up-regulated |
| PRSS30P | 124221 | 2.93891 | -0.5944 | 0.000576 | 0.001918 | up-regulated |
| ATP6V0A4 | 50617 | 6.27282 | -1.3946 | 0.000577 | 0.00192 | up-regulated |
| VSIG8 | 391123 | 4.02342 | -1.2809 | 0.00058 | 0.001927 | up-regulated |
| USP26 | 83844 | 3.69698 | -3.7138 | 0.000581 | 0.00193 | up-regulated |
| KRTAP3-1 | 83896 | 6.73654 | -1.6455 | 0.000581 | 0.001932 | up-regulated |
| C9orf71 | 169693 | 3.70344 | 0.44551 | 0.000593 | 0.001963 | up-regulated |
| C19orf30 | 284424 | -2.3969 | -3.8485 | 0.000596 | 0.001972 | down-regulated |
| IL1F5 | 26525 | 4.23703 | -1.7608 | 0.000597 | 0.001975 | up-regulated |
| MPP4 | 58538 | 2.2787 | -3.3647 | 0.000599 | 0.001983 | up-regulated |
| PIWIL1 | 9271 | 2.89024 | 1.49749 | 0.000607 | 0.002003 | up-regulated |
| B4GALNT2 | 124872 | -2.5153 | 1.39638 | 0.000609 | 0.002009 | down-regulated |
| FZD10 | 11211 | 4.21097 | 3.6997 | 0.000622 | 0.002045 | up-regulated |
| PDIA2 | 64714 | 2.48823 | 0.33641 | 0.000637 | 0.002092 | up-regulated |
| APOA1 | 335 | 8.74732 | 5.99791 | 0.000642 | 0.002105 | up-regulated |
| FAM83A | 84985 | 2.6065 | 0.05165 | 0.000642 | 0.002105 | up-regulated |
| SULT1E1 | 6783 | 5.67881 | 1.32006 | 0.000644 | 0.00211 | up-regulated |
| NTSR1 | 4923 | -2.0154 | 1.82157 | 0.000645 | 0.002113 | down-regulated |
| C12orf70 | 341346 | 2.1968 | -2.912 | 0.000651 | 0.002129 | up-regulated |
| TM7SF4 | 81501 | 2.95034 | -1.1476 | 0.000655 | 0.002141 | up-regulated |
| CLCA1 | 1179 | -2.6202 | 7.90527 | 0.000666 | 0.002171 | down-regulated |
| TNNT1 | 7138 | 4.57884 | 1.21578 | 0.000684 | 0.002223 | up-regulated |
| VTCN1 | 79679 | 4.94355 | -0.8177 | 0.000692 | 0.002245 | up-regulated |
| DLL3 | 10683 | 3.01484 | -0.9004 | 0.000702 | 0.002272 | up-regulated |
| HSPA6 | 3310 | 2.69352 | 3.94946 | 0.000703 | 0.002276 | up-regulated |
| VTN | 7448 | 8.39869 | 6.13485 | 0.000707 | 0.002287 | up-regulated |
| FBXO2 | 26232 | 2.59156 | 3.41298 | 0.000708 | 0.002287 | up-regulated |
| DUOXA2 | 405753 | 3.09809 | 5.10718 | 0.000714 | 0.002306 | up-regulated |
| SCG3 | 29106 | -2.0946 | 0.55728 | 0.000714 | 0.002306 | down-regulated |
| KCNJ15 | 3772 | 2.14366 | 0.51249 | 0.000716 | 0.002312 | up-regulated |
| CXCL17 | 284340 | 5.89057 | 1.08711 | 0.00072 | 0.002321 | up-regulated |
| CYP2E1 | 1571 | 6.32284 | 4.84153 | 0.00073 | 0.002352 | up-regulated |
| AKR1CL1 | 340811 | 4.47949 | -3.3218 | 0.00073 | 0.002352 | up-regulated |
| CYP4X1 | 260293 | 2.64945 | 3.18628 | 0.000732 | 0.002357 | up-regulated |
| CALCA | 796 | 3.49811 | 0.25995 | 0.000733 | 0.002359 | up-regulated |
| CFC1B | 653275 | -2.3327 | -2.6718 | 0.000747 | 0.002401 | down-regulated |
| RASL10B | 91608 | 2.12149 | 1.71001 | 0.000756 | 0.002426 | up-regulated |
| COL6A4P2 | 646300 | 2.47451 | -2.2676 | 0.000762 | 0.002444 | up-regulated |
| LOC100130386 | 100130386 | 2.84433 | -2.7035 | 0.000764 | 0.002448 | up-regulated |
| LST-3TM12 | 338821 | 5.11614 | -2.3365 | 0.00079 | 0.002525 | up-regulated |
| SLC22A1 | 6580 | 5.57031 | 1.76229 | 0.000795 | 0.002538 | up-regulated |
| C4BPA | 722 | 4.128 | 4.34921 | 0.000796 | 0.002541 | up-regulated |
| CSMD2 | 114784 | 2.02117 | 0.38471 | 0.000829 | 0.002632 | up-regulated |
| SPRR2A | 6700 | 4.93277 | -1.3632 | 0.000855 | 0.002704 | up-regulated |
| SAA1 | 6288 | 5.13249 | 6.35997 | 0.000859 | 0.002716 | up-regulated |
| UCN3 | 114131 | -2.0339 | 0.21538 | 0.00086 | 0.002717 | down-regulated |
| CXCL9 | 4283 | 2.01726 | 4.8727 | 0.000863 | 0.002725 | up-regulated |
| SPRR3 | 6707 | 5.69153 | -1.4599 | 0.000864 | 0.002728 | up-regulated |
| NMU | 10874 | 2.76037 | 2.2236 | 0.000868 | 0.002738 | up-regulated |
| TRIM54 | 57159 | 3.74437 | 1.67249 | 0.000869 | 0.002742 | up-regulated |
| NR0B2 | 8431 | 2.1595 | 1.9089 | 0.000903 | 0.002837 | up-regulated |
| ITGBL1 | 9358 | 2.4054 | 3.92308 | 0.000929 | 0.002907 | up-regulated |
| HIST1H2AH | 85235 | 2.41505 | -2.8043 | 0.000931 | 0.002912 | up-regulated |
| VGF | 7425 | 2.6711 | 2.15356 | 0.000935 | 0.002923 | up-regulated |
| SLC13A5 | 284111 | 7.34947 | 2.50796 | 0.000945 | 0.00295 | up-regulated |
| CXCL5 | 6374 | 3.77652 | 5.38272 | 0.000954 | 0.002976 | up-regulated |
| NBPF6 | 653149 | 5.33531 | -1.3483 | 0.000959 | 0.002989 | up-regulated |
| AQP9 | 366 | 2.94281 | 3.13345 | 0.00096 | 0.002991 | up-regulated |
| LOC400696 | 400696 | 3.29942 | -1.7171 | 0.000973 | 0.003027 | up-regulated |
| IGF2AS | 51214 | 4.50493 | -0.2839 | 0.000978 | 0.00304 | up-regulated |
| TNNT3 | 7140 | -2.5494 | -2.7843 | 0.000978 | 0.00304 | down-regulated |
| CPN1 | 1369 | 5.28278 | 1.22142 | 0.00099 | 0.003073 | up-regulated |
| ITIH2 | 3698 | 11.267 | 4.28263 | 0.000991 | 0.003073 | up-regulated |
| UMODL1 | 89766 | 2.28113 | 0.06356 | 0.000992 | 0.003077 | up-regulated |
| STRC | 161497 | 2.11744 | -0.9651 | 0.000997 | 0.003091 | up-regulated |
| TTC16 | 158248 | 2.13568 | -1.8993 | 0.000999 | 0.003094 | up-regulated |
| TREM1 | 54210 | 2.14894 | 1.45596 | 0.001008 | 0.003116 | up-regulated |
| CHST4 | 10164 | 4.73373 | 1.02581 | 0.001009 | 0.003119 | up-regulated |
| KRT31 | 3881 | 8.39627 | -0.1259 | 0.001013 | 0.003129 | up-regulated |
| VGLL1 | 51442 | 6.10673 | -1.4951 | 0.001038 | 0.003198 | up-regulated |
| CATSPER1 | 117144 | 2.25433 | -1.8223 | 0.001045 | 0.003218 | up-regulated |
| NXF2 | 56001 | 9.8674 | 1.29081 | 0.00105 | 0.003229 | up-regulated |
| F5 | 2153 | 3.03081 | 4.00969 | 0.001055 | 0.003243 | up-regulated |
| CST5 | 1473 | 4.82134 | -2.1998 | 0.001056 | 0.003245 | up-regulated |
| LOC285629 | 285629 | 2.54646 | -1.6134 | 0.001068 | 0.003276 | up-regulated |
| NPSR1 | 387129 | 6.88938 | 1.70221 | 0.001079 | 0.003304 | up-regulated |
| CITED1 | 4435 | 2.446 | 0.57404 | 0.001103 | 0.00337 | up-regulated |
| SERPINC1 | 462 | 8.76288 | 4.55035 | 0.001104 | 0.003373 | up-regulated |
| C14orf68 | 283600 | 5.61396 | 1.82912 | 0.001126 | 0.003433 | up-regulated |
| CLCN1 | 1180 | 2.42769 | -1.2864 | 0.001139 | 0.003463 | up-regulated |
| HPN | 3249 | 4.10883 | 3.04862 | 0.001142 | 0.003474 | up-regulated |
| MS4A15 | 219995 | 3.04359 | -0.7704 | 0.001148 | 0.003487 | up-regulated |
| PLA2G4D | 283748 | 3.20329 | 0.79265 | 0.001153 | 0.0035 | up-regulated |
| MAGEA5 | 4104 | 2.58401 | -3.1457 | 0.001176 | 0.003563 | up-regulated |
| ARHGAP36 | 158763 | -2.0388 | -3.1442 | 0.001183 | 0.003582 | down-regulated |
| SLC6A1 | 6529 | 2.17302 | 0.81833 | 0.00119 | 0.0036 | up-regulated |
| MAGEA12 | 4111 | 9.92417 | 2.69075 | 0.001206 | 0.003643 | up-regulated |
| KRT39 | 390792 | 6.0746 | -0.3894 | 0.00122 | 0.00368 | up-regulated |
| LGALS7B | 653499 | 4.49356 | -1.5877 | 0.001225 | 0.003695 | up-regulated |
| CXCL6 | 6372 | 2.4399 | 2.2666 | 0.00123 | 0.003707 | up-regulated |
| LOC84740 | 84740 | 3.35911 | 1.13935 | 0.001232 | 0.003713 | up-regulated |
| BNIPL | 149428 | 2.2124 | -0.7004 | 0.001234 | 0.003717 | up-regulated |
| EREG | 2069 | 2.2136 | 6.31733 | 0.001255 | 0.003774 | up-regulated |
| SH2D6 | 284948 | -2.0106 | -1.5038 | 0.001255 | 0.003774 | down-regulated |
| C9orf173 | 441476 | 2.45723 | -2.4972 | 0.0013 | 0.00389 | up-regulated |
| COLEC10 | 10584 | 2.83724 | -1.3385 | 0.001331 | 0.003974 | up-regulated |
| KLK11 | 11012 | 2.65615 | 2.50574 | 0.001337 | 0.00399 | up-regulated |
| ALB | 213 | 15.6127 | 10.1961 | 0.001378 | 0.004098 | up-regulated |
| C17orf78 | 284099 | 6.48548 | 0.15863 | 0.001378 | 0.004099 | up-regulated |
| INS-IGF2 | 723961 | 7.05124 | 0.99554 | 0.00138 | 0.004104 | up-regulated |
| SI | 6476 | -2.2788 | 4.74238 | 0.00139 | 0.004129 | down-regulated |
| HAMP | 57817 | 6.75812 | 3.54508 | 0.001451 | 0.004292 | up-regulated |
| FOXD1 | 2297 | 3.28398 | 0.72538 | 0.001453 | 0.004298 | up-regulated |
| IFNG | 3458 | 2.45766 | -1.5862 | 0.00147 | 0.004338 | up-regulated |
| FOXC2 | 2303 | 2.03966 | -0.5889 | 0.001474 | 0.004348 | up-regulated |
| PTF1A | 256297 | 5.52809 | -1.4967 | 0.001489 | 0.004385 | up-regulated |
| ODAM | 54959 | 3.24557 | 2.58379 | 0.001519 | 0.004458 | up-regulated |
| LAIR2 | 3904 | 2.26862 | -1.438 | 0.001522 | 0.004467 | up-regulated |
| TEKT5 | 146279 | 2.98887 | -1.4652 | 0.001523 | 0.004468 | up-regulated |
| NFE2 | 4778 | 2.52817 | 0.8722 | 0.001562 | 0.004571 | up-regulated |
| SLCO1B1 | 10599 | 8.15034 | 1.17282 | 0.001585 | 0.004627 | up-regulated |
| ASPG | 374569 | -2.1349 | 1.42286 | 0.001601 | 0.004666 | down-regulated |
| RDH12 | 145226 | 2.03407 | -1.2073 | 0.001606 | 0.004678 | up-regulated |
| IL13RA2 | 3598 | 2.17077 | 0.37961 | 0.001623 | 0.004718 | up-regulated |
| VIT | 5212 | -2.2241 | -0.0235 | 0.00163 | 0.004737 | down-regulated |
| MYH4 | 4622 | 3.76707 | -0.5735 | 0.001642 | 0.004767 | up-regulated |
| CCL25 | 6370 | 5.63443 | 0.25191 | 0.00168 | 0.004869 | up-regulated |
| TRIM71 | 131405 | 5.88714 | -2.333 | 0.001727 | 0.004986 | up-regulated |
| TCAM1P | 146771 | 4.10786 | -1.4879 | 0.001749 | 0.005038 | up-regulated |
| XIRP1 | 165904 | 2.11542 | -0.2338 | 0.001788 | 0.005142 | up-regulated |
| ITIH4 | 3700 | 5.8951 | 5.33008 | 0.001806 | 0.005188 | up-regulated |
| C3P1 | 388503 | 8.18057 | -0.3528 | 0.001824 | 0.005234 | up-regulated |
| TDO2 | 6999 | 2.85947 | 3.25143 | 0.001856 | 0.00532 | up-regulated |
| TMEM40 | 55287 | 3.95161 | -3.1574 | 0.001876 | 0.005368 | up-regulated |
| CGB5 | 93659 | 5.29185 | -2.8437 | 0.001879 | 0.005376 | up-regulated |
| ACSBG2 | 81616 | 2.08918 | -3.201 | 0.001894 | 0.005413 | up-regulated |
| SMOC1 | 64093 | 2.29222 | 3.69575 | 0.001902 | 0.005431 | up-regulated |
| IFNE | 338376 | 5.04789 | -2.9705 | 0.001927 | 0.005494 | up-regulated |
| IGFBP1 | 3484 | 5.34362 | 1.73167 | 0.001929 | 0.0055 | up-regulated |
| LOC100133545 | 100133545 | 3.34676 | 0.68336 | 0.00196 | 0.005575 | up-regulated |
| FAM157B | 100132403 | 2.55451 | -3.2741 | 0.001962 | 0.005579 | up-regulated |
| CYP2C9 | 1559 | 6.50725 | 3.28779 | 0.001989 | 0.005645 | up-regulated |
| CPS1 | 1373 | 4.32603 | 3.96874 | 0.002017 | 0.005715 | up-regulated |
| CYP17A1 | 1586 | 6.34893 | -1.3401 | 0.00202 | 0.005721 | up-regulated |
| NDST4 | 64579 | -2.2057 | -3.6951 | 0.002042 | 0.00577 | down-regulated |
| CYP4F8 | 11283 | 3.31964 | -1.8705 | 0.002064 | 0.005824 | up-regulated |
| NPW | 283869 | 3.39755 | 1.17924 | 0.002065 | 0.005825 | up-regulated |
| SERPINA10 | 51156 | 3.99411 | 2.84321 | 0.00208 | 0.005863 | up-regulated |
| C15orf54 | 400360 | 2.17043 | -3.28 | 0.002093 | 0.005891 | up-regulated |
| GJB5 | 2709 | 3.17264 | 1.20041 | 0.002197 | 0.00615 | up-regulated |
| MAGEA6 | 4105 | 9.32784 | 4.31298 | 0.002205 | 0.00617 | up-regulated |
| DLX5 | 1749 | 3.84948 | 0.66101 | 0.002221 | 0.006211 | up-regulated |
| IL17F | 112744 | 3.03447 | -2.4072 | 0.002246 | 0.006274 | up-regulated |
| GCKR | 2646 | 6.00489 | -1.1817 | 0.00228 | 0.006354 | up-regulated |
| UGT1A6 | 54578 | 2.33774 | 3.94947 | 0.002284 | 0.006364 | up-regulated |
| UPK1A | 11045 | 4.84668 | -0.897 | 0.002298 | 0.006394 | up-regulated |
| CA6 | 765 | 6.82689 | -1.6029 | 0.002313 | 0.00643 | up-regulated |
| LOC100131551 | 100131551 | 2.25001 | -2.8494 | 0.002319 | 0.006442 | up-regulated |
| EYA1 | 2138 | 3.56346 | 2.21044 | 0.002341 | 0.006499 | up-regulated |
| HRG | 3273 | 13.6604 | 5.05818 | 0.002388 | 0.006606 | up-regulated |
| FGF3 | 2248 | 6.8594 | -0.8611 | 0.002408 | 0.006657 | up-regulated |
| MUC6 | 4588 | 4.24272 | 0.87444 | 0.002415 | 0.006668 | up-regulated |
| C19orf69 | 100170765 | 3.04324 | -1.7217 | 0.002419 | 0.006678 | up-regulated |
| GABRP | 2568 | 3.46835 | 2.54989 | 0.002465 | 0.006789 | up-regulated |
| SERPINB3 | 6317 | 6.50991 | -1.0155 | 0.002466 | 0.006792 | up-regulated |
| ECEL1 | 9427 | 3.28031 | 0.50638 | 0.002482 | 0.006833 | up-regulated |
| HCRT | 3060 | 4.07464 | -3.0721 | 0.002506 | 0.006887 | up-regulated |
| KLK9 | 284366 | 4.39057 | -3.3654 | 0.002551 | 0.006994 | up-regulated |
| TERC | 7012 | 3.1177 | -1.88 | 0.002569 | 0.00704 | up-regulated |
| SYT12 | 91683 | 2.22117 | 0.25714 | 0.00257 | 0.007041 | up-regulated |
| SERPINA7 | 6906 | 5.07466 | 1.58915 | 0.002617 | 0.007155 | up-regulated |
| CGB | 1082 | 4.32143 | -2.9108 | 0.002638 | 0.007206 | up-regulated |
| MSLNL | 401827 | 4.18733 | -3.4967 | 0.002732 | 0.007418 | up-regulated |
| PHACTR3 | 116154 | 2.0337 | 1.80545 | 0.002767 | 0.007501 | up-regulated |
| CALHM3 | 119395 | 4.35722 | -1.7505 | 0.002768 | 0.007505 | up-regulated |
| EVX1 | 2128 | 2.78948 | 1.00373 | 0.00277 | 0.007508 | up-regulated |
| WFDC13 | 164237 | 2.40777 | -3.2141 | 0.002779 | 0.007528 | up-regulated |
| FGG | 2266 | 13.864 | 7.57025 | 0.002804 | 0.007585 | up-regulated |
| CPN2 | 1370 | 6.32683 | 2.222 | 0.002851 | 0.007694 | up-regulated |
| MAGEA4 | 4103 | 8.0121 | 0.46809 | 0.002853 | 0.007698 | up-regulated |
| DNAH12 | 201625 | 2.43473 | -2.3058 | 0.002932 | 0.007881 | up-regulated |
| VWCE | 220001 | 2.45092 | 0.51396 | 0.002939 | 0.007895 | up-regulated |
| HIST1H1B | 3009 | 2.36609 | -1.1854 | 0.002952 | 0.007922 | up-regulated |
| PRSS1 | 5644 | 5.07778 | 0.83961 | 0.003055 | 0.008162 | up-regulated |
| ITIH3 | 3699 | 5.37222 | 4.51756 | 0.003112 | 0.008305 | up-regulated |
| KRT37 | 8688 | 4.47679 | -3.3231 | 0.003129 | 0.008341 | up-regulated |
| SRD5A2 | 6716 | 6.29881 | -0.7794 | 0.003129 | 0.008341 | up-regulated |
| GAST | 2520 | 4.65084 | -3.1998 | 0.003153 | 0.008392 | up-regulated |
| IFITM5 | 387733 | 3.49505 | -3.4058 | 0.003168 | 0.008425 | up-regulated |
| ASGR2 | 433 | 4.61951 | 2.64139 | 0.003184 | 0.008456 | up-regulated |
| MAGEA2 | 4101 | 9.77889 | 2.94114 | 0.003196 | 0.008484 | up-regulated |
| APOA2 | 336 | 10.8784 | 5.405 | 0.003237 | 0.008577 | up-regulated |
| NKX2-2 | 4821 | -2.083 | -1.6068 | 0.003263 | 0.008638 | down-regulated |
| FOXL2 | 668 | 5.10019 | -2.947 | 0.003318 | 0.00876 | up-regulated |
| SLC28A2 | 9153 | -2.2188 | 2.70507 | 0.003348 | 0.008827 | down-regulated |
| TNNC2 | 7125 | 2.18826 | 3.79798 | 0.003379 | 0.008898 | up-regulated |
| PYY2 | 23615 | 2.0604 | -2.9599 | 0.003403 | 0.008957 | up-regulated |
| SCARNA2 | 677766 | 2.10353 | -0.8423 | 0.003446 | 0.009059 | up-regulated |
| CYP4A11 | 1579 | 9.16771 | 2.82697 | 0.003494 | 0.009164 | up-regulated |
| CYP2C8 | 1558 | 5.15139 | 2.79906 | 0.00351 | 0.009204 | up-regulated |
| MAGEA11 | 4110 | 6.55081 | 1.2666 | 0.003523 | 0.009232 | up-regulated |
| FAM66D | 100132923 | 2.57356 | -0.0807 | 0.00356 | 0.009316 | up-regulated |
| UROC1 | 131669 | 5.58375 | -1.4052 | 0.003601 | 0.009404 | up-regulated |
| KIF25 | 3834 | 2.57056 | -1.0115 | 0.003601 | 0.009404 | up-regulated |
| PRSS50 | 29122 | 3.90896 | -2.3041 | 0.003624 | 0.00945 | up-regulated |
| SYT8 | 90019 | 2.29029 | 0.72853 | 0.003699 | 0.009626 | up-regulated |
| KRT38 | 8687 | 4.66084 | -3.2133 | 0.0037 | 0.009627 | up-regulated |
| GLTPD2 | 388323 | 3.91298 | -1.6948 | 0.003702 | 0.009628 | up-regulated |
| ATP6V1B1 | 525 | 2.15118 | -1.2477 | 0.003718 | 0.009663 | up-regulated |
| SYT9 | 143425 | -2.0141 | -2.862 | 0.003722 | 0.009669 | down-regulated |
| DMRTA2 | 63950 | 3.81041 | -0.0129 | 0.003782 | 0.009809 | up-regulated |
| TEX19 | 400629 | 2.11443 | -2.5523 | 0.003798 | 0.009846 | up-regulated |
| SERPINB4 | 6318 | 6.69007 | -1.6959 | 0.003827 | 0.009907 | up-regulated |
| GOLGA6L1 | 283767 | 4.34652 | -2.4729 | 0.003848 | 0.009953 | up-regulated |
| EFCAB3 | 146779 | 2.65883 | -3.7993 | 0.003875 | 0.010013 | up-regulated |
| CLVS1 | 157807 | 2.66825 | -0.2665 | 0.003898 | 0.010061 | up-regulated |
| TNFSF18 | 8995 | 2.41483 | -2.4396 | 0.003919 | 0.010105 | up-regulated |
| CRP | 1401 | 14.568 | 7.50703 | 0.003936 | 0.010146 | up-regulated |
| CARTPT | 9607 | -2.0972 | 0.44631 | 0.003939 | 0.010151 | down-regulated |
| EPHA8 | 2046 | 4.20328 | -2.0845 | 0.003987 | 0.010262 | up-regulated |
| GOLGA8G | 283768 | 4.19817 | -2.9212 | 0.004009 | 0.010309 | up-regulated |
| ITLN2 | 142683 | 6.52383 | 3.17666 | 0.004036 | 0.010368 | up-regulated |
| POU6F2 | 11281 | 4.70645 | -0.1078 | 0.004058 | 0.010419 | up-regulated |
| TREML3 | 340206 | 2.70101 | -3.3129 | 0.004173 | 0.01067 | up-regulated |
| MAGEA9B | 728269 | 7.65369 | 0.55518 | 0.004177 | 0.010677 | up-regulated |
| MUC21 | 394263 | 4.30919 | -3.3957 | 0.004226 | 0.010784 | up-regulated |
| CLLU1 | 574028 | 2.53128 | -2.1628 | 0.004248 | 0.010833 | up-regulated |
| FGF23 | 8074 | 4.63581 | -2.7078 | 0.004299 | 0.010949 | up-regulated |
| CPB2 | 1361 | 9.08993 | 2.28671 | 0.004372 | 0.011112 | up-regulated |
| A2ML1 | 144568 | 7.6795 | 0.0595 | 0.004393 | 0.011148 | up-regulated |
| STOML3 | 161003 | 2.4606 | -0.8309 | 0.004419 | 0.011205 | up-regulated |
| LRP2 | 4036 | 5.61362 | 0.2559 | 0.004428 | 0.011219 | up-regulated |
| IL5 | 3567 | 3.49542 | -3.7993 | 0.004448 | 0.011263 | up-regulated |
| GJB6 | 10804 | 2.85619 | -1.8136 | 0.0045 | 0.011379 | up-regulated |
| MAGEC2 | 51438 | 9.9418 | 1.36304 | 0.004586 | 0.011577 | up-regulated |
| WFDC10A | 140832 | 2.85887 | -3.0204 | 0.004591 | 0.011589 | up-regulated |
| SPZ1 | 84654 | 3.83543 | -3.6581 | 0.004633 | 0.011679 | up-regulated |
| BIRC7 | 79444 | 2.271 | -0.1217 | 0.004652 | 0.01172 | up-regulated |
| KRT40 | 125115 | 4.95223 | 4.36941 | 0.004673 | 0.011769 | up-regulated |
| GPR109A | 338442 | 2.49097 | 1.80069 | 0.004701 | 0.011829 | up-regulated |
| CSAG1 | 158511 | 7.34455 | 1.39238 | 0.004747 | 0.011929 | up-regulated |
| KRT34 | 3885 | 4.86389 | -3.0582 | 0.004784 | 0.012011 | up-regulated |
| MMP20 | 9313 | 3.19102 | -3.2854 | 0.00484 | 0.012138 | up-regulated |
| OCA2 | 4948 | 2.81916 | 0.70971 | 0.00487 | 0.012203 | up-regulated |
| NLRP4 | 147945 | -2.0868 | -3.3637 | 0.004888 | 0.012239 | down-regulated |
| DMP1 | 1758 | 3.49976 | -3.7931 | 0.005054 | 0.012585 | up-regulated |
| SBSN | 374897 | 2.18948 | -2.3101 | 0.005059 | 0.012595 | up-regulated |
| RHCG | 51458 | 2.15961 | 1.55896 | 0.005132 | 0.012761 | up-regulated |
| C6orf126 | 389383 | 3.52493 | -3.7883 | 0.005151 | 0.012804 | up-regulated |
| ADH4 | 127 | 4.58569 | 3.62497 | 0.005152 | 0.012804 | up-regulated |
| CALB1 | 793 | 5.24461 | 3.13829 | 0.005153 | 0.012804 | up-regulated |
| ABCG5 | 64240 | 2.72073 | -0.464 | 0.005164 | 0.012826 | up-regulated |
| LCT | 3938 | 2.70447 | -2.355 | 0.005251 | 0.01302 | up-regulated |
| TPH1 | 7166 | -2.0015 | 0.45524 | 0.005277 | 0.013073 | down-regulated |
| REN | 5972 | 3.01744 | 1.72349 | 0.005353 | 0.013235 | up-regulated |
| HOXC6 | 3223 | 3.36183 | -0.0006 | 0.005451 | 0.013445 | up-regulated |
| MORN3 | 283385 | 2.724 | -0.8586 | 0.005452 | 0.013445 | up-regulated |
| ISM1 | 140862 | 2.24989 | 2.39662 | 0.005455 | 0.013451 | up-regulated |
| CALML6 | 163688 | 2.40518 | -3.275 | 0.00547 | 0.013481 | up-regulated |
| SOHLH2 | 54937 | -2.1346 | -1.2396 | 0.005484 | 0.013502 | down-regulated |
| APOA5 | 116519 | 8.98855 | 1.1484 | 0.00556 | 0.013656 | up-regulated |
| PLA2G3 | 50487 | 4.04411 | 0.22713 | 0.005604 | 0.013748 | up-regulated |
| RPL29P2 | 118432 | 2.22244 | -3.7227 | 0.005629 | 0.013804 | up-regulated |
| FBXW10 | 10517 | 2.28606 | -3.2903 | 0.005726 | 0.014017 | up-regulated |
| S100A7 | 6278 | 4.1668 | -2.3017 | 0.005934 | 0.014441 | up-regulated |
| ALOX12P2 | 245 | 3.81835 | -2.5177 | 0.005998 | 0.014571 | up-regulated |
| PRL | 5617 | 3.42335 | -3.8228 | 0.006015 | 0.014609 | up-regulated |
| RBP4 | 5950 | 2.55772 | 5.74211 | 0.006016 | 0.014611 | up-regulated |
| LEP | 3952 | -2.0437 | -0.795 | 0.006104 | 0.01479 | down-regulated |
| TSIX | 9383 | 6.23187 | 4.58408 | 0.006121 | 0.014821 | up-regulated |
| KISS1R | 84634 | 2.32181 | -2.6948 | 0.006123 | 0.014824 | up-regulated |
| FAM183A | 440585 | 3.29242 | -2.5908 | 0.006134 | 0.01484 | up-regulated |
| LY6D | 8581 | 3.74827 | -0.6184 | 0.006228 | 0.01505 | up-regulated |
| ALOXE3 | 59344 | 2.0675 | -2.4605 | 0.006251 | 0.015088 | up-regulated |
| TCP11 | 6954 | 3.95359 | -1.2652 | 0.006307 | 0.0152 | up-regulated |
| CLDN6 | 9074 | 3.76078 | -1.8027 | 0.006325 | 0.015231 | up-regulated |
| FAM71E2 | 284418 | 3.30195 | -3.2367 | 0.006451 | 0.015484 | up-regulated |
| TUBA3E | 112714 | 4.86914 | -0.7318 | 0.006472 | 0.015518 | up-regulated |
| IGFN1 | 91156 | 2.21764 | -0.6922 | 0.006865 | 0.01633 | up-regulated |
| AFP | 174 | 3.69722 | -3.3102 | 0.00688 | 0.016363 | up-regulated |
| KCTD16 | 57528 | 2.26121 | -0.3401 | 0.006922 | 0.016447 | up-regulated |
| LOC400794 | 400794 | 3.16457 | -2.1974 | 0.006964 | 0.016542 | up-regulated |
| PNMT | 5409 | 2.58906 | -0.7592 | 0.007035 | 0.016687 | up-regulated |
| C5orf27 | 202299 | 3.51719 | -1.1605 | 0.007044 | 0.016703 | up-regulated |
| LOC285733 | 285733 | 3.85811 | -1.7853 | 0.007049 | 0.01671 | up-regulated |
| INHBC | 3626 | 5.07336 | 0.14961 | 0.007055 | 0.016718 | up-regulated |
| LGALS7 | 3963 | 2.71516 | -1.9039 | 0.007089 | 0.016793 | up-regulated |
| TLX3 | 30012 | 3.65582 | -3.7371 | 0.007112 | 0.016842 | up-regulated |
| AQP5 | 362 | 2.28308 | -1.296 | 0.007185 | 0.016992 | up-regulated |
| C5orf38 | 153571 | 3.12117 | 0.03902 | 0.007204 | 0.017027 | up-regulated |
| ANGPTL3 | 27329 | 7.41534 | 1.64596 | 0.007215 | 0.01705 | up-regulated |
| GRIN3B | 116444 | 2.35409 | -3.0326 | 0.007259 | 0.017137 | up-regulated |
| KRT83 | 3889 | 2.79289 | -1.6952 | 0.007271 | 0.01716 | up-regulated |
| WIF1 | 11197 | 3.58672 | 2.83071 | 0.007362 | 0.017335 | up-regulated |
| HGFAC | 3083 | 7.7562 | 0.45804 | 0.007454 | 0.017532 | up-regulated |
| PON1 | 5444 | 6.34237 | 1.57361 | 0.00754 | 0.017708 | up-regulated |
| CYP2C19 | 1557 | 3.75829 | -1.0805 | 0.007582 | 0.017785 | up-regulated |
| FGF17 | 8822 | 2.08135 | -2.698 | 0.007584 | 0.017788 | up-regulated |
| IRX2 | 153572 | 2.93471 | 2.46302 | 0.007586 | 0.01779 | up-regulated |
| ITLN1 | 55600 | -2.096 | 7.08169 | 0.007622 | 0.017871 | down-regulated |
| IGFL2 | 147920 | 2.5816 | 0.5443 | 0.007776 | 0.018188 | up-regulated |
| CYP8B1 | 1582 | 5.8507 | 2.76157 | 0.007779 | 0.018191 | up-regulated |
| KRTAP1-1 | 81851 | 3.96201 | -3.5937 | 0.00781 | 0.018247 | up-regulated |
| FGL1 | 2267 | 11.129 | 5.08596 | 0.008012 | 0.018669 | up-regulated |
| KRTAP5-10 | 387273 | 2.54462 | -3.8157 | 0.008045 | 0.018733 | up-regulated |
| ITIH1 | 3697 | 8.65064 | 4.13759 | 0.008183 | 0.019001 | up-regulated |
| SHBG | 6462 | 3.80885 | -0.0812 | 0.008242 | 0.019107 | up-regulated |
| DKK1 | 22943 | 2.77631 | 1.4107 | 0.008265 | 0.019157 | up-regulated |
| SLCO1A2 | 6579 | 3.63682 | -1.5378 | 0.008283 | 0.019191 | up-regulated |
| GRPR | 2925 | 2.13632 | 0.29256 | 0.008289 | 0.0192 | up-regulated |
| C9 | 735 | 8.52107 | 3.79245 | 0.008319 | 0.019262 | up-regulated |
| CGB7 | 94027 | 2.80429 | -3.4696 | 0.008331 | 0.019283 | up-regulated |
| CST6 | 1474 | 2.50756 | -0.7962 | 0.008393 | 0.019402 | up-regulated |
| UGT2B4 | 7363 | 7.81551 | 2.47097 | 0.00846 | 0.019531 | up-regulated |
| ASIP | 434 | -2.3668 | -1.7615 | 0.00856 | 0.019735 | down-regulated |
| NPTX2 | 4885 | 2.50937 | 4.1387 | 0.008709 | 0.020038 | up-regulated |
| KRT74 | 121391 | 5.12122 | -2.9304 | 0.008854 | 0.020326 | up-regulated |
| CLDN18 | 51208 | 4.88013 | 2.56617 | 0.008897 | 0.020416 | up-regulated |
| MIOX | 55586 | 2.6138 | -3.0928 | 0.008907 | 0.02043 | up-regulated |
| CRYAA | 1409 | 5.45986 | -2.0887 | 0.009216 | 0.021016 | up-regulated |
| SLC6A3 | 6531 | 2.56022 | -2.7418 | 0.009289 | 0.021162 | up-regulated |
| PAGE2B | 389860 | 5.62171 | -2.5309 | 0.009291 | 0.021163 | up-regulated |
| METTL11B | 149281 | 3.32892 | -3.8612 | 0.009293 | 0.021165 | up-regulated |
| FGF21 | 26291 | 6.28583 | -2.0653 | 0.00943 | 0.021442 | up-regulated |
| SERPINB7 | 8710 | 2.84558 | -0.5293 | 0.009453 | 0.021487 | up-regulated |
| APOH | 350 | 5.60864 | 5.62868 | 0.009489 | 0.021554 | up-regulated |
| NMUR2 | 56923 | 4.43784 | -0.523 | 0.009557 | 0.021689 | up-regulated |
| GRM1 | 2911 | 4.7292 | 1.15358 | 0.009656 | 0.021882 | up-regulated |
| UNC5A | 90249 | 2.80079 | 0.4542 | 0.009721 | 0.02201 | up-regulated |
| APOF | 319 | 8.4579 | 1.10122 | 0.009777 | 0.022122 | up-regulated |
| FLJ16779 | 100192386 | 2.17654 | 0.04981 | 0.009821 | 0.022202 | up-regulated |
| LOC731789 | 731789 | 6.42429 | -0.5187 | 0.010066 | 0.022675 | up-regulated |
| PRB3 | 5544 | 2.04692 | -3.2109 | 0.010134 | 0.022796 | up-regulated |
| FSTL4 | 23105 | 2.22653 | 0.52471 | 0.010283 | 0.023108 | up-regulated |
| DEFB4A | 1673 | 4.52459 | -3.2851 | 0.010442 | 0.023434 | up-regulated |
| FAM83C | 128876 | 2.64717 | -0.5277 | 0.010501 | 0.023555 | up-regulated |
| TRIM72 | 493829 | 4.93657 | -0.5704 | 0.010541 | 0.023634 | up-regulated |
| HOXC13 | 3229 | 6.09638 | -1.4966 | 0.010699 | 0.023951 | up-regulated |
| GATA4 | 2626 | 4.6971 | 0.76131 | 0.010716 | 0.023985 | up-regulated |
| HOXC8 | 3224 | 3.44939 | -1.9796 | 0.010733 | 0.024017 | up-regulated |
| SOHLH1 | 402381 | 4.17193 | -3.4818 | 0.010766 | 0.024077 | up-regulated |
| APOC3 | 345 | 11.084 | 4.46885 | 0.010813 | 0.024172 | up-regulated |
| KLK5 | 25818 | 5.76815 | -1.2779 | 0.011131 | 0.024778 | up-regulated |
| GCM1 | 8521 | 2.26298 | -2.2313 | 0.011231 | 0.024965 | up-regulated |
| C2orf66 | 401027 | 2.23352 | -2.6796 | 0.011249 | 0.02499 | up-regulated |
| HAL | 3034 | 2.5304 | -0.0134 | 0.011251 | 0.024993 | up-regulated |
| HTR2C | 3358 | 7.04227 | -1.3817 | 0.011454 | 0.025384 | up-regulated |
| IRX3 | 79191 | 2.03297 | -0.3012 | 0.011465 | 0.025405 | up-regulated |
| C22orf45 | 646023 | 2.18816 | -1.2764 | 0.011542 | 0.025552 | up-regulated |
| NKX2-1 | 7080 | 6.58552 | 0.07653 | 0.011792 | 0.026034 | up-regulated |
| RGR | 5995 | 3.67575 | -2.3238 | 0.012133 | 0.026679 | up-regulated |
| APOC4 | 346 | 6.86632 | 0.4067 | 0.012161 | 0.026735 | up-regulated |
| RSPO4 | 343637 | 3.04873 | -0.209 | 0.012309 | 0.027 | up-regulated |
| A1BG | 1 | 3.33079 | 4.40338 | 0.01234 | 0.027058 | up-regulated |
| ACTBL2 | 345651 | 3.25938 | -3.5409 | 0.012371 | 0.027118 | up-regulated |
| DNAJB13 | 374407 | 2.1766 | -3.6228 | 0.012509 | 0.027366 | up-regulated |
| TUSC5 | 286753 | -2.18 | -0.4197 | 0.012544 | 0.027421 | down-regulated |
| KRTAP3-2 | 83897 | 3.41397 | -2.89 | 0.012591 | 0.027493 | up-regulated |
| C17orf77 | 146723 | 2.54345 | 0.94495 | 0.012992 | 0.028256 | up-regulated |
| KLK12 | 43849 | 2.40911 | 1.66607 | 0.013138 | 0.028554 | up-regulated |
| NTF4 | 4909 | 3.36955 | -3.4818 | 0.013223 | 0.0287 | up-regulated |
| PAPL | 390928 | 3.23329 | -3.1092 | 0.013343 | 0.028929 | up-regulated |
| IZUMO1 | 284359 | 2.36606 | -3.6677 | 0.013487 | 0.029188 | up-regulated |
| TBC1D3G | 654341 | 4.66094 | -0.7793 | 0.013528 | 0.02926 | up-regulated |
| MYO18B | 84700 | 2.59114 | -1.4484 | 0.01365 | 0.029505 | up-regulated |
| GNMT | 27232 | 2.35482 | -1.4765 | 0.013738 | 0.029679 | up-regulated |
| BARX1 | 56033 | 2.73934 | -0.881 | 0.013877 | 0.029948 | up-regulated |
| SLED1 | 643036 | 2.05181 | -3.4765 | 0.014084 | 0.030322 | up-regulated |
| LOC284798 | 284798 | 3.01196 | -2.729 | 0.014147 | 0.030432 | up-regulated |
| RIPPLY1 | 92129 | 2.00721 | -2.6336 | 0.014185 | 0.030502 | up-regulated |
| HS3ST4 | 9951 | 5.02084 | 1.02548 | 0.014363 | 0.03083 | up-regulated |
| SLC2A2 | 6514 | 8.09811 | 2.46321 | 0.014429 | 0.030949 | up-regulated |
| HSD17B13 | 345275 | 2.95892 | 1.63111 | 0.014446 | 0.03098 | up-regulated |
| HSP90AB4P | 664618 | 2.02765 | -3.4153 | 0.014724 | 0.031528 | up-regulated |
| FAM155B | 27112 | 2.18841 | 2.11385 | 0.01473 | 0.031533 | up-regulated |
| GC | 2638 | 13.1274 | 5.26423 | 0.014767 | 0.031592 | up-regulated |
| CACNA1E | 777 | 2.74904 | 1.30651 | 0.014915 | 0.031868 | up-regulated |
| MAGEC1 | 9947 | 7.75673 | 0.01808 | 0.01495 | 0.031923 | up-regulated |
| SERPINB2 | 5055 | 3.26254 | -0.5242 | 0.014957 | 0.031935 | up-regulated |
| C8B | 732 | 11.324 | 2.72813 | 0.015109 | 0.032217 | up-regulated |
| CYP3A7 | 1551 | 2.18994 | 0.57465 | 0.015402 | 0.032722 | up-regulated |
| LCE1E | 353135 | 5.26614 | -2.8202 | 0.015524 | 0.032948 | up-regulated |
| TF | 7018 | 4.69559 | 5.5107 | 0.015537 | 0.032971 | up-regulated |
| SLC22A7 | 10864 | 8.51943 | 1.38998 | 0.015541 | 0.032971 | up-regulated |
| OXT | 5020 | 2.84284 | -3.7525 | 0.015624 | 0.033121 | up-regulated |
| XAGE2 | 9502 | 6.15851 | -2.1394 | 0.01572 | 0.033284 | up-regulated |
| C19orf34 | 255193 | 2.15993 | -3.7499 | 0.015741 | 0.033318 | up-regulated |
| LHB | 3972 | 2.3687 | -3.7098 | 0.016154 | 0.03409 | up-regulated |
| MAGEA10 | 4109 | 7.45329 | -1.0442 | 0.016447 | 0.034629 | up-regulated |
| UGT2A1 | 10941 | 4.55339 | -3.2772 | 0.016492 | 0.034721 | up-regulated |
| WNT7A | 7476 | 3.15328 | -2.12 | 0.016577 | 0.034875 | up-regulated |
| UPB1 | 51733 | 3.1464 | 0.49973 | 0.016718 | 0.035123 | up-regulated |
| HSD17B3 | 3293 | 2.52067 | -1.1515 | 0.016992 | 0.035621 | up-regulated |
| LOC116437 | 116437 | 2.7437 | -3.7726 | 0.017014 | 0.035653 | up-regulated |
| CSAG3 | 389903 | 5.76038 | 0.7421 | 0.017072 | 0.035766 | up-regulated |
| ? | 729884 | 4.62955 | -0.169 | 0.0173 | 0.036165 | up-regulated |
| SERPINA11 | 256394 | 6.41687 | 2.16152 | 0.017434 | 0.036396 | up-regulated |
| PRB2 | 653247 | 4.10086 | -2.9753 | 0.017434 | 0.036396 | up-regulated |
| KRT35 | 3886 | 3.74314 | -3.6903 | 0.01749 | 0.036503 | up-regulated |
| ? | 553137 | 4.89033 | -3.0979 | 0.017706 | 0.036873 | up-regulated |
| FAM75A2 | 642265 | 3.54685 | -3.7647 | 0.017976 | 0.037367 | up-regulated |
| PLG | 5340 | 10.9525 | 4.08197 | 0.018028 | 0.037454 | up-regulated |
| CYP2A6 | 1548 | 7.79496 | 3.03961 | 0.018045 | 0.037481 | up-regulated |
| SUN3 | 256979 | 2.41394 | -3.632 | 0.018094 | 0.037569 | up-regulated |
| CFHR1 | 3078 | 8.14951 | 2.93444 | 0.018118 | 0.037615 | up-regulated |
| C8A | 731 | 10.923 | 2.32964 | 0.018133 | 0.037641 | up-regulated |
| FLJ45983 | 399717 | 2.0298 | -3.8246 | 0.018174 | 0.037705 | up-regulated |
| HPD | 3242 | 5.18544 | 2.74197 | 0.01824 | 0.037824 | up-regulated |
| FGF8 | 2253 | 2.74436 | -3.5734 | 0.018259 | 0.037856 | up-regulated |
| HIST1H2AB | 8335 | 2.24822 | -3.5863 | 0.018437 | 0.03818 | up-regulated |
| LCN15 | 389812 | 3.9607 | 6.11158 | 0.018451 | 0.038205 | up-regulated |
| ASB4 | 51666 | 3.03727 | -1.181 | 0.018623 | 0.0385 | up-regulated |
| ANKRD1 | 27063 | 2.52576 | -2.3898 | 0.018626 | 0.0385 | up-regulated |
| ARG1 | 383 | 5.27067 | 1.65888 | 0.018673 | 0.038571 | up-regulated |
| ASPDH | 554235 | 4.59137 | -1.2409 | 0.018707 | 0.038628 | up-regulated |
| IL1F7 | 27178 | 3.05072 | -0.5719 | 0.018829 | 0.038832 | up-regulated |
| PCSK1 | 5122 | 2.49384 | 5.25733 | 0.019095 | 0.039314 | up-regulated |
| HCN1 | 348980 | 2.62979 | 0.95017 | 0.019119 | 0.039357 | up-regulated |
| MAGEA1 | 4100 | 7.50466 | -0.9543 | 0.019126 | 0.039367 | up-regulated |
| DMRT3 | 58524 | 2.75379 | -1.865 | 0.019204 | 0.039503 | up-regulated |
| TMEM213 | 155006 | 2.77082 | -3.3062 | 0.019386 | 0.03981 | up-regulated |
| APCS | 325 | 11.7875 | 3.18948 | 0.019398 | 0.039819 | up-regulated |
| HPR | 3250 | 6.08231 | 2.56966 | 0.019502 | 0.039996 | up-regulated |
| CXorf22 | 170063 | 3.61122 | -3.7327 | 0.019621 | 0.0402 | up-regulated |
| MUC16 | 94025 | 3.40714 | -0.7498 | 0.019758 | 0.040426 | up-regulated |
| C3orf45 | 132228 | 3.90095 | 0.9932 | 0.019788 | 0.040483 | up-regulated |
| DPYS | 1807 | 6.19591 | 1.03093 | 0.019876 | 0.040639 | up-regulated |
| HAO2 | 51179 | 5.30604 | -0.0504 | 0.020326 | 0.04149 | up-regulated |
| FTCD | 10841 | 5.60634 | 1.82975 | 0.020638 | 0.041983 | up-regulated |
| CLDN10 | 9071 | 3.77796 | 1.0921 | 0.020829 | 0.042339 | up-regulated |
| UGT1A4 | 54657 | 9.31564 | 2.20704 | 0.020862 | 0.042397 | up-regulated |
| LPA | 4018 | 4.39773 | -0.1754 | 0.02106 | 0.042728 | up-regulated |
| HAO1 | 54363 | 9.71939 | 1.13956 | 0.021121 | 0.042821 | up-regulated |
| ENPP7 | 339221 | 3.83935 | -2.5995 | 0.021183 | 0.042929 | up-regulated |
| ZNF556 | 80032 | 3.53617 | -2.317 | 0.02128 | 0.043109 | up-regulated |
| AMDHD1 | 144193 | 2.25222 | 0.44887 | 0.021502 | 0.043472 | up-regulated |
| TBX5 | 6910 | 3.20765 | -3.0271 | 0.021699 | 0.043784 | up-regulated |
| DNMT3L | 29947 | 4.13292 | -3.4977 | 0.021739 | 0.043842 | up-regulated |
| SP9 | 100131390 | 3.87575 | -3.0988 | 0.021752 | 0.043861 | up-regulated |
| CFHR3 | 10878 | 3.67726 | 0.52463 | 0.021817 | 0.043958 | up-regulated |
| SERPINA1 | 5265 | 2.18664 | 9.80917 | 0.021958 | 0.044212 | up-regulated |
| OBP2A | 29991 | 3.06717 | -3.5909 | 0.022014 | 0.044314 | up-regulated |
| APOC1P1 | 342 | 6.9875 | -1.4594 | 0.02203 | 0.044327 | up-regulated |
| SOX21 | 11166 | 4.85239 | -2.0885 | 0.022297 | 0.044769 | up-regulated |
| SLC14A1 | 6563 | 2.51291 | 4.08084 | 0.022388 | 0.044923 | up-regulated |
| LECT2 | 3950 | 8.27445 | -0.2644 | 0.022598 | 0.045293 | up-regulated |
| GREB1L | 80000 | 3.01492 | -0.1711 | 0.023097 | 0.046141 | up-regulated |
| PAX2 | 5076 | 2.6492 | -2.9134 | 0.023244 | 0.046387 | up-regulated |
| CFHR5 | 81494 | 9.61399 | 1.03594 | 0.023323 | 0.04651 | up-regulated |
| VNN3 | 55350 | 2.38924 | -0.5764 | 0.023657 | 0.047098 | up-regulated |
| PKHD1 | 5314 | 2.46332 | 0.515 | 0.023838 | 0.047391 | up-regulated |
| FAM9A | 171482 | 4.08593 | -3.5253 | 0.02402 | 0.047687 | up-regulated |
| SPINLW1 | 57119 | 3.07288 | -2.962 | 0.024022 | 0.047687 | up-regulated |
| KCNT1 | 57582 | 2.34785 | -0.418 | 0.024365 | 0.048273 | up-regulated |
| FXYD2 | 486 | 2.08668 | -0.3855 | 0.024707 | 0.048872 | up-regulated |
| MUC15 | 143662 | 4.82822 | -0.2901 | 0.024917 | 0.049244 | up-regulated |
| SERPINA3 | 12 | 3.12732 | 7.01326 | 0.024929 | 0.049262 | up-regulated |
| F9 | 2158 | 10.5836 | 1.99294 | 0.024967 | 0.049327 | up-regulated |
| PGLYRP4 | 57115 | 2.77916 | -2.6293 | 0.024975 | 0.049337 | up-regulated |
| SNORA76 | 677842 | 2.34566 | -3.6873 | 0.025083 | 0.049517 | up-regulated |
| SLC10A1 | 6554 | 4.32392 | 0.55908 | 0.025363 | 0.049977 | up-regulated |

Table contains Log2 Fold Change (logFC), P-Value and adjusted P-value (FDR) for each gene.

**Supplementary Table 3: Official gene names and Entrez gene IDs of sixteen modules listed in the gene-module**

| No. | Module name | Gene number | Official gene name / Entrez gene ID |
| --- | --- | --- | --- |
| 1 | Black module | 121 | MT1M\|4499, FUT9\|10690, MT1F\|4494, BEST2\|54831, UGT2B15\|7366, LAMA1\|284217, SHISA3\|152573, LRMP\|4033, FAM5C\|339479, REP15\|387849, CPB1\|1360, TPSG1\|25823, SLC4A10\|57282, ADH1C\|126, VIT\|5212, LOC389033\|389033, SPINK2\|6691, VSIG2\|23584, PLAC2\|257000, PLCXD3\|345557, KLF4\|9314, UGT1A10\|54575, FABP2\|2169, ROPN1\|54763, B4GALNT2\|124872, PPY\|5539, FAM46C\|54855, LOC643763\|643763, FRMD3\|257019, HSPA2\|3306, METTL7A\|25840, CLCA1\|1179, SCGB2A1\|4246, RNF125\|54941, KCNMB2\|10242, B3GNT6\|192134, NRAP\|4892, CNTN3\|5067, RETNLB\|84666, BMX\|660, C9orf135\|138255, ABCC8\|6833, ITLN1\|55600, HS3ST6\|64711, RIMS3\|9783, FAM181B\|220382, NPY6R\|4888, SLC9A2\|6549, GPR120\|338557, PHGR1\|644844, ZBTB7C\|201501, MSTN\|2660, SH3GL2\|6456, FETUB\|26998, FAM55D\|54827, RHBDL2\|54933, MB\|4151, SPDEF\|25803, TMEM61\|199964, SPDYC\|387778, CA12\|771, LRRC26\|389816, DRP2\|1821, KLK1\|3816, WFIKKN2\|124857, NPY1R\|4886, BCAS1\|8537, LOC728606\|728606, KCNA6\|3742, KCNE2\|9992, FAM177B\|400823, ANO7\|50636, CALN1\|83698, CAPN9\|10753, ST3GAL4\|6484, CTSE\|1510, PGA3\|643834, KLK8\|11202, LOC283392\|283392, ATOH1\|474, C20orf151\|140893, MTUS2\|23281, IGSF5\|150084, SLITRK6\|84189, RPL10L\|140801, PACSIN1\|29993, NOL4\|8715, KIF19\|124602, LOC554202\|554202, RAB27B\|5874, SLC5A8\|160728, SOX14\|8403, NR1H4\|9971, FCGBP\|8857, WFDC2\|10406, WFDC10B\|280664, GP9\|2815, APOBEC1\|339, HOXD12\|3238, GALNT8\|26290, PRSS41\|360226, NPY5R\|4889, TCL6\|27004, C11orf90\|387804, ALDH1L1\|10840, ANO3\|63982, LOC100144604\|100144604, ORM2\|5005, KLK3\|354, TPRXL\|348825, MUC4\|4585, C1orf125\|126859, MUC2\|4583, VTCN1\|79679, WISP3\|8838, CXCL17\|284340, GIF\|2694, BHMT\|635, TRY6\|154754, PRSS1\|5644, CCDC60\|160777 |
| 2 | Blue module | 283 | LYVE1\|10894, FIGF\|2277, SCGN\|10590, C16orf89\|146556, SFRP1\|6422, GPM6B\|2824, OGN\|4969, DPT\|1805, GSTM5\|2949, GLIPR2\|152007, ADCYAP1R1\|117, PCOLCE2\|26577, FAM180B\|399888, LPAR1\|1902, DCLK1\|9201, ATP6V1G2\|534, CMA1\|1215, ASPA\|443, C7\|730, GRIK1\|2897, SGCG\|6445, PDE6A\|5145, PRKAR2B\|5577, SLC9A9\|285195, SALL4\|57167, SOX4\|6659, ABCA9\|10350, CILP\|8483, GGTA1\|2681, KIF5C\|3800, CXCL12\|6387, ANGPTL5\|253935, C14orf139\|79686, FAM70A\|55026, SSTR2\|6752, GPR112\|139378, EML1\|2009, ZBTB16\|7704, CNTN4\|152330, ABCA6\|23460, ADAMTSL1\|92949, LRCH2\|57631, LRRN2\|10446, ZMAT4\|79698, TMTC1\|83857, ZNF781\|163115, ARMCX1\|51309, PER3\|8863, TPX2\|22974, ADH1A\|124, EDIL3\|10085, NR3C1\|2908, CLIP4\|79745, GYPC\|2995, GABRD\|2563, MFAP5\|8076, SGCE\|8910, NAALADL1\|10004, SEMA3D\|223117, HIF3A\|64344, OSBPL3\|26031, FAM13C\|220965, AQPEP\|206338, SLIT3\|6586, PRICKLE2\|166336, LOC339524\|339524, C20orf194\|25943, MGAT4C\|25834, SFRP5\|6425, ARHGAP20\|57569, CD300LG\|146894, SSPN\|8082, PDE3A\|5139, NTNG1\|22854, RTN1\|6252, CALY\|50632, TLL1\|7092, CLDN1\|9076, LOC144571\|144571, LOC284276\|284276, PPP1R3C\|5507, PALM\|5064, MAGEE2\|139599, EFHA2\|286097, WNT2\|7472, C2orf74\|339804, RNF180\|285671, MAS1L\|116511, NAALAD2\|10003, PDE1A\|5136, CP\|1356, ASAM\|79827, FABP6\|2172, FAM123A\|219287, DPYD\|1806, GLI3\|2737, ADRB2\|154, OMD\|4958, ZNF626\|199777, SLC26A2\|1836, CACNA2D1\|781, LRP1B\|53353, BNC2\|54796, STAC\|6769, KLK15\|55554, C7orf68\|29923, NPAS3\|64067, SPATA12\|353324, GPX3\|2878, CD1C\|911, UBE2C\|11065, F13A1\|2162, LRRK2\|120892, C2\|717, DDR2\|4921, GSTA1\|2938, MGC42105\|167359, SFTA1P\|207107, NXPH2\|11249, ATG9B\|285973, DLX4\|1748, GSDMC\|56169, ZDHHC15\|158866, TUB\|7275, CHRM4\|1132, STON1\|11037, RPH3A\|22895, C13orf38\|728591, HSD3B2\|3284, DIRC3\|729582, AMELX\|265, TMEM130\|222865, TNNT3\|7140, SLIT2\|9353, RBMS3\|27303, EYA2\|2139, KCNC1\|3746, ENHO\|375704, GALNTL2\|117248, LUZP2\|338645, HEPN1\|641654, RGS22\|26166, SKA3\|221150, ZFPM2\|23414, SLC6A15\|55117, SFTPA2\|729238, PKD1L2\|114780, LGALS9B\|284194, RORB\|6096, SMOX\|54498, EMILIN3\|90187, IGF1\|3479, KCNT2\|343450, CAMK2A\|815, DSG1\|1828, CYBRD1\|79901, ANKRD53\|79998, SCN4A\|6329, GALNT13\|114805, C4orf39\|152756, SLC8A3\|6547, KIF14\|9928, TWIST2\|117581, PTH1R\|5745, MMP11\|4320, FAM132A\|388581, LOC84931\|84931, CLEC4G\|339390, PROZ\|8858, PLA2G4E\|123745, C2orf58\|285154, INHBA\|3624, DCN\|1634, AR\|367, AKAP12\|9590, PABPC5\|140886, C13orf36\|400120, ASCL1\|429, TLX1NB\|100038246, TTYH1\|57348, LOC134466\|134466, ZNF471\|57573, NHSL2\|340527, FIGN\|55137, LOC283867\|283867, KIAA1257\|57501, C17orf96\|100170841, EXTL1\|2134, APCDD1L\|164284, SLC6A16\|28968, CYTL1\|54360, KCNK2\|3776, COL1A1\|1277, DKFZp779M0652\|374387, PAX4\|5078, BGN\|633, DPY19L2\|283417, LCN10\|414332, EFCAB1\|79645, HIST1H1E\|3008, STK33\|65975, L1CAM\|3897, CDO1\|1036, AOX1\|316, GAS1\|2619, CCDC80\|151887, HBA1\|3039, NANOS3\|342977, LOC100240735\|100240735, ZSCAN23\|222696, LEP\|3952, LOC389332\|389332, LRRC4C\|57689, CYP11A1\|1583, PRRG3\|79057, SFTPA1\|653509, RUNX1T1\|862, FAM55B\|120406, GRB7\|2886, RFPL1S\|10740, CPXM1\|56265, MGP\|4256, COL11A1\|1301, KRT20\|54474, NRG3\|10718, MMP7\|4316, FOXI2\|399823, SFTPD\|6441, LEF1\|51176, OLR1\|4973, COL10A1\|1300, C6orf176\|90632, MEOX2\|4223, TRDN\|10345, GPR98\|84059, NCAM2\|4685, CHI3L1\|1116, ALG1L\|200810, GRIK2\|2898, MFAP2\|4237, CORIN\|10699, GOLGA7B\|401647, FER1L4\|80307, TRIM29\|23650, GDF6\|392255, NOX4\|50507, IL10\|3586, SLC18A1\|6570, COMP\|1311, CTHRC1\|115908, TMEM132C\|92293, WISP1\|8840, UBD\|10537, ADAM12\|8038, GNGT1\|2792, CRYBB1\|1414, KRT23\|25984, FAP\|2191, ACSL6\|23305, KCNH4\|23415, ADAMTS12\|81792, NRK\|203447, A2BP1\|54715, C6\|729, KLHL34\|257240, DIO2\|1734, GJB4\|127534, PLAC1\|10761, NAT8B\|51471, SLC6A4\|6532, SPRR1B\|6699, SFRP2\|6423, REG1B\|5968, ZNF492\|57615, APOC2\|344, CEL\|1056, IGF2BP1\|10642, LRRC15\|131578, REG1A\|5967, SYN3\|8224, HULC\|728655, INS-IGF2\|723961, GSTT1\|2952 |
| 3 | Brown module | 173 | OTOP2\|92736, BEST4\|266675, CA7\|766, GUCA2B\|2981, TMIGD1\|388364, PYY\|5697, GCG\|2641, AQP8\|343, PKIB\|5570, CA1\|759, GLDN\|342035, SLC6A19\|340024, CDKN2BAS\|100048912, C2orf88\|84281, SLC25A34\|284723, INSL5\|10022, PRKG2\|5593, CLDN8\|9073, GUCA2A\|2980, ALPI\|248, SULT1A2\|6799, SLC4A4\|8671, CHAT\|1103, MMP28\|79148, MS4A12\|54860, CLDN23\|137075, C11orf86\|254439, FAM151A\|338094, CHGA\|1113, OSTBETA\|123264, SLC17A8\|246213, SMPDL3A\|10924, CLCA4\|22802, C14orf176\|643382, CA2\|760, TRPM6\|140803, ARL14\|80117, ANO5\|203859, TMEM72\|643236, C21orf88\|114041, SLC30A10\|55532, AGPAT9\|84803, CA4\|762, KRTAP13-2\|337959, AVPR1B\|553, USP2\|9099, UGT1A8\|54576, FAM135B\|51059, RNF152\|220441, CCDC68\|80323, CD177\|57126, HSD17B2\|3294, GBA3\|57733, ZG16\|653808, GDPD2\|54857, ACADS\|35, DAO\|1610, LDHD\|197257, NPY2R\|4887, GPT\|2875, MFSD4\|148808, SCNN1B\|6338, SLC17A4\|10050, NR3C2\|4306, DHRS9\|10170, B3GALT1\|8708, TSPAN7\|7102, ST6GALNAC6\|30815, SEMA6D\|80031, ABCC13\|150000, DHRS11\|79154, ENTPD5\|957, CDKN2B\|1030, SLC22A18AS\|5003, AKR1B10\|57016, EDN3\|1908, HSPH1\|10808, LOC646627\|646627, CBLN2\|147381, LEPREL1\|55214, SH2D7\|646892, CLCNKB\|1188, TFAP2B\|7021, HPGD\|3248, HBB\|3043, TMEM82\|388595, AHCYL2\|23382, CHST5\|23563, NR5A2\|2494, C6orf105\|84830, KCTD4\|386618, MRGPRX2\|117194, KLB\|152831, CCDC152\|100129792, CASR\|846, SULT1B1\|27284, TMCC3\|57458, SGK1\|6446, SDCBP2\|27111, B3GNT7\|93010, DISP2\|85455, BTNL8\|79908, MOGAT2\|80168, TRIM40\|135644, PADI2\|11240, SPINK5\|11005, HRASLS2\|54979, SULT1A1\|6817, B3GALT5\|10317, LOC643008\|643008, TMEM37\|140738, SLC16A9\|220963, LRRC19\|64922, HHLA2\|11148, PLA2G10\|8399, TTLL6\|284076, TUBAL3\|79861, PAPPA2\|60676, CIDEC\|63924, SLC30A8\|169026, CCL28\|56477, WDR78\|79819, PLAC8\|51316, MEP1B\|4225, TMEM171\|134285, PRKACB\|5567, ITM2C\|81618, HBA2\|3040, LANCL3\|347404, PPARGC1A\|10891, MYPN\|84665, SEPP1\|6414, CES3\|23491, SCNN1G\|6340, HRCT1\|646962, HSD11B2\|3291, PPYR1\|5540, SLC26A3\|1811, TSPAN1\|10103, BTNL3\|10917, APLN\|8862, PDE4C\|5143, FAM55A\|120400, ABCB11\|8647, P2RY4\|5030, C15orf48\|84419, SLC10A2\|6555, SCIN\|85477, GPA33\|10223, TAT\|6898, PCK1\|5105, ADH6\|130, GCNT3\|9245, XDH\|7498, GLRA4\|441509, CEACAM7\|1087, TBX10\|347853, CLDN14\|23562, CT62\|196993, LGR5\|8549, CREB3L3\|84699, SHOX\|6473, NRG1\|3084, EDN2\|1907, FAM95B1\|100133036, SLC15A1\|6564, MMP10\|4319, C10orf99\|387695, FABP1\|2168, MEP1A\|4224, LBP\|3929, PIGR\|5284, SHISA9\|729993 |
| 4 | Cyan module | 39 | CCBE1\|147372, PTGDR\|5729, ESM1\|11082, SOX15\|6665, P2RY1\|5028, OLFM3\|118427, TEX11\|56159, UGT2A3\|79799, AQP4\|361, KLHL1\|57626, CEP72\|55722, RICH2\|9912, GPT2\|84706, CCDC160\|347475, CHST9\|83539, KIT\|3815, LIX1\|167410, MOBP\|4336, HEATR7B2\|133558, UGT2B11\|10720, C17orf93\|360205, STK31\|56164, VSNL1\|7447, PCDH15\|65217, UGT1A9\|54600, SLCO1B3\|28234, TNNI3\|7137, NXPH4\|11247, SOX2OT\|347689, UNC93A\|54346, ADAMTS15\|170689, POU4F1\|5457, UGT2B7\|7364, NTRK2\|4915, ADAMTS19\|171019, NEUROG2\|63973, FGF20\|26281, SOX2\|6657, PLA2G2A\|5320 |
| 5 | Green module | 131 | FRMPD4\|9758, FMN2\|56776, RBM20\|282996, CDH3\|1001, LYNX1\|66004, CDKL1\|8814, KIF1A\|547, GPR119\|139760, CPM\|1368, ENTPD3\|956, LOC92659\|92659, ST8SIA1\|6489, EFHC2\|80258, FUT1\|2523, FBXO32\|114907, RNF43\|54894, DPF3\|8110, TMEM108\|66000, GALNT6\|11226, ATP11A\|23250, SLC5A6\|8884, UCHL1\|7345, SLC13A2\|9058, MGC14436\|84983, SLC22A17\|51310, SPTBN2\|6712, CDC25B\|994, UCN2\|90226, SHROOM4\|57477, SEMA6A\|57556, C14orf49\|161176, TRIB3\|57761, LOC100128239\|100128239, PPM1H\|57460, CGREF1\|10669, BMP2\|650, ACAN\|176, NEUROD1\|4760, FOXQ1\|94234, CST4\|1472, CHRNA1\|1134, MEX3A\|92312, DYNC1I1\|1780, CAPN13\|92291, MATN2\|4147, LMTK3\|114783, NOS1\|4842, GRIN2B\|2904, MEGF10\|84466, LOC148709\|148709, COL28A1\|340267, FJX1\|24147, FMO5\|2330, REEP1\|65055, AK5\|26289, MMP13\|4322, POU5F1B\|5462, CBX2\|84733, VWA2\|340706, SLC26A7\|115111, PI15\|51050, FGF18\|8817, DNMT3B\|1789, WNT3\|7473, WDR66\|144406, IRX5\|10265, SEZ6L\|23544, RECQL4\|9401, PTK7\|5754, RHPN1\|114822, SP6\|80320, IL5RA\|3568, CACNA1D\|776, CKB\|1152, FGF9\|2254, ProSAPiP1\|9762, FAM176A\|84141, IL11\|3589, IBSP\|3381, SULT4A1\|25830, LGI3\|203190, ZYG11A\|440590, EPYC\|1833, COL7A1\|1294, CAMKV\|79012, SEC14L4\|284904, LRRC6\|23639, TTR\|7276, NEB\|4703, C20orf46\|55321, TUBB2B\|347733, NPFFR1\|64106, PTP4A3\|11156, TG\|7038, CSF2\|1437, PAH\|5053, ST6GAL2\|84620, DCHS2\|54798, FGA\|2243, CLCNKA\|1187, COL27A1\|85301, KIF26B\|55083, TBX1\|6899, LOC145837\|145837, LRRN4\|164312, KIAA1875\|340390, DLX6AS\|285987, FGB\|2244, FOSB\|2354, GABRA2\|2555, MYBPHL\|343263, PIWIL1\|9271, LOC400696\|400696, DLX6\|1750, LY6G6D\|58530, SNORD116-4\|100033416, ABCC2\|1244, GZMB\|3002, SLC5A12\|159963, SLC38A3\|10991, ELF5\|2001, MMP3\|4314, CYP2B6\|1555, MMP1\|4312, DLX3\|1747, GPR81\|27198, ERP27\|121506, IL24\|11009, VENTX\|27287, SLC39A2\|29986, TBC1D3G\|654341 |
| 6 | Greenyellow module | 54 | MPZ\|4359, FEV\|54738, RHAG\|6005, STMN2\|11075, CNTN1\|1272, CD36\|948, CHGB\|1114, GSG1L\|146395, SCG3\|29106, KCNK3\|3777, PLD5\|200150, SYP\|6855, SNAP25\|6616, CORO2B\|10391, SEZ6\|124925, SCN3B\|55800, STAB2\|55576, KCNQ5\|56479, STXBP5L\|9515, CLDN11\|5010, C19orf30\|284424, LRRC55\|219527, SYT6\|148281, TRIM9\|114088, C1QTNF9\|338872, IL23A\|51561, DLGAP2\|9228, SORCS3\|22986, TMEM90A\|646658, SLC35F3\|148641, FMO2\|2327, PAK7\|57144, SLC5A11\|115584, CLDN16\|10686, KCNH8\|131096, CDK5R2\|8941, LOC255167\|255167, GJC3\|349149, SLITRK4\|139065, AP3B2\|8120, ASPHD1\|253982, FAM123C\|205147, FAM69C\|125704, CTXN2\|399697, RUNDC3A\|10900, STON1-GTF2A1L\|286749, KCNJ5\|3762, FBLL1\|345630, DMRTC1B\|728656, IL1F5\|26525, MATN3\|4148, NPR3\|4883, GLRA2\|2742, UCA1\|652995 |
| 7 | Grey module | 11 | LRRC7\|57554, GABRG1\|2565, SERPINA4\|5267, MGC16121\|84848, NDST4\|64579, DKK4\|27121, WNT16\|51384, BAAT\|570, HABP2\|3026, SLC7A9\|11136, FSTL5\|56884 |
| 8 | Magenta module | 94 | CWH43\|80157, HAPLN1\|1404, PTGS1\|5742, PDE7B\|27115, GPR12\|2835, SCD\|6319, CNGB1\|1258, ANLN\|54443, SLITRK1\|114798, CNTD2\|79935, KLHL35\|283212, CARD14\|79092, CCDC85A\|114800, GRHL3\|57822, GLYATL3\|389396, NKPD1\|284353, GCNT4\|51301, C6orf15\|29113, TAS2R38\|5726, C1orf105\|92346, EPHX4\|253152, KISS1\|3814, MAP2\|4133, CELSR3\|1951, AKR1C4\|1109, PLEKHG4\|25894, SULT2B1\|6820, ESPNP\|284729, C6orf223\|221416, GRHL1\|29841, KLK7\|5650, TH\|7054, GBX2\|2637, MCOLN2\|255231, CPNE4\|131034, SIM2\|6493, PP14571\|100130449, CCDC141\|285025, GABRB3\|2562, LY6G6C\|80740, GJA3\|2700, PRSS22\|64063, KRT6A\|3853, TRIM50\|135892, HEPHL1\|341208, KRT16\|3868, DGKB\|1607, NRADDP\|100129354, MMP8\|4317, PRSS33\|260429, TMEM211\|255349, CLCA2\|9635, C10orf81\|79949, PROM2\|150696, VWA3B\|200403, OR2W3\|343171, MSX1\|4487, TCN1\|6947, ADD2\|119, DIRAS2\|54769, PCSK9\|255738, STC1\|6781, SRMS\|6725, C8orf73\|642475, ISM2\|145501, C9orf169\|375791, GPC5\|2262, HFM1\|164045, FOXN1\|8456, PSORS1C2\|170680, SP8\|221833, ST8SIA6\|338596, TMPRSS13\|84000, C9orf70\|84850, UPK2\|7379, ATP12A\|479, SAA4\|6291, HOXB8\|3218, CYP26A1\|1592, MSX2\|4488, SPRR2A\|6700, SIX2\|10736, TACSTD2\|4070, ATP6V0A4\|50617, SPRR2D\|6703, CALCA\|796, GABRB2\|2561, PALM3\|342979, KRTAP3-1\|83896, PTPN20B\|26095, NBPF6\|653149, SAA2\|6289, TNNT1\|7138, REN\|5972 |
| 9 | Midnightblue module | 32 | CFD\|1675, EPB41L3\|23136, DSCAML1\|57453, JUB\|84962, XRCC2\|7516, SRL\|6345, CCDC150\|284992, FCGR2B\|2213, DMBX1\|127343, COLEC12\|81035, ATP6V0D2\|245972, TM4SF19\|116211, CTSW\|1521, KLRC1\|3821, NEUROG3\|50674, LY6H\|4062, SPTBN5\|51332, RNF165\|494470, MAP1LC3C\|440738, LHFPL4\|375323, KHDC1\|80759, STC2\|8614, NECAB2\|54550, DHRS2\|10202, CXCL11\|6373, PPBP\|5473, ZIC5\|85416, C14orf105\|55195, KLK10\|5655, PNPLA3\|80339, ZIC2\|7546, NPSR1\|387129 |
| 10 | Pink module | 97 | CFD\|1675, EPB41L3\|23136, DSCAML1\|57453, JUB\|84962, XRCC2\|7516, SRL\|6345, CCDC150\|284992, FCGR2B\|2213, DMBX1\|127343, COLEC12\|81035, ATP6V0D2\|245972, TM4SF19\|116211, CTSW\|1521, KLRC1\|3821, NEUROG3\|50674, LY6H\|4062, SPTBN5\|51332, RNF165\|494470, MAP1LC3C\|440738, LHFPL4\|375323, KHDC1\|80759, STC2\|8614, NECAB2\|54550, DHRS2\|10202, CXCL11\|6373, PPBP\|5473, ZIC5\|85416, C14orf105\|55195, KLK10\|5655, PNPLA3\|80339, ZIC2\|7546, NPSR1\|387129 |
| 11 | Purple module | 84 | SST\|6750, ABCG2\|9429, ETV4\|2118, DNASE1L3\|1776, LY6G6E\|79136, GPR15\|2838, LILRB5\|10990, MT1G\|4495, MT1E\|4493, MS4A10\|341116, TOMM34\|10953, TEAD4\|7004, LOC389791\|389791, LRRC8E\|80131, PVT1\|5820, GSG1\|83445, LY6G6F\|259215, C1orf170\|84808, PLEKHN1\|84069, IFITM1\|8519, SNORA39\|677821, SNORD1C\|677850, C8ORFK29\|340393, EVPL\|2125, PRDM12\|59335, RHEBL1\|121268, SLCO4A1\|28231, TM6SF2\|53345, CCNO\|10309, PRR7\|80758, MIR17HG\|407975, LOC388796\|388796, KRTAP5-1\|387264, PBX4\|80714, C20orf165\|128497, KLHL31\|401265, ANKRD13B\|124930, CPNE7\|27132, LOC286467\|286467, KRT9\|3857, WNT8B\|7479, PABPC1L\|80336, MT1L\|4500, CCDC78\|124093, FLJ41941\|100192420, PCSK1N\|27344, CYP2D7P1\|1564, KCNQ4\|9132, NSUN5P1\|155400, LIPC\|3990, C19orf45\|374877, C2orf48\|348738, IGFL4\|444882, R3HDML\|140902, TNS4\|84951, GABRE\|2564, RDH16\|8608, GDPD5\|81544, HAGHL\|84264, MLXIPL\|51085, NSUN5P2\|260294, PMFBP1\|83449, C2orf61\|285051, LOC150197\|150197, C2CD4A\|145741, KIR2DL4\|3805, MAPK15\|225689, TTC16\|158248, UNC5CL\|222643, SH2D5\|400745, HSF4\|3299, ALDH3B2\|222, RP1\|6101, TSPO2\|222642, ATHL1\|80162, FAM131C\|348487, HMX3\|340784, AKAP4\|8852, TNFRSF6B\|8771, TF\|7018, SLC6A20\|54716, MT1A\|4489, KRT39\|390792, B4GALNT4\|338707 |
| 12 | Red module | 126 | SCN9A\|6335, OTOP3\|347741, SRPX\|8406, EPHA7\|2045, IGFBP6\|3489, PRPH\|5630, RDH5\|5959, CRYBA2\|1412, UST\|10090, MAPT\|4137, FAM189A2\|9413, CNTNAP3\|79937, SLC17A7\|57030, GP2\|2813, CA14\|23632, FOXD3\|27022, RIMS4\|140730, NEFL\|4747, MT1X\|4501, IGSF11\|152404, INSM1\|3642, SLCO4C1\|353189, GSN\|2934, CDKL2\|8999, RND2\|8153, RASD2\|23551, HRK\|8739, OTX1\|5013, SYN2\|6854, LTK\|4058, DMRTA1\|63951, MT2A\|4502, MDFI\|4188, GRIA3\|2892, ULBP1\|80329, TMEM132B\|114795, HRASLS5\|117245, SYT5\|6861, CEACAM3\|1084, TMEM74\|157753, CCDC13\|152206, ASXL3\|80816, LRAT\|9227, APLP1\|333, SYT4\|6860, EGFL6\|25975, TMEM132A\|54972, XIRP1\|165904, FLT3\|2322, RAET1K\|646024, HTR3E\|285242, PEG10\|23089, RFX6\|222546, ULBP2\|80328, HTR3C\|170572, TMEM155\|132332, HPCAL4\|51440, SPTBN4\|57731, SIX1\|6495, RAB3B\|5865, KLC3\|147700, CLEC5A\|23601, TAS1R1\|80835, GNG13\|51764, NTSR1\|4923, C5orf46\|389336, HOXD13\|3239, TFR2\|7036, PDZD7\|79955, SLAMF9\|89886, MATN4\|8785, PTX3\|5806, TROAP\|10024, C19orf26\|255057, ZNF385B\|151126, GPR27\|2850, GPAT2\|150763, STK32A\|202374, NCAN\|1463, ELMOD1\|55531, GLS2\|27165, SNCB\|6620, PRRT4\|401399, SH2D6\|284948, ERBB4\|2066, KRT17\|3872, DUSP9\|1852, VEPH1\|79674, ANKRD33\|341405, FCN1\|2219, ACADL\|33, VWA5B2\|90113, ONECUT2\|9480, C18orf34\|374864, VNN2\|8875, AZGP1\|563, LRRC16B\|90668, FLJ42875\|440556, EEF1A2\|1917, DNAH2\|146754, TDGF3\|6998, PAEP\|5047, SH3TC2\|79628, TRIM55\|84675, HMX2\|3167, CCL8\|6355, MST1\|4485, PCDHAC1\|56135, HS3ST5\|222537, SCEL\|8796, CCK\|885, TDGF1\|6997, PRKCG\|5582, SERPINE1\|5054, CILP2\|148113, HOXC11\|3227, OSM\|5008, IL8\|3576, KLHL14\|57565, MARCO\|8685, GPR109B\|8843, CRABP1\|1381, MYEOV\|26579, PRSS21\|10942, H19\|283120, MUC5B\|727897 |
| 13 | Salmon module | 41 | PTPRZ1\|5803, LEMD1\|93273, IGSF10\|285313, KIAA1199\|57214, CALB2\|794, MAOB\|4129, C2orf70\|339778, COL4A6\|1288, DPP10\|57628, NEK5\|341676, SNTB1\|6641, FGF19\|9965, PHLDA1\|22822, TESC\|54997, KLK6\|5653, SLC4A11\|83959, KRT6C\|286887, GLT25D2\|23127, PGC\|5225, EN2\|2020, TRIM58\|25893, LRRTM1\|347730, ARMC4\|55130, SOSTDC1\|25928, CTNNA2\|1496, CSMD3\|114788, SUSD4\|55061, ROS1\|6098, LOC100127888\|100127888, KRT6B\|3854, NOTUM\|147111, HOMER2\|9455, TNNT2\|7139, DEFB1\|1672, SPRR1A\|6698, TMPRSS5\|80975, INHBB\|3625, CAPN6\|827, SPRR3\|6707, KAL1\|3730, APOH\|350 |
| 14 | Tan module | 44 | CNR1\|1268, RXRG\|6258, HLF\|3131, AGTR2\|186, GFRA3\|2676, CNTFR\|1271, NLGN4X\|57502, PPP1R1A\|5502, SFTA2\|389376, PCP4L1\|654790, GABRG2\|2566, SH3GL3\|6457, CNTNAP4\|85445, POU3F4\|5456, LOC389493\|389493, FSIP1\|161835, SVOP\|55530, SLC8A2\|6543, AXIN2\|8313, KCNJ14\|3770, BTBD16\|118663, KRT75\|9119, C10orf140\|387640, ZNF676\|163223, ZNF727\|442319, JPH3\|57338, SOHLH2\|54937, CATSPERG\|57828, SLC23A1\|9963, ODZ1\|10178, LMX1A\|4009, RAD51AP2\|729475, PKP1\|5317, GPR156\|165829, ZNF257\|113835, ZNF280A\|129025, MT1DP\|326343, AGT\|183, IRX6\|79190, GOLGA6L6\|727832, DSG3\|1830, MAGEA3\|4102, MAP7D2\|256714, NXF2\|56001 |
| 15 | Turquoise module | 623 | CLEC3B\|7123, PLP1\|5354, KCNIP4\|80333, ANGPTL1\|9068, CADM3\|57863, PRIMA1\|145270, MAMDC2\|256691, SCN7A\|6332, ATP1A2\|477, FAM107A\|11170, GCNT2\|2651, RSPO2\|340419, NRXN1\|9378, KIAA0408\|9729, GNG7\|2788, BMP3\|651, TMEM100\|55273, NGB\|58157, PI16\|221476, DPP6\|1804, NBLA00301\|79804, HRNBP3\|146713, TMEM35\|59353, PYGM\|5837, BCHE\|590, GRIK3\|2899, RYR3\|6263, CADM2\|253559, CMTM5\|116173, PDE2A\|5138, AFF3\|3899, RBM24\|221662, LOC572558\|572558, TACR2\|6865, NAP1L2\|4674, GNAO1\|2775, DVWA\|344875, CCL14\|6358, LDB3\|11155, NPTX1\|4884, ANK2\|287, ANGPTL7\|10218, SDPR\|8436, GREM2\|64388, EPM2A\|7957, LOC401093\|401093, NEGR1\|257194, GPM6A\|2823, MYOM1\|8736, LMO3\|55885, SSBP2\|23635, MYOC\|4653, PTN\|5764, RALYL\|138046, CHODL\|140578, NTN1\|9423, PLAC9\|219348, SOX10\|6663, SEMA3E\|9723, PDZD4\|57595, PDZRN4\|29951, PIRT\|644139, MYOT\|9499, TMEFF2\|23671, PMP2\|5375, SORCS1\|114815, CCDC69\|26112, GHR\|2690, MMP27\|64066, KIAA2022\|340533, RBPMS2\|348093, MASP1\|5648, TNXB\|7148, NFE2L3\|9603, FXYD1\|5348, NECAB1\|64168, HMP19\|51617, NLGN1\|22871, STMN4\|81551, PDK4\|5166, BAALC\|79870, C2orf40\|84417, HAND2\|9464, CFL2\|1073, ASB2\|51676, HSPB8\|26353, BAI3\|577, MAB21L1\|4081, SPHKAP\|80309, FOXF2\|2295, WISP2\|8839, PSD\|5662, KCNMB1\|3779, NCAM1\|4684, C1QTNF7\|114905, FAM129A\|116496, SOX9\|6662, FAM46B\|115572, POPDC2\|64091, NEBL\|10529, EIF4E3\|317649, CDH19\|28513, NKX2-3\|159296, LONRF2\|164832, SLC2A4\|6517, PGM5\|5239, ADCY5\|111, KCNMA1\|3778, LRRTM3\|347731, MORN5\|254956, GPER\|2852, PPP1R14A\|94274, BVES\|11149, PLN\|5350, FABP4\|2167, CASQ2\|845, C7orf58\|79974, CAND2\|23066, XKR4\|114786, NOVA1\|4857, PDE5A\|8654, CAP2\|10486, ADAMTSL3\|57188, RUNDC3B\|154661, WSCD1\|23302, MYOCD\|93649, TAGLN3\|29114, SNCG\|6623, FBXL22\|283807, GALR1\|2587, SCN4B\|6330, KIAA1644\|85352, KCNA4\|3739, PLIN4\|729359, JAM2\|58494, PHOX2B\|8929, PEG3\|5178, TGFBI\|7045, SCN2B\|6327, TCEAL2\|140597, SH3BGR\|6450, RNF150\|57484, PKNOX2\|63876, RGMA\|56963, CLDN5\|7122, CNGA3\|1261, TCEAL7\|56849, SPOCK3\|50859, BEND5\|79656, C2orf71\|388939, KY\|339855, CSRP1\|1465, SETBP1\|26040, RNF112\|7732, HOXD1\|3231, MAB21L2\|10586, HPSE2\|60495, FHL1\|2273, DNAJB5\|25822, MAPK4\|5596, DNER\|92737, BEX1\|55859, ATP1B2\|482, JPH2\|57158, DACT3\|147906, HSPB6\|126393, CDH18\|1016, KCNB1\|3745, GNG3\|2785, KRT27\|342574, C20orf200\|253868, PGM5P2\|595135, ROR1\|4919, DLG2\|1740, ANKS1B\|56899, ADHFE1\|137872, FCER1A\|2205, CAV1\|857, MUSTN1\|389125, CPEB1\|64506, TP53INP2\|58476, CTNND2\|1501, CDH10\|1008, HDAC9\|9734, CHRDL1\|91851, SLC5A7\|60482, ASB5\|140458, ADIPOQ\|9370, PGR\|5241, SPEG\|10290, ASTN1\|460, NRG2\|9542, FILIP1\|27145, ARL4D\|379, LGI4\|163175, LIMS2\|55679, TMOD1\|7111, NBEA\|26960, GRIK5\|2901, GTF2IRD1\|9569, CTNNA3\|29119, EPHA6\|285220, DAND5\|199699, MAPK10\|5602, RGS9\|8787, TPM1\|7168, RELN\|5649, PTCHD1\|139411, PPP1R12B\|4660, SMYD1\|150572, MACC1\|346389, CHRM2\|1129, SYNM\|23336, MRGPRF\|116535, RAB9B\|51209, GEFT\|115557, C9orf4\|23732, LMOD1\|25802, THRB\|7068, ELAVL4\|1996, AKAP6\|9472, SCN11A\|11280, MYLK\|4638, RGS2\|5997, MYT1L\|23040, CYS1\|192668, SNCA\|6622, FXYD6\|53826, PNCK\|139728, PGPEP1L\|145814, SVIL\|6840, SYNPO2\|171024, JPH4\|84502, HAND1\|9421, SLC7A14\|57709, KRT80\|144501, SCG2\|7857, C12orf53\|196500, ATP2B3\|492, TMEM59L\|25789, MEIS1\|4211, SLC28A2\|9153, ACTL6B\|51412, GAP43\|2596, INA\|9118, HSPB2\|3316, LRRN4CL\|221091, C6orf186\|728464, ADCY2\|108, GSTM2\|2946, RERG\|85004, REEP2\|51308, MPP2\|4355, CLIP3\|25999, MEIS2\|4212, KIAA1549\|57670, NEFM\|4741, ACSBG1\|23205, RASGEF1C\|255426, SCRG1\|11341, SALL2\|6297, LRRC3B\|116135, OLFM1\|10439, AGTR1\|185, TLX1\|3195, ADAM33\|80332, ATCAY\|85300, COX7A1\|1346, SLC7A5\|8140, OSR1\|130497, SLC27A6\|28965, C20orf166\|128826, CRYAB\|1410, NTRK3\|4916, RIC3\|79608, GRIA4\|2893, SERTAD4\|56256, ARPP21\|10777, C1orf95\|375057, ZNF536\|9745, NACAD\|23148, PCDH9\|5101, PRKAA2\|5563, TCF7\|6932, SECTM1\|6398, GALNTL1\|57452, TCEAL5\|340543, PHYHD1\|254295, PABPC1L2B\|645974, ST8SIA3\|51046, DARC\|2532, SPERT\|220082, MAP6\|4135, ANKRD35\|148741, WDR17\|116966, TMEM196\|256130, C14orf132\|56967, SPATA4\|132851, CELF4\|56853, DMD\|1756, FGF2\|2247, TACR1\|6869, AKAP7\|9465, ATRNL1\|26033, VIP\|7432, SLC16A12\|387700, ZDHHC22\|283576, PHOX2A\|401, NRSN1\|140767, LOC728264\|728264, MSRB3\|253827, COL21A1\|81578, AQP7\|364, PNLIPRP2\|5408, ENTPD8\|377841, ELAVL3\|1995, SGCA\|6442, SLITRK2\|84631, LOC541471\|541471, EVX2\|344191, ZNF229\|7772, MYL9\|10398, NPAS4\|266743, PLIN1\|5346, SORBS2\|8470, HSPB7\|27129, CNN1\|1264, CST2\|1470, FAM19A4\|151647, PRDM6\|93166, CHRNA3\|1136, KIRREL3\|84623, UNC80\|285175, FAM189A1\|23359, P2RX1\|5023, TRPV3\|162514, ITIH5\|80760, ATP2B4\|493, CEND1\|51286, ADAMTS8\|11095, KIAA1239\|57495, SLITRK5\|26050, GRIN2A\|2903, FXYD5\|53827, KCNA1\|3736, MYH11\|4629, DDX25\|29118, LRFN5\|145581, DPYSL5\|56896, PLA2G5\|5322, VIPR2\|7434, DDN\|23109, SLITRK3\|22865, SLC35F1\|222553, LOC284578\|284578, SV2B\|9899, OXTR\|5021, DUSP26\|78986, AOC3\|8639, ITGA7\|3679, SCARA3\|51435, C5orf40\|408263, C1orf133\|574036, FLNC\|2318, DOCK3\|1795, IL1RAPL1\|11141, THBS4\|7060, WBSCR17\|64409, SLC22A11\|55867, PIK3C2G\|5288, BHMT2\|23743, GRIN2D\|2906, KCNA5\|3741, KIF5A\|3798, MAOA\|4128, NKD2\|85409, ACTG2\|72, CHST8\|64377, DIRAS1\|148252, TNFRSF12A\|51330, TUSC5\|286753, GPR26\|2849, NAP1L3\|4675, SPARCL1\|8404, WNT9A\|7483, EPHA5\|2044, UCN3\|114131, MDGA2\|161357, UBE2QL1\|134111, NEXN\|91624, PDLIM3\|27295, LCN6\|158062, ABCB5\|340273, LRP8\|7804, C6orf168\|84553, CCL21\|6366, TTLL7\|79739, SYT9\|143425, FLRT1\|23769, PCDH10\|57575, C8orf85\|441376, PCDH7\|5099, CYP24A1\|1591, OR51E2\|81285, CPXM2\|119587, AMOTL1\|154810, DTNA\|1837, FAM163A\|148753, HCN4\|10021, TNS1\|7145, NEU4\|129807, ZNF677\|342926, RADIL\|55698, LTC4S\|4056, PPP2R2B\|5521, GRIA2\|2891, KL\|9365, LOC399959\|399959, SORBS1\|10580, RNF183\|138065, CHRNB4\|1143, NPPC\|4880, DSCR6\|53820, LOC283856\|283856, C8orf46\|254778, CACNA1H\|8912, CHRFAM7A\|89832, STRA6\|64220, PRDM8\|56978, FAM124A\|220108, TAGLN\|6876, STOX2\|56977, C1orf70\|339453, TPO\|7173, GPIHBP1\|338328, SFTPC\|6440, DBNDD1\|79007, TPM2\|7169, MFAP4\|4239, DPEP1\|1800, ADRB3\|155, CHRNA7\|1139, TMEM179\|388021, HTR4\|3360, CLU\|1191, TSPAN2\|10100, KCNF1\|3754, WBSCR28\|135886, ADRA1A\|148, C1orf182\|128229, CORO6\|84940, C6orf155\|79940, POU3F3\|5455, PRELP\|5549, KCTD8\|386617, CIDEA\|1149, ORC6L\|23594, CPA4\|51200, PTGER3\|5733, FAM163B\|642968, MYH16\|84176, FGF10\|2255, NKAPL\|222698, NAP1L6\|645996, GDNF\|2668, ALK\|238, C1orf180\|439927, LOC100190940\|100190940, ZCCHC12\|170261, DCAF12L2\|340578, BRCA2\|675, C15orf59\|388135, C14orf180\|400258, LGALS9C\|654346, FLNA\|2316, NPY\|4852, CLVS2\|134829, FAM150B\|285016, RET\|5979, RELL2\|285613, PENK\|5179, KCNIP1\|30820, CST1\|1469, C8G\|733, HSPB3\|8988, HKDC1\|80201, DES\|1674, C1orf135\|79000, SLC10A4\|201780, WNT7B\|7477, SLC18A3\|6572, HIPK4\|147746, MPPED2\|744, RSPO3\|84870, RPRM\|56475, SHISA2\|387914, SERPINA5\|5104, NGFR\|4804, UNC13C\|440279, LOC150622\|150622, LPO\|4025, NXPH3\|11248, JAKMIP3\|282973, PTPRN\|5798, ITGA8\|8516, C1orf114\|57821, CRTAC1\|55118, ARID3A\|1820, GPR88\|54112, PITX2\|5308, FAM40B\|57464, C10orf107\|219621, WSCD2\|9671, CNKSR2\|22866, C9orf24\|84688, LRRC36\|55282, FAM150A\|389658, STAC2\|342667, GPR22\|2845, RAB3C\|115827, GAD1\|2571, FLJ35024\|401491, LOC286002\|286002, SMPX\|23676, ZNF835\|90485, CD207\|50489, NUDT10\|170685, PTGIS\|5740, KRTAP5-4\|387267, LOC100216001\|100216001, MYH2\|4620, DUSP5P\|574029, PRDM13\|59336, DACH1\|1602, OTC\|5009, ARHGAP36\|158763, PSAT1\|29968, ZNF483\|158399, C1QTNF4\|114900, LOC154822\|154822, CELP\|1057, SHH\|6469, CASQ1\|844, FEZF1\|389549, ADRA1D\|146, RAMP1\|10267, DNAH5\|1767, FOSL1\|8061, EGF\|1950, XKRX\|402415, UGT3A2\|167127, HTR2B\|3357, CLDN9\|9080, NME5\|8382, LRRTM4\|80059, WT1\|7490, HOXB13\|10481, PRAC\|84366, FAM184A\|79632, CPA6\|57094, PNPLA1\|285848, KRT7\|3855, DCX\|1641, PRND\|23627, CDH22\|64405, HBG1\|3047, PCP4\|5121, IGFL1\|374918, RAET1L\|154064, LOC440356\|440356, CRLF1\|9244, MT3\|4504, STL\|7955, TIMD4\|91937, DMGDH\|29958, F7\|2155, PCDHA3\|56145, SSTR5\|6755, CAMK2B\|816, HAVCR1\|26762, HTR1D\|3352, NKD1\|85407, SP5\|389058, LOC151174\|151174, CARTPT\|9607, CXCL1\|2919, NXPH1\|30010, CLGN\|1047, CXCL3\|2921, MYOM3\|127294, SLC6A2\|6530, CA9\|768, GLYATL1\|92292, SPACA3\|124912, CLDN2\|9075, C21orf34\|388815, COL22A1\|169044, REG3A\|5068, F2\|2147, KRT14\|3861, C7orf52\|375607, IL17A\|3605, GDF5\|8200, ALDH1A2\|8854, ORM1\|5004, SLC26A9\|115019, ASGR1\|432, HOTAIR\|100124700, SLC13A3\|64849, TBX20\|57057, MAT1A\|4143, OBP2B\|29989, C14orf53\|440184, NLRP2\|55655, KCNV1\|27012, COL9A3\|1299, KNG1\|3827, PTF1A\|256297, C9orf71\|169693, DSC3\|1825, BMP7\|655, WDR72\|256764, APOB\|338 |
| 16 | Yellow module | 165 | KRT24\|192666, SPIB\|6689, PCSK2\|5126, LIFR\|3977, LGI1\|9211, AADACL2\|344752, ABCA8\|10351, VSTM2A\|222008, MAL\|4118, DHRS7C\|201140, MMRN1\|22915, ABI3BP\|25890, GLP2R\|9340, RERGL\|79785, FGFBP2\|83888, MT1H\|4496, ADH1B\|125, PKHD1L1\|93035, KHDRBS2\|202559, CNTN2\|6900, LGALS2\|3957, GFRA1\|2674, TMEM220\|388335, P2RX2\|22953, IL1R2\|7850, MUSK\|4593, PLCL2\|23228, CTSG\|1511, SLC6A6\|6533, FER1L6\|654463, FAM19A2\|338811, SEC14L5\|9717, RGS13\|6003, CPNE8\|144402, SI\|6476, TUSC3\|7991, SYT10\|341359, UNC5D\|137970, CAV3\|859, TPH1\|7166, CD79B\|974, FAM23A\|653567, FOXP2\|93986, NNAT\|4826, HEPACAM2\|253012, ANPEP\|290, SYNGR1\|9145, MADCAM1\|8174, ASPG\|374569, CR2\|1380, VPREB3\|29802, MTHFD1L\|25902, TNFRSF13B\|23495, CD22\|933, FCRLA\|84824, TOX\|9760, KIAA1683\|80726, PCDH11X\|27328, MST1P9\|11223, ST6GALNAC3\|256435, FGFR2\|2263, FCER2\|2208, MS4A1\|931, GPR44\|11251, STAP1\|26228, SNAP91\|9892, CD79A\|973, KIAA1045\|23349, ATP13A4\|84239, SCN3A\|6328, CCL19\|6363, TCL1A\|8115, ITGB1BP3\|27231, EFNA5\|1946, NCRNA00092\|100188953, COL19A1\|1310, BLK\|640, FLJ43390\|646113, LOC645323\|645323, C1orf173\|127254, GSTM3\|2947, SNTG2\|54221, C1orf186\|440712, CHP2\|63928, HEMGN\|55363, CXCR5\|643, CMAH\|8418, TLR10\|81793, SCUBE2\|57758, PDE9A\|5152, GRIA1\|2890, CXCL13\|10563, HTR3A\|3359, KIAA1751\|85452, HMGCLL1\|54511, ZIK1\|284307, UGT1A1\|54658, BARX2\|8538, BEND4\|389206, CHAD\|1101, CLEC4M\|10332, NEURL\|9148, HEPACAM\|220296, ERVFRDE1\|405754, CD19\|930, MYADML2\|255275, C4orf7\|260436, CNR2\|1269, EPHA4\|2043, FLJ37543\|285668, SERPINA9\|327657, LOC100128164\|100128164, SGSM1\|129049, CBFA2T3\|863, KIAA0125\|9834, FCRL2\|79368, AICDA\|57379, CLEC17A\|388512, C16orf54\|283897, FCRL4\|83417, ABCA10\|10349, SLC16A7\|9194, CYP4B1\|1580, NKX2-2\|4821, SPTLC3\|55304, PDX1\|3651, FCRL1\|115350, KCTD12\|115207, C14orf64\|388011, SPON1\|10418, CFC1B\|653275, C13orf30\|144809, ACSM1\|116285, FCRL3\|115352, CYP3A4\|1576, MYO3A\|53904, PAX5\|5079, KLRF1\|51348, AGR3\|155465, SSTR1\|6751, CUX2\|23316, RSPO1\|284654, KCNB2\|9312, TBX15\|6913, AFF2\|2334, FAM129C\|199786, TNFRSF13C\|115650, CHRM1\|1128, MS4A8B\|83661, BFSP2\|8419, CAMP\|820, TMPRSS3\|64699, ATP2B2\|491, ST6GALNAC1\|55808, ARX\|170302, FAM3B\|54097, KBTBD12\|166348, HMGCS2\|3158, SRPX2\|27286, TPSD1\|23430, CACNA1B\|774, TMEM229A\|730130, NELL1\|4745, BRSK2\|9024, CXCL5\|6374 |
